# Acknowledgment to Reviewers of *Molecules* in 2020

**DOI:** 10.3390/molecules26030565

**Published:** 2021-01-22

**Authors:** Molecules Editorial Office

**Affiliations:** MDPI AG, St. Alban-Anlage 66, 4052 Basel, Switzerland

Peer review is the driving force of journal development, and reviewers are gatekeepers who ensure that *Molecules* maintains its standards for the high quality of its published papers. Thanks to the cooperation of our reviewers, in 2020, the median time to first decision was 13 days and the median time to publication was 32 days. The editors would like to express their sincere gratitude to the following reviewers for their precious time and dedication, regardless of whether the papers were finally published:
A. Bekhit, Alaa El DinLončar, NikolaAandres-Mach, MartaLončarević-Vasiljković, NatašaAav, RiinaLondergan, CaseyAbarbri, MohamedLondoño López, Martha ElenaAbashev, George G.Long, BrianAbate, LorenzoLong, YuchenAbba, Mohammed L.Longhi, GiovannaAbballe, AnnalisaLongo, AngelaAbbate, SergioLongo, Maria A.Abbehausen, CamillaLongo, PasqualeAbbrent, SabinaLongo, SandroAbbruzzetti, StefaniaLongo, ValentinaAbd El Hakim, YasminaLönnberg, HarriAbd El-Hack, Mohamed E.Loontjens, Ton J. A.Abdala, AhmedLopes Teixeira, GersonAbdallah, ShaabanLopes, Adelino V.Abdel-Rahman, Laila H.Lopes, AnaAbdel-Razek, AhmedLopes, FranciscaAbdelwahed, SamehLopes, GracilianaAbdullah, Oday IbraheemLópez Nicolás, Jose ManuelAbe, NaohitoLopez Rodilla, Jesus M.Abebe, WorkuLópez Romero, Juan M.Åberg, Daniel N.López, ConcepciónAbollino, OrnellaLopez, Jose CristobalAbramova, Tatyana V.López, Kelly Johana FigueroaAbramovich, HaimLópez, LluviaÁbrányi-Balogh, PéterLopez, VictorAbrunhosa, LuísLopez-Camarillo, CesarAbugri, Daniel A.López-Cornejo, PilarAbuin, GracielaLopez-Jaramillo, Francisco JavierAbu-Reidah, Ibrahim M.Lopez-Linares, FranciscoAbushouk, AbdelrahmanLópez-Linares, Juan CarlosAcero, NuriaLópez-López, José A.Aceto, MaurizioLópez-Lorente, Ángela InmaculadaAchelle, SylvainLópez-Salas, NievesAchinivu, EzinneLopez-Serrano, JoaquinAchour, BrahimLopez-Viñas, EduardoAci-Sèche, SamiaLoppinet, BenoitAčkar, ĐurđicaŁopusiewicz, ŁukaszAcker, JörgLordan, RonanAcquah, CalebLorenc-Koci, ElżbietaAcquaviva, RosariaLorentz, AxelÁdám, TölgyesiLorenzetti, StefanoAdam, TomášLorenzo, Jose M.Adamakis, Ioannis-DimosthenisLorenzo, JuliaAdamcová, DanaLorenzo-Morales, JacobAdamczyk, GretaLoret, Erwann P.Adamczyk-Woźniak, AgnieszkaLoretz, BrigittaAdamkova, AnnaLorite, PedroAdamo, CarloLorkiewicz, Pawel K.Adamopoulos, StergiosŁoś, SzymonAddepalli, BalasubrahmanyamLosada-Perez, PatriciaAdebo, OluwafemiLosantos, RaúlAdelberg, JefferyLoschenov, Victor B.Adewole, DeborahLosurdo, GiuseppeAdi, Santoso Cornelia MelindaLotina-Hennsen, BlasAdil, MohdLou, ChenguangAdil, Syed FarooqLou, HongxiangAdjé, FélixLouarn, EssylltAdly, Frady G.Louda, PetrAdt, IsabelleLouie, StaceyAfantitis, AntreasLourenço, AnaAfarinkia, KamyarLourenço, AnáliaAfify, Ahmed SabryLourenço, Gustavo JacobAfonin, KirillLouvaris, ZafeirisAfonso, Carlos M.Love, JasonAfseth, Nils KristianLoveridge, JoelAgamennone, MariangelaLow, NicholasAganovic, KemalLowell, AndrewAgartan, LutfiLowery, JonathanAgbossou-Niedercorn, FrancineLoza-Coll, Mariano AndresAgeitos, Jose ManuelLoza-Mejía, Marco A.Agerbirk, NielsLoza-Mejía, Marco AntonioAggarwal, Bharat B.Lozano, Jose ManuelAggeli, Ioanna-KaterinaLozano-Gorrin, Antonio D.Agha, RamsyLozinsky, VladimirAghazada, SadigLu, Chih-HaoAgić, DejanLu, Chi-YuAgnieszka, SzopaLu, Chung-kuangAgòcs, AttilaLu, JianqinAgosta, LorenzoLu, JingzhiAgostino, MarkLu, Mei-ChinAgrawal, AlokLu, MengAgrawal, DineshLu, PengtaoAguayo, Maria GracielaLu, QingyiAguiar, AndréLu, QuanlongAguilar, EnriqueLu, ShaoyongAguilar-Bolados, HectorLu, ShuiyuAguilar-Caballos, María PazLu, WenchaoAguiló-Aguayo, NoemíLu, XiaoAguirre, Nestor F.Lu, XuejunAgusa, TetsuroLu, YiAgustin, DominiqueLu, YujiaoAgustin, Melissa B.Lu, YuyunAhamed, MuneerLu, Zhan-HuiAhlenstiel, ChantelleLubawy, JanAhluwalia, AmritaLubin-Germain, NadègeAhmad, JavedLubowiecka, IzabelaAhmad, KhurshidLucaciu, Constantin MihaiAhmad, ZulfiqarLucarini, SimoneAhmadi, MojtabaLucas, Andrew T.Ahmadivand, ArashLucas, GuilhermeAhmed, AlauddinLucas, Heather R.Ahmed, HafizLucena, RafaelAhmed, KhalilLucenti, ElenaAhmed, SaveerLuchini, ClaudioAhmed, Sheikh AliLucía Z., Flores-LópezAhmedova, AnifeLučić, BonoAhn, Hee-ChulŁuczaj, WojciechAhn, Ji-YoungLuddi, AliceAiba, YuichiroLudwiczuk, AgnieszkaAiello, FrancescaLugnier, ClaireAiello, GildaLugo, Jesus EduardoAimanianda, Vishu KumarLui, Edmund Man KingAiraksinen, AnuLuís, ÂngeloAires, AlfredoLuis, Santiago V.Aitken, R. AlanLuisetto, RobertoAjay, Amrendra K.Luisi, RenzoAjay, AshokLuiz Dotto, GuilhermeAkabayov, BarakLujanienė, GalinaAkad, FouadLukáč, MilošAkbar, MohammedLukasik, RafalÅkerström, BoŁukasz, MarczakAkhgari, AmirLukaszewicz, MarcinAkhrem, IrenaLukaszkowicz, KrzysztofAkimoto, SeijiLuke, WhileyAkita, SadanoriLukić, IgorAkitsu, TakashiroLukinac Čačić, JasminaAkiyama, HisashiLukinac, JasminaAkopova, TatianaLukinavicius, GrazvydasAkurathi, GopalakrishnaLukšič, MihaAlaimo, AlessandroLukyanov, Pavel A.Alam, Arif U.Luliński, SergiuszAlam, Mohammad AbrarLuna Maldonado, AurelioAlam, ToddLuna Moreno, DonatoAlander, JarmoLuna, DiegoAlarcon, JulioLundin, Jeffrey G.Alarif, Walied MohamedLung, Shiu CheungAlbalat, AmayaLungu, CristianAlbano, GianluigiLunov, OlegAlbarracín, Sonia L.Lunter, DominiqueAlbeck, AmnonLuo, DanmengAlbericio, FernandoLuo, HongliangAlbero, JosepLuo, JinAlbertolle, Matthew E.Luo, KuiAlbillos, AlmudenaLuo, RongcongAlbiniak, PhilipLuo, WentaiAlbuquerque, HélioLuo, XiaomeiAlcantara, AndresLuo, YanhuaAlcaraz, MiguelLuo, ZishengAlcarde, Andre RicardoLupan, AlexandruAlcaro, StefanoLuppi, BarbaraAlcolea Palafox, MauricioLupu, StelianAlcolea, VeronicaLusczek, Elizabeth R.Alcoutlabi, MatazLushchekina, SofyaAldred, KatieŁuszczki, JarogniewAldrich-Wright, JaniceLutfy, KabirullahAlduina, RosaLutter, ErikaAl-Dujaili, EmadLutz, MartinAlegria, Elisabete C.B.A.Luu, Hung N.Aleixandre, ManuelLuz, Rita De Cássia SilvaAlejandro-Martín, SergueiLuzi, FrancescaAleksandrov, AlexeyLuzina, OlgaAlekseeva, AnnaLuzzio, FredAlen, FranciscoLv, KangleAleshin, AndreyLvova, LarisaAlessandro, CecconelloLyashchenko, SergeAlessio, NicolaŁyczakowski, JanAlexa-Stratulat, TeodoraŁyczko, JacekAlexis, FrankLykidis, CharalamposAl-Fatimi, MohamedLyon, R. E.Alfei, SilvanaŁyszczek, RenataAlgieri, VincenzoLyu, HailongAlguacil, FranciscoLyukmanova, Ekaterina N.Al-Horani, Rami A.M. Harandi, VahidAli, Hayssam M.M. Vorob’ev, MikhailAli, NaserMa, Chih-MingAli, RameezMa, CongAli, ShafaqatMa, JunfengAliabadian, EhsanMa, JunyingAliaño González, María JoséMa, LiangAlikhani, EsmailMa, LongAlina, Brzeczek-SzafranMa, QianliAlipieva, KalinaMa, QingyongAl-Khrasani, MahmoudMa, XiaoliAlkorta, IbonMa, XueyingAllais, FlorentMa, YongAllan, SandraMa, YufeiAllegra, MarioMa, YupingAllegro, GianlucaMa, YurongAllen, AndrewMa, ZhenAllen, Bruce G.Ma, ZhengxinAllen, Norman S.Maász, GáborAllen, Sean DavidMacaluso, RobertoAllwood, Daniel M.Maccagno, MassimoAl-Maharik, NawafMaccallini, CristinaAlmajano, María PilarMacchi, BeatriceAlmeida Jr., HumbertoMacdonald, ThomasAlmeida Júnior, José HumbertoMacGillavry, Harold D.Almeida, AdelaideMach, MojmirAlmeida-Paes, RodrigoMachado, DianaAlmerico, Anna MariaMachado, FabricioAlmiro De Almeida, AgostinoMachado, Michel MansurAlmogi-Hazan, OsnatMachado, RaulAl-Mrabeh, AhmadMachetti, FabrizioAlonso, Ana P.Machrafi, HatimAlonso, José L.Macías, RamónAlonso-Moreno, CarlosMaciej, StrzemskiAlonso-Vante, NicolasMaciejewska, DorotaAl-Qurayshi, ZaidMaciel, Maria Inês SucupiraAl-Rimawi, FuadMaciuk, AlexandreAlshbool, Fatima Z.Mack, JohnAltamimi, MohammadMackereth, CameronAltamirano-Bustamante, Myriam M.MacKinnon, NeilAltemimi, AmmarMaçôas, ErmelindaAltieri, FabioMacone, AlbertoAltmeppen, HermannMacreadie, IanAltomare, CosimoMączka, MirosławAltucci, LuciaMączka, WandaAlugubelly, NavathaMączyński, MarcinAluicio-Sarduy, EduardoMadala, Hanumantha RaoAlvarado-Lassman, AlejandroMadej, KatarzynaAlvarez Fernandez, Maria AntoniaMadhukar, BurraAlvarez, AlejandraMadhyastha, RadhaAlvarez, Ana I.Madine, JillÁlvarez, EleuterioMadka, VenkateshwarAlvarez-Idaboy, Juan RaùlMadonov, PavelAlvarez-Parrilla, EmilioMadrid, AlejandroAlvarez-Ramirez, JoseMadsen, Steen J.Álvarez-Suarez, José M.Madureira, Patrícia A.Alves, Carla SophiaMadurga, SergioAlves, Carlos RobertoMady, Carlos Eduardo KeutenedjianAlves, FranciscoMady, Mohamed F.Alves, LuisMadzhidov, TimurAlves, MarcoMaeda, HayatoAlves, Wagner A.Maehara, ShojiAmadio, EmanueleMaeng, Han-JooAmanda, LumsdenMaeno, ZenAmano, TomokoMaes, EvelyneAmara, NeriMaestri, ElenaAmaral, Joana S.Maestro, MiguelAmarowicz, RyszardMaffei, FrancescaAmato, FeliceMaffei, GianlucaAmato, JussaraMaffia, MicheleAmenabar, MaximilianoMafu, SibongileAmer, BasharMaga, GiovanniAmer, HassanMagalhães, Geraldo S.Amero, CarlosMagalhães, SolangeAmi, DilettaMagdalena, WoźniczkaAmigo, LourdesMaGee, DavidAmigo-Benavent, MiryamMaggi, FilippoAmii, HidekiMagiatis, ProkopiosAmillis, SotirisMagkoev, Tamerlan T.Amine, AzizMagni, MirkoAmini, HajarMagoulas, GeorgeAminin, DmitryMagrassi, LorenzoAminnaji, MortezaMagrioti, VictoriaAmiri, EsmaeilMagrone, TheaAmmazzalorso, AlessandraMague, Joel T. Amo Ochoa, PilarMagyari, KlaraAmorati, RiccardoMahapatra, Ajit K.Amores Arrocha, AntonioMahar, RohitAmorim, Maria JoaoMahasenan, KiranAmoroso, RosaMahata, Manoj KumarAmovilli, ClaudioMahato, NeelimaAmr, Abd El-Galil E.Mahdavi, MahboobeAn, FeifeiMahdavian, ElaheAn, HyosungMaher, PamelaAn, QuanfuMahmudov, Kamran T.An, YuMaia, CláudioAna María, Zamora MartínezMaicas, SergiAnand, Jessica P.Maidaniuc, AndreeaAnandhan, AnnaduraiMaidannyk, ValentynAnanieva, ElitsaMaier, CameliaAnanthakrishnan, Soundaram JeevarathinamMaietta, SaverioAnconi, CleberMaietti, AnnalisaAncuceanu, RobertMainardi, MarcoAnděra, LadislavMaioli, SilviaAnderbrant, OlleMair, Lamar O.Andersen, PålMaireles Torres, Pedro JesusAnderson, Harry L.Maisetta, GiuseppantonioAndicsová Eckstein, AnitaMaixent, Jean MichelAndjelkovic, Anuska V.Maj, MalgorzataAndjelkovic, MirjanaMajcher, Malgorzata A.Andolfi, AnnaMajdan, MarekAndrade Cetto, AdolfoMajewski, MichałAndrade, Eugénia DeMajewski, PawelAndrade, José CarlosMajewski, SebastianAndrae, DirkMajireck, MaxAndrea, MahnMajkowska-Pilip, AgnieszkaAndreana, PeterMajolino, DomenicoAndreasen, RasmusMajtan, JurajAndrei, SandaMajumder, AvisekAndreou, ChrysafisMajumder, KaustavAndreu, InmaculadaMakabe, HidehumiAndreu, IreneMakarevich, PavelAndreuzzi, EvaMakareviciene, VioletaAndrews, Andrew J.Makarov, IgorAndrews, DavidMakarova, KaterinaAndrianov, AlexanderMakarska-Bialokoz, MagdalenaAndrić, FilipMakaryan, TaronAndruh, MariusMaker, GarthAndrysic, ZdenekMaki, YasuyukiAndrzejewski, KryspinMakino, ToshiakiAnedda, RobertoMakishima, MakotoAneta, PanuszkoMakky, AliAngel, Sergio O.Makosza, MieczyslawAngelella, GinaMakowski, MariuszAngeletti, MauroMaksimchuk, NataliyaAngeli, AndreaMaksimov, Anton LvovichAngelica De Oliveira, GomesMaksimowski, PawełAngelici, GaetanoMakunga, Nokwanda P.Angelini, PaolaMakvandi, PooyanAngelino, DonatoMalaguarnera, MarianoAngelov, BorislavMalaguarnera, MicheleAngelov, GeorgeMalakhov, DmitriAngelov, Orlin I.Malandrino, GraziellaAngelova, AngelinaMalapelle, UmbertoAngelova, Violina T.Malawska, BarbaraAnghel, AndreiMaldonado Hodar, Francisco JoséAngiolella, LetiziaMaldonado, José-LuisAngius, FabrizioMałecka, MagdalenaAngkawijaya, Artik ElisaMalek, KamillaAnibal, JaimeMalemud, Charles J.Aniceto, NatáliaMalenović, AnđelijaAniello, FrancescoMales, ZeljanAnis, BadawiMalfa, Giuseppe AntonioAnisimova, IrinaMalfeito-Ferreira, ManuelAnisoara Peptu, CatalinaMali, GregorAnitescu, CosminMalik-Gajewska, MagdalenaAnjos, OféliaMalińska, DominikaAnni, MarcoMalitesta, CosiminoAnnibal, AndreaMalkin, AlexanderAnnor, GeorgeMallah, TalalAnraku, MakotoMallamace, DomenicoAnsari, D. Mohd. AzamMallat, ArianeAntal, DianaMalliavin, ThérèseAntal, IstvánMallipeddi, Prema LathaAnthamatten, MitchellMallouchos, AthanasiosAnthemidis, AristidisMallow, OleAnticó, EnriquetaMalmström, LarsAntoce, Arina OanaMalucelli, GiulioAntoine, RodolpheMalvano, FrancescaAntolak, HubertMalwal, Satish R.Anton, HalinaMaly, PavelAntoni, GunnarMalzert-Fréon, AurélieAntoniassi, RosemarMamat, ConstantinAntoniewska, AgataMamba, BhekieAntonino, FamulariMamidi, NarmidiAntônio Casagrande, GleisonMammeri, FaynaAntonio Malfa, GiuseppeManabe, KeiAntonov, Alexander S.Manabe, ShinoAntonov, LiudmilManabe, YukiAntunes, AlexandraManagò, StefanoAntunes, DulceManakhov, AntonAnugwom, IkennaManca, Maria LetiziaAnzenbacher, PavelMancianti, FrancescaAoki, DanMancini, InesAoyagi, TakaoManco, GiuseppeApetrei, ConstantinMancuso, RaffaellaApopei, Andrei IonutMandard, StéphaneAppelhans, DietmarMandati, VinayAppell, MichaelManders, PeggyAppendino, GiovanniManderville, Richard A.Aprea, EugenioMandoli, AlessandroAprodu, IulianaMandrioli, RobertoAquaro, StefanoManera, ClementinaAquilano, DinoManes, JordiAquino, AndreaManetti, DinaArabski, MichałManetti, MirkoArakawa, KenjiManfredini, StefanoArand, MichaelMangani, StefanoArango, Rachel A.Maniam, SubashaniAraniciu, CătălinManian, Avinash P.Araújo, EvandoManicardi, AlexAraújo, João M. M.Manini, PaolaAraujo, Maria E. M.Manivannan, NadarajahAraujo, PedroMannari, VijayAraújo, Thaíse GonçalvesMannello, FerdinandoAraya, Samuel SimonManner, Virginia W.Araya-Maturana, RamiroMannina, LuisaArce, Pedro F.Mannino, GiuseppeArchibald, SteveMannino, SaverioArciello, AngelaMANNO, Daniela ErminiaArcone, RosariaManocha, GunjanArczewska, MartaManos, ManolisArdévol, AnnaManouras, TheodorosAreche, CarlosManoury, EricAremu, Adeyemi OladapoManousi, NataliaAresta, AntonellaMansell, PeterArgentieri, Maria PiaMansi, RosalbaArguelles, SandroMansikkamäki, AkseliArias Santiago, Salvador AntonioMansuri, ShahidAricò, FabioMantell, CasimiroAriga, KatsuhikoMantell, LinArinstein, ArkadiManthy, JohnArion, VladimirMantione, DanieleArisawa, MiekoMäntylä, ElinaArmand, MartineMantzioris, EvangelineArmanini, DecioMantzourani, IoannaArmenta, SergioMantzouridou, FaniArmijo, Juan FranciscoManyes, LaraArmitage, BruceManzano, Blanca R.Armitage, Ruth AnnManzano, RaquelArnaboldi, SerenaManzhos, SergeiArnaouteli, SofiaManzotti, GloriaArnason, JohnMao, YuanbingArnaut, Luís GuilhermeMao, ZongwanArnesano, FabioMarakos, PanagiotisAronica, Laura AntonellaMaráková, KatarínaArora, NehaMaramai, SamueleArosio, PaoloMarangon, MatteoArrieta, MarinaMarangoni, AlejandroArrieta-Baez, DanielMaranzana, AndreaArrigo, RossellaMarasco, DanielaArrizon, JavierMarat-Mendes, RosaArroo, RandolphMaraveas, ChrysanthosArruebo, ManuelMarbacher, SergeArscott, SteveMarc, GabrielArsene, Andreea LetiţiaMarcal, HelderArshad, AdeelMarceau, FrançoisArteaga, Jesús FernándezMarchetti, AlessandraArtem’ev, Alexander V.Marchetti, FabioArtyukhin, AlexMarchetti, MarioArugula, Mary AnithaMarchetti, MartaArunachalam, PrabhakarnMarchetti, NicolaArvanitis, AntoniosMarchev, AndreyAryal, Uma K.Marcinčáková, DanaAsadipooya, KamyarMarciniec, KrzysztofAsadzadeh, BehnazMarcinkowski, ŁukaszAsahara, HaruyasuMarco, FranciscoAsakawa, YoshinoriMarco-Contelles, Jose LuisAsamizu, ShumpeiMarcos, MarcosAsano, RyutaroMarcos, Paula M.Asaro, FiorettaMarcu, Ioan CezarAscheri, JoseMaréchal, Jean-DidierAscrizzi, RobertaMarek, KieliszekAsencios, Yvan J. O.Marengo, BarbaraAsfin, Ruslan E.Marengo, MauroAshley, JonMareș, MihaiAshour, Mohamed LotfyMaresca, MarcAshpole, Nicole M.Marfin, YuriyAshton, TrentMargarucci, SabrinaAsín, LauraMargheritis, EleonoraAspatwar, AshokMărghitaş, Liviu A.Asquith, Christopher R. M.Margiolaki, IreneAssfalg, MichaelMarhuenda-Egea, Frutos CarlosAstiasaran, IciarMaría A., PerilloAstolfi, AndreaMaria Teresa, FrangipaneAta, AtharMaria, Teresa M. R.Ata, SeisukeMarian, BrigitteAtaide, Janaína ArtemMariani, AlessandroAtanasov, Atanas TodorovMariani, StefanoAtanasov, VasilMariani-Costantini, RenatoAtanassova, MariaMariappan, KadarkaraisamyAtanassova, StefkaMarić, MilanAtawia, Reem T.Marichev, KostiantynAthanassopoulos, ConstantinosMarin, Jose J. G.Atobe, MasakazuMarin, LuminitaAtochin, DmitriyMarin, Ricardo MallaviaAtrián-Blasco, ElenaMarina, María LuisaAttia, Nour F.Marinescu, MariaAttri, PankajMarini, ElisabettaAtuchin, VictorMarini, FedericoAturki, ZeinebMarini, Herbert RyanAuberon, FlorenceMarino, AngelaAuffinger, PascalMarinova, VeraAugustyniak, AdrianMarius, BodorAugustyniak, DariaMark S., JohnsonAuh, Joong-HyuckMarkiewicz, Wojciech T.Auladell, CarmeMarkopoulou, OlgaAullon, GabrielMarkou, GiorgosAuriemma, GiuliaMarkova, SvetlanaAusra, SipailieneMarkova-Car, Elitza P.Au-Yeung, Ho YuMarković, KsenijaAvarvari, NarcisMarkovitsi, DimitraAvato, PinarosaMarkowicz-Piasecka, MagdalenaAvdeeva, VarvaraMarkt, RudolfAvdonin, Pavel V.Markuszewski, MichelAverin, Alexei D.Marlin, NathalieAverina, Elena B.Marminon, ChristelleAvi, ShpigelmanMarmiroli, MartaÁvila-Flores, AntoniaMarocco, AntonelloAviles, Francesc XavierMarote, AnaAvin, VijayMarpu, Sreekar BabuAviñó, AnnaMarques Da Silva, DorindaAvramescu, Sorin MariusMarques, Alexandra P.Avramopoulos, AggelosMarques, Ana ClaraAwate, SanketMarques, M. MatildeAwni, Rasha A.Marquez, EdgarAyala-Zavala, Jesus F.Marr, Andrew C.Azab, WalidMarra, AlbertoAzad, Mohammad A.Marra, FabrizioAzaizeh, HassanMarrazzo, AgostinoAzhdarinia, AliMarrazzo, PasqualeAziz, FaisalMarrocchi, AssuntaAzizi, AliMarrone, RafaelleAzofra, Luis MiguelMarrs, James A.Azov, VladMarrugo-Negrete, José LuisAzushima, KengoMarsche, GuntherAzzimonti, BarbaraMarshall, PaulAzzini, ElenaMarsillach, JuditBaars, JohanMarsol, AlexisBabaev, EugeneMarta, Plonska-BrzezinskaBabafemi, Adewumi JohnMartano, GiuseppeBabu, SiddharthMartella, GiuseppinaBaby, André RolimMartelli, FaustoBacariza, CarmenMartí, EvaBacciocchi, MicheleMarti, GuillaumeBąchor, RemigiuszMartí, SergioBackhouse, DavidMartin Lawson, AtaBäckvall, Jan ErlingMartin, Christopher B.Bácskay, IldikóMartin, FranciscoBączek, KatarzynaMartin, FranckBaczyńska, DagmaraMartin, IanBadea, IldikoMartin, James D.Badea, MihaelaMartin, Jan M. L.Badeka, AnastasiaMartín, MargaritaBadimon, Juan J.Martín, María ElenaBadino, PaolaMartin, OlivierBadur, MehmetMartin, RachelBae, Jung-WooMartin, RainerBae, Tae-HyunMartinek, Tamás A.Bae, Youn-SangMartínek, VáclavBaeckens, SimonMartinez Alonso, MartaBaek, Seung-AMartínez Ávila, Guillermo Cristian GuadalupeBaenas, NievesMartinez De Lizarrondo, SaraBaeza, Juan JoséMartinez Hernandez, EliasBagdžiūnas, GintautasMartínez López, José IsraelBagetta, GiacintoMartinez Martinez, VirginiaBagi, PéterMartínez Rodrigo, JavierBaglioni, MicheleMartínez Urreaga, JoaquínBagnoli, LuanaMartínez, AlbertoBago, Juli R.Martínez, AnaBagryanskaya, ElenaMartínez, ConstantinoBahal, RamanMartínez, CristinaBahrim, GabrielaMartínez, SidoniaBahuguna, AshutoshMartinez, VicenteBai, ChenxiMartínez-Cifuentes, MaximilianoBai, FangMartínez-Espinosa, Rosa MaríaBai, LeiMartínez-García, MarcosBai, RenrenMartínez-Gómez, PedroBaidoo, EdwardMartínez-Lillo, JoséBailly, ChristianMartinez-Mayorga, KarinaBailly, FabriceMartínez-Monteagudo, Sergio I.Bailly, Jean-DenisMartinez-Moreno, CarlosBaiocchi, ClaudioMartinez-Navarrete, GemaBaj, TomaszMartínez-Palou, RafaelBajales, NoeliaMartínez-Rodríguez, SergioBajda, MarekMartínez-Sánchez, NoeliaBajda, TomaszMartínez-Tomé, María JoséBajerová, PetraMartínez-Tong, Daniel E.Bajguz, AndrzejMartinez-Valladares, MariaBajusz, DávidMartinez-Villaluenga, CristinaBak, AndrzejMartín-Gil, JesusBakalkin, GeorgyMartinho, José Manuel GasparBaker, Brent A.Martini, DanielaBaker, PaulMartini, PetraBaker, RobertMartini, SilvanaBaki, GabriellaMartínková, LudmilaBakonyi, ImreMartín-Molina, AlbertoBakre, Abhijeet A.Martinotti, SimonaBalagurusamy, NagamaniMartín-Ramos, PabloBalan, PrabhuMartín-Rapún, RafaelBalanč, BojanaMartins, AliceBalaraman, VelmuruganMartins, IanBalashov, Victor N.Martins, José A.Balasubramanian, BalamuralikrishnanMartins, LuisaBalawejder, MaciejMartins, M. RosarioBaláž, MatejMartins, Mónia A. R.Balažová, AndreaMartins, NataliaBalazsi, CsabaMartins, RosárioBalcar, HynekMartins, SoniaBalcerczyk, AnetaMartins, Verónica C.Baldassarre, Maria ElisabettaMartins-Bessa, AnaBaldi, FrancoMarto, JoanaBaldini, MarioMartos, PerryBaldino, LuciaMartos, VanessaBaldisserotto, AnnaMartra, GianmarioBalestra, DarioMartucci, AnnalisaBalestri, FrancescoMartucciello, NadiaBalewski, ŁukaszMarty, Jean-DanielBálint, ErikaMartz, FrancoiseBallabio, DavideMaruca, AnnalisaBallandras-Colas, AllisonMarucci, GabriellaBallante, FlavioMaruyama, IchiroBallesté, Ruben MasMaruyama, RikaBallester, Pedro J.Marverti, GaetanoBallesteros, MentaMarzaro, GiovanniBallesteros-Garrido, RafaelMarzec, AnnaBalme, SébastienMarzo, LeyreBalmer, LoisMarzocco, StefaniaBalogh, KrisztiánMarzorati, StefaniaBalogh, LeventeMarzullo, Vincenzo ManuelBalogh, ZsoltMasaki, TakayukiBalslev, HenrikMasaoka, TatsuhiroBalzano, TizianoMascia, LenoBamba, TakeshiMasetti, AntonioBan, YusukeMashima, RyuichiBanach, MarcinMasi, MarcoBanach, MateuszMaslak, Veselin R.Banchelli, MartinaMasłyk, MaciejBanchero, MauroMason, NigelBanciu, ManuelaMaspoch, SantiagoBandapalli, Obul ReddyMassa, AntonioBandarenka, Hanna V.Massadi, Omar AlBandura, LidiaMassari, SerenaBandyopadhyay, DebasishMasselli, ClaudiaBandyopadhyay, SupriyoMassiani, PascaleBane, Susan L.Massimiliano, CordaroBanerjee, AbhinandanMassiot, GeorgesBanerjee, DebabrataMassotte, DominiqueBang, HyoweonMassoud, SalahBang, Marie-LouiseMastanjević, KristinaBankova, VassyaMasternak, JoannaBanning, AntjeMastinu, AndreaBannister, ThomasMastrogianni, OrthodoxiaBanoub, Joseph H.Mastrotto, FrancescaBansal, KuldeepMasu, HyumaBanskota, Arjun H.Masuda, HidekiBanti, Christina N.Masullo, MariorosarioBanyasz, AkosMata, RicardoBao, JinkuMatanović, IvanaBao, YongmingMatassa, SilvioBao, YongpingMatczak, PiotrBarakat, AssemMatczak-Jon, EwaBarałkiewicz, DanutaMateescu, Mircea AlexandruBarančík, MiroslavMatei, IoanaBaranyai, Edina FehérnéMateo, CesarBarao, Carlos E.Mateo, DiegoBaratto, LuciaMateo, ReyesBarbalexis, PanagiotisMaterazzi, StefanoBarbante, CarloMaterska, MałgorzataBarbaric, MonikaMatesanz, Ana I.Barbasiewicz, MichalMatesic, LidiaBarbera, JoaquinMateus, PedroBarbero, NadiaMathe, EwyBarbieri, FedericaMathew, KattathuBarbiero, IsabellaMathieu, DidierBarbon, SilviaMathieu, MarchivieBarboni, LucianoMathivathanan, LogeshBarbosa, ArmenioMatiadis, DimitrisBarbosa, MarciaMatichenkov, VladimirBarbosa, Raquel De MeloMatikonda, SiddharthBarbu, VasilicaMatkowski, AdamBarca, AmilcareMatos, InêsBarceló-Coblijn, GwendolynMatos, ManuelBardají, EduardMatosiuk, DariuszBardet, MichelMatouskova, PetraBardi, GiuseppeMátravölgyi, BélaBare, SimonMatsidik, RukiyaBarek, JiříMatsoukas, MinosBarjas, Gustavo CabreraMatsubara, KoukiBarker, DavidMatsuda, AkioBarker, JamesMatsuda, TomokoBarker, Philip J.Matsuda, YokoBarker, TylerMatsuda, YuBarki-Harrington, LizaMatsuda, YudaiBarkoulas, MichalisMatsugi, MasatoBarlocco, DanielaMatsugo, SeiichiBarlow, James W.Matsumoto, KazuyaBarnett, David A.Matsumoto, Ken’ichiroBarok, MárkMatsumoto, ShojiBaron, MarcoMatsumoto, TakayukiBaroutaji, AhmadMatsumoto, YasuhikoBarradas, Thaís NogueiraMatsunaga, KazuhisaBarraja, PaolaMatsunami, KatsuyoshiBarranco, AngelMatsuo, HiroshiBarreca, DavideMatsuo, TatsuhitoBarreca, SalvatoreMatsuo, YukikoBarreiro, Maria FilomenaMatsuura, BunzoBarreiro, Sonia LosadaMatsuura, KazunoriBarresi, AntonelloMatsuya, YujiBarreto, Maria CarmoMatta, CherifBarroca, Maria JoãoMattarelli, MaurizioBarron, Andrew R.Matthews, Michael A.Barros Gonçalves, Luciana RochaMatthews, PeterBarros, AnaMatthews, SusanBarros, Marcelo PaesMatthies, MichaelBarry, NicolasMattioli, Anna VittoriaBar-Shir, AmnonMattivi, FulvioBart, Bart LimburgMattoo, Autar K.Bartczak, PiotrMattos, CarlaBartel, KarinMatuszek, KarolinaBartella, LuciaMatwijczuk, ArkadiuszBarth, AndersMatyáš, RobertBartlett, JohnMatysiak, JoannaBartoli, MattiaMauk, Michael G.Bartolini, PaoloMaurer, AndreasBartoloni, Fernando H.Mauri, PierluigiBartosz, GrzegorzMaurício Alves, Da Motta SobrinhoBartoszak-Adamska, ElżbietaMauriz, ElbaBartoszek, AgnieszkaMaurizi, AntonioBartyzel, AgataMauro, MatteoBarvik, IvanMaury, OlivierBarwiolek, MagdalenaMautner, FranzBarygina, VictoriaMaveyraud, LaurentBaryshnikov, GlibMavri, JanezBasavegowda, NagarajMavrogonatou, EleniBascands, Jean-LoupMavromoustakos, ThomasBaschieri, AndreaMavumengwana, VuyoBasco, LeonardoMawatari, YasuteruBasile, AdrianaMay, AndrewBasile, TeodoraMayence, AnnieBasini, GiuseppinaMayer, DirkBasit, AbdulMayer, RupertBasith, ShaherinMayer, ThomasBaskaran, RathinasamyMayer-Gall, ThomasBaskin, IgorMayorga-Martinez, Carmen C.Basnett, PoojaMays, Jacqueline W.Bassani, AndreaMayumi, KoichiBassoli, AngelaMayyas, MohannadBassols, AnnaMazaira, GiselaBastarrachea, Luis J.Mazej, ZoranBastiat, GuillaumeMazerbourg, SabineBastida, AgathaMazin, AlexanderBaswan, SudhirMazivila, SarmentoBaszanowska, EmiliaMazumder, KishorBatey, Robert T.Mazur, AndrzejBatista, IrineuMazur-Bialy, AgnieszkaBatista, PatríciaMazzafera, PauloBatista, RamonMazzanti, MicheleBatista, RonanMazzoccanti, GiuliaBatta, GyulaMazzola, Eugene P.Battaglia, YuriMazzone, GloriaBattiato, AlfioMazzoni, RitaBaud, StephanieMba Blázquez, MiriamBaudis, StefanMbey, Jean AimeBauer, EikeMbita, ZukileBauer, RudolfMcCarthy, PumtiwittBaumann, ArndMcCartney, FionaBaumann, HeinzMcClure, JamesBaumann, MarcusMcComb, JenBaumli, PeterMcCormick, Laura J.Bauzá, AntonioMcDonald, PaulBavaro, Simona LuciaMcDougal, Owen M.Bavaro, TeodoraMcFeeters, RobertBay, Denice C.McGlinchey, Michael J.Bayer, PeterMcGrattan, Kevin B.Bayer, WibkeMcIntyre, Dustin L.Bayes-Garcia, LauraMcKay, Ryan T.Baykov, SergeyMckee, VickieBayona, JosepMcLemore, Gabrielle LynnBayse, CraigMcMillin, Kenneth W.Bazwinsky-Wutschke, IvonneMcMillin, MatthewBazylińska, UrszulaMcMorran, DavidBazylko, AgnieszkaMcNeil, DavidBazzicalupi, CarlaMcPhail, Kerry L.Beale, DavidMcPhillie, MartinBeauchemin, DianeMcreynolds, Katherine D.Bębenek, EwaMcSweeney, Matthew B.Beć, Krzysztof B.Md Badrul, AlamBechthold, AndreasMd, ShadabBeck, AndreasMeagher, RobertBeck, Tyler C.Meana Paneda, RubenBeck, William T.Means, Robert T.Becker, DanielaMeca, GiuseppeBecker, GuillaumeMeccariello, RosariaBecker, PierreMecinović, JasminBeckett, Michael A.Męczyńska-Wielgosz, SylwiaBeckingham, BryanMedana, ClaudioBedini, EmilianoMedarevic, DjordjeBednar, PetrMedeiros, Adriane Bianchi PedroniBednarek, RadoslawMeder, RogerBednárová, LucieMedici, SerenellaBednarski, Patrick J.Medina Juarez, Luis AngelBednarz-Knoll, NataliaMedina, Scott H.Bedoni, MarziaMedina-Franco, José L.Beemon, KarenMedina-González, YaocihuatlBeers, EricMedina-Ramirez, AdrianaBeghelli, DanielaMedio-Simón, MercedesBeghetto, ValentinaMedová, MichaelaBeharry, AndrewMedunic, GordanaBeharry, Kay D.Medved, IgorBehrends, VolkerMedvidović-Kosanović, MartinaBehringer, ErikMeegan, Mary J.Beidaghy Dizaji, HosseinMeerson, AriBeis, DimitrisMeeta, GeraBeitz, ToralfMegiel, ElżbietaBeklemishev, MikhailMehandzhiyski, AleksandarBel’skaya, LyudmilaMehariya, SanjeetBelBruno, JosephMehl, FlorenceBelcari, AntonioMehta, SunaliBelda-Palazón, BorjaMei, YangBelka, MariuszMeier-Menches, Samuel M.Bell, Thomas W.Meijide, FranciscoBellaloui, NacerMeiri, DavidBellelli, RobertoMeister, IsabelBellemin-Laponnaz, StéphaneMejía-Salazar, Jorge RicardoBellich, BarbaraMelcher, KarstenBellini, CostanzoMele, GiuseppeBello, ClaudiaMeléndez-Alafort, LauraBello, MartinianoMelendi-Espina, SoniaBelloncle, ChristopheMelguizo-Guijarro, ManuelBelloso-Uribe, KepaMelini, ValentinaBellucci, StefanoMelinte, VioletaBellumori, MariaMelko, JoshuaBelluti, FedericaMelkonyan, FerdinandBelmonte, Maria TeresaMellano, Maria GabriellaBelonenko, MikhailMelman, ArtemBelter, AgnieszkaMelo, AndréBeltrán Sanahuja, AnaMelo, Maria J.Beltran, María Del Carmen SánchezMelo, PedroBeltrán, SagrarioMelo, PriscillaBenaglia, MaurizioMelucci, DoraBenčić, ĐaniMemo, MaurizioBencini, AndreaMemon, TosifaBencsik, PeterMen, YongjunBencsik, TimeaMena Rejón, Gonzalo JoaquínBend, JackMenasché, PhilippeBende, AttilaMendel, MartaBender, DirkMendes, AdrianoBenedec, DanielaMendiola, JoséBenedek, GiorgioMendoza-Nuñez, Victor ManuelBenediktsdottir, BerglindMenegatti, StefanoBenesperi, IacopoMeneghello, RobertoBéni, SzabolcsMeneghetti, FiorellaBeninati, SimoneMenendez, EstherBenitez, SoniaMenezes, Helvécio CostaBenítez-Mateos, Ana I.Menezes, Irwin Rose AlencarBenito, AdriánMenezes, José C. J. M. D. S.Benito, Juan M.Menezes, PrashanthBennato, FrancescaMeng, DongBennis, NoureddineMeng, GeBenoit, DenizotMeng, QingshiBen-Salem, SalmaMeng, XiangchaoBenson, HeatherMeng, XiaoliBentel, Jacqueline M.Menger, Fredric M.Benvenuti, StefaniaMenghini, LuigiBenyhe, SandorMenichetti, StefanoBerardi, Anna ConcettaMenke, AndréBerdonosov, PeterMenkissoglu-Spiroudi, UraniaBerenjian, AydinMenna, MarialuisaBeretta, Giovanni L.Menon, Nishanth VenugopalBergamaschi, MatteoMensik, MiroslavBerger, Daniela CristinaMequanint, KibretBerger, Ralf G.Merah, OthmaneBergés Tiznado, Magdalena ElizabethMercader, JosepBerillis, PanagiotisMercandelli, PierluigiBerlin, JoshuaMercante, LuizaBerlin, K. DarrellMercuri, Nicola BiagioBerlowska, JoannaMereiter, StefanBerman, PaulaMergia, AyalewBermeshev, MaximMerighi, AdalbertoBermudez, MarcelMériguet, GuillaumeBerna, JoseMerino Peláez, GraciaBernacchi, SébastienMerino, GabrielBernal, VicenteMerino-Montiel, PenelopeBernard, Marek K.Merlino, AntonelloBernardes, NunoMernyák, ErzsébetBernardi, LucaMerola, Joseph S.Bernardo, UmbertoMertas, AnnaBernards, MatthewMerten, ChristianBernascon, LeonardoMeshram, ChetanBernat, PrzemysławMesquita, JoãoBernatchez, JeanMessa, PiergiorgioBernatová, SilvieMessinger, RobertBernède, Jean ChristianMessyasz, BeataBernhagen, JürgenMester, Patrick-JulianBerni, RobertoMestre, Ana S.Bernini, RobertaMethling, KarenBernstein, ElliotMewis, Ryan E.Bernstein, NiritMeyer, AndreasBerreau, Lisa M.Mezule, LindaBerrotéran-Infante, NeydherMezzetta, AndreaBerrue, FabriceMi, Fwu-LongBerski, WiktorMi, LanBerteina-Raboin, SabineMiao, LinglingBertelloni, FabrizioMiao, QingqingBerthold-Pluta, AnnaMicale, NicolaBerti, FedericoMiceli, AlessandroBertin, Matthew J.Miceli, NataliziaBertrand, MartineMichaelakis, AntoniosBertsch, ArnaudMichaelian, KaroBertsch, PascalMichaelis, MartinBertsouklis, KonstantinosMichail, ChristosBertuzzi, GiulioMichalak, AgnieszkaBertuzzi, TerenzioMichalik, JanBesalú, EmiliMichalska, AnnaBessmertnykh-Lemeune, AllaMichaud, GuillaumeBesson, ThierryMichaud, PhilippeBest, StephenMichaud-Soret, IsabelleBetancur, StefaníaMichel, Magdalena M.Betekhtin, AlexanderMichel, PiotrBetemariam, Senait AshenafiMichelacci, Yara M.Betoret, NoeliaMicheletti, GabrieleBettencourt, Ana FranciscaMichelle, PalmieriBettini, SimonaMichl, ThomasBettotti, PaoloMichna, AnetaBeyeh, Ngong KodiahMicic, MiodragBeyer, SebastianMicillo, RaffaellaBezirtzoglou, EugeniaMicucci, MatteoBhandari, Dhaka RamMidgley, AdamBharadwaj, ShivMieczkowski, AdamBharath, Leena P.Miecznikowski, JohnBhargava, AditiMiekisch, WolframBhaskar, AbhinavMiękus, NataliaBhat, OwaisMielańczyk, AnnaBhatt, PriyankaMielczarek, PrzemyslawBhattacharjee, Apurba KrishnaMierzejewska, JolantaBhattacharjee, SonaliMigaud, Marie E.Bhattacharya, AbhisekMigliori, Carmela AnnaBhattacharya, PratipMiguel, Ángel AguirreBhattacharya, RajatMiguel, Maria Da Graça CostaBhattacharya, SupriyoMihai, MarcelaBhatti, Muhammad MubashirMihajlović, IvanaBhosale, SheshanathMihasan, MariusBhowmik, Pradip K.Mija, AliceBhusal, RamMika, DelphineBiagi, MarcoMikac, Lara M.Bialecka-Florjańczyk, EwaMikata, YujiBiałek, AgnieszkaMikhelson, KonstantinBiałek, MałgorzataMiki, YasuhiroBian, HuiyangMikolajczak, PrzemyslawBian, YongzhongMikros, EmmanuelBianchi, AntonioMikstacka, RenataBianchi, MassimilianoMikulic-Petkovsek, MajaBianchini, FrancescaMikulski, DawidBianco, AndreaMikuš, PeterBianco, ArmandodorianoMilán-Carrillo, JorgeBianco, GiulianaMilanese, ChiaraBiancolillo, AlessandraMilanesio, MarcoBidarra, Sílvia J.Milardi, DaniloBideshi, DennisMilasius, RimvydasBidet, YannickMilazzo, Maria FrancescaBidwai, AshokMilczarek, MagdalenaBiedermann, DavidMilczarek, MałgorzataBiel, WiolettaMildren, RichBielas, RafałMilea, DemetrioBielejewski, MichalMilella, LuigiBielenica, AnnaMiletić, Goran I.Bieliński, Dariusz M.Miletín, MiroslavBiernasiuk, AnnaMilham, PaulBiesaga, MagdalegaMilia, EgleBiffis, AndreaMilic, NatasaBijak, MichałMilke, RalfBilal, MuhammadMilković, LidijaBilancio, AntonioMilledge, JohnBildstein, BennoMiller, JustinBílek, VlastimilMiller, ReinhardBilewicz, AleksanderMillet, MauriceBilia, Anna RitaMillet, ÓscarBilitewski, UrsulaMills, GermanBillard, ThierryMills, KenBilska-Wilkosz, AnnaMilojević-Rakić, MajaBin, XuMiloso, MariarosariaBinda, ElisaMilton, IñakiBini, RobertoMimica-Dukic, NedaBiniari, KaterinaMin, Kyeong-SikBinotto, GianniMinakshi, ManickamBiondi, MarcoMinami, AtsushiBiot, ChristopheMinami, IchiroBirch, BrianMinami, MasaakiBird, Phillip I.Minami, TsuyoshiBirke, Ronald L.Minami, YasunoriBirková, AnnaMinbiole, Kevin P. C.Birsa, Mihail LucianMincheva, RosicaBisbey, RyanMindt, ThomasBisceglie, FrancoMinehan, Thomas G.Bischof, JohannesMineva, TzonkaBishop, RogerMinguez, LaetitiaBisio, FrancescoMiniero, Daniela ValeriaBišová, KateřinaMinkiewicz, PiotrBisti, SilviaMinnaar, PhillipBis-Wencel, HannaMinnelli, CristinaBisz, ElwiraMino, LorenzoBiziuk, MarekMinocha, SubhashBiziuk, Marek K.Minofar, BabakBizzarri, Bruno MattiaMinorics, RenátaBizzarri, MarianoMinutolo, FilippoBizzo, HumbertoMinutti, CarlosBjerklin, KristerMiranda, ClaudioBjornstedt, MikaelMiranda, CristobalBlacha-Grzechnik, AgataMiranda, Jose M.Black, DavidMiranda, KatrinaBlackwell, BarbaraMiriam Maria, De ResendeBlagojevic Zagorac, GordanaMirkhani, Seyyed AlirezaBlakemore, James D.Mirković, MilicaBlalock, WilliamMironescu, MonicaBlamires, SeanMironova, MariaBlancaflort, LluísMiroslaw, BarbaraBlanco, AngelesMirza, Muhammad UsmanBlanco, EduardoMirzaeva, IrinaBlanco, Francisco J.Misaelides, PanagiotisBlanco-Fernandez, BarbaraMiseki, YugoBlandin, GaetanMiseta, AttilaBlando, FedericaMishima, MasakiBlanton, CynthiaMishra, Amit KumarBlasi, FrancescaMishra, Nagendra N.Blasiak, JanuszMishra, NigamBłaszczyk, JarosławMishra, Pawan KumarBlay, GonzaloMishra, RosalinBlay, VincentMishra, YogendraBlazevic, IvicaMishyna, MaryiaBłażewicz, AnnaMisicka, AleksandraBłażewska, Katarzyna M.Misra, Rajesh ChandraBlazquez, Ana-BelenMistrik, MartinBlázquez, EnriqueMisul, DanielaBlersch, David M.Mita, GiovanniBlind, RaymondMitaine-Offer, Anne-ClaireBlindauer, Claudia A.Mitakou, SofiaBloch, SylwiaMitani, ShoheiBloch, WitoldMitani, TakakazuBlom, BurgertMitchell, Angela M.Blonska, MarzennaMitchell, Cassie S.Blough, Bruce E.Mitchell, Scott G.Blumel, JanetMitchell, ToddBlythe, Linda L.Mitjans Arnal, MontserratBoanini, ElisaMitoraj, MariuszBobak, ŁukaszMitran, GheorghiţaBobek, JanMitran, Raul-AugustinBober-Majnusz, KatarzynaMitsou, Evdokia K.Bobis, OtiliaMitsudo, KoichiBobrovsky, AlexeyMitulovic, GoranBocarsly, MiriamMiyagawa, HayatoBochkarev, M. N.Miyamae, YusakuBoddaert, ThomasMiyamoto, KojiBode, Bela ErnestMiyanaga, AkimasaBodea, Liviu-GabrielMiyata, YoshikiBodenstine, ThomasMiyoshi, DaisukeBodoki, EdeMiyoshi, NorioBoege, FritzMizerska, UrszulaBoeglin, AlexMizerska-Kowalska, MagdalenaBoehr, David D.Mizobata, TomohiroBoekema, BoukeMizoshiri, MizueBoese, Adrian DanielMizumoto, ShujiBoffo, Elisangela F.Mizuno, Cassia SBoga, CarlaMladěnka, PřemyslBogdan, CatalinaMladenović, MilanBogdanchikova, NinaMlcek, JiriBogdanov, Alexey A.Mleczek, MiroslawBogdański, PawełMlejnek, PetrBoggia, RaffaellaMlostoń, GrzegorzBoggioni, LauraMnif, WissemBogliotti, NicolasMoad, GraemeBognár, GabriellaMobbili, GiovannaBogush, AnnaMoccia, StefaniaBogusławska, JoannaMochizuki, HitoshiBohinc, KlemenMochizuki, ShinichiBöhm, VolkerMočibob, MarkoBohman, BjörnMock, BeverlyBoichuk, SergeiModec, BarbaraBoika, AliakseiModenutti, CarlosBojarová, PavlaModica, MariaBojarska, JoannaModolo, Luzia ValentinaBojić, MirzaModugno, FrancescaBojková, BiankaModukuri, RamkumarBokach, NadezhdaMoein, Mohammad MahdiBoland, MikeMoghtadernejad, SaraBolanos-Garcia, Victor M.Mogielnicki, AndrzejBölcskei, HedvigMohamed, Azza H.Bölcskei, KataMohamed, SharmarkeBoldizsár, FerencMohamed, TamerBolibruchová, DanaMohammed, AltafBolokang, Amogelang SylvesterMohammed, SohaibBolonio, DavidMohankumar, KumaravelBolotin, Dmitrii S.Mohanty, Joy G.Bonacina, FabriziaMohles, VolkerBonadies, IreneMoita, Ana SofiaBonamore, AlessandraMojzych, MariuszBonardd, SebastianMok, Daniel Kam-WahBonasera, AurelioMokshin, Anatolii V.Bonaventura, GiovanniMoldogazieva, Nurbubu T.Bondon, ArnaudMoldovan, MirelaBondzior, BartoszMoldovan, Radu-CristianBonferoni, Maria CristinaMoldoveanu, Serban C.Bongarzone, ItaliaMolina Salinas, Gloria MaríaBonikowski, RadosławMolina-Cerrillo, JavierBonin, SerenaMolina-Espeja, PatriciaBonnet, Célia S.Molina-Santiago, CarlosBonnet, PascalMolinie, RolandBonnet, Pierre-AntoineMoll, Henri C.Bonofiglio, DanielaMollace, VincenzoBonomo, MatteoMollica, AdrianoBonrath, WernerMollica, GiuliaBontempo, PaolaMolognoni, LucianoBonucci, AlessioMołoń, MateuszBonyár, AttilaMoloney, Mark G.Boo, Yong ChoolMolteni, GiorgioBooth, BrianMomekov, GregoriBoratyński, PrzemysłąwMomekova, Denitsa B.Borbás, AnikóMonai, MatteoBorbas, K. EszterMonari, AntonioBorcan, FlorinMoncalián, GabrielBordignon, EnricaMondal, KunalBorek, WojciechMondal, MukuleshBorges, AlissonMondanelli, GiadaBorges, RicardoMonge, DavidBorges, WarleyMoni, LisaBorghi, MonicaMonica-Maria, BaiaBorgonovo, GigliolaMonika, Waksmundzka-HajnosBoris, PejinMoniuszko-Szajwaj, BarbaraBork, KayaMoniz, TâniaBorka, DuškoMonnerat, GustavoBorkowska, Edyta MartaMonroe, Mary Beth BrowningBorole, AbhijeetMonsalve, MariaBoroń, DariuszMontagne, KevinBoros, DanutaMontagnon, TamsynBoros, LászlóMontalvo González, EfigeniaBorota, AnaMontalvo-Rodríguez, RafaelBorowiec, JoannaMontanez, Julio CesarBorowik, AgataMontaño, Gabriel A.Borse, VikrantMontarolo, FrancescaBorska, SylwiaMontecchio, DanieleBortolozzi, RobertaMontecucco, FabrizioBortoluzzi, MarcoMonteiro, CarlosBorys, KrzysztofMonteiro-Neto, ValérioBoryski, JerzyMontenarh, MathiasBorza, TudorMontenegro, IvánBosch, JoanMontero, OlimpioBoschi-Muller, SandrineMontes Morán, Miguel A.Boschin, GiovannaMontesano, CamillaBoscoboinik, Jorge AnibalMontesano, DomenicoBoselli, EmanueleMontesarchio, DanielaBosetti, MichelaMontes-García, VerónicaBoskovic, ZarkoMonti, DanielaBosnar, Maja HerakMonti, MarcoBosso, AndreaMontiel-Smith, SaraBoström, Kristina BengtssonMontoliu-Gaya, LaiaBotana, Ana MaríaMontone, Carmela MariaBotelho, JoãoMontoro, PaolaBotez, ElisabetaMontouillout, ValérieBotha, FrederikMontoya-Castillo, AndrésBotondi, RinaldoMonts, DavidBotoran, OanaMoon, Geon DaeBotta, BrunoMoon, Il SooBottari, FabioMoon, Jong-SeokBotubol-Ares, José ManuelMoon, MinhoBou, RicardMoon, YuseokBoucekkine, AbdouMoore, StuartBoucheix, ClaudeMoore, TaraBoucher, DaveMoosmann, BerndBoucher, David S.Mor, AmramBouckaert, JulieMorabito, RossanaBoudaden, JamilaMoraca, FedericaBoudon, JulienMoraga, JavierBougrin, KhalidMorais, MauricioBouillaud, FredericMorais, Tânia S.Boukouvalas, JohnMorak-Młodawska, BeataBourdon, EmmanuelMorales Martín, AnaBourgoin, SylvainMorales Suárez-Varela, María M.Bourneuf, EmmanuelleMorales, JavierBouropoulos, NikolaosMorales, Jose ManuelBourson, PatriceMorales, PatriciaBouvier, BenjaminMorales, PaulaBovi Karatay, Graziele GrossiMorales-de La Peña, MarianaBowden, BruceMorales-González, José AntonioBowman-James, KristinMorales-Morales, DavidBoyle, MariaMoran, WesleyBoysen, ReinhardMoratti, Stephen CBozdag, MuratMoravcová, DanaBozio, RenatoMorbidelli, LuciaBozzo, GiancarloMorcillo, Miguel ÁngelBrabet, PhilippeMordalski, StefanBracher, FranzMoreira Gonçalves, LuísBradley, MarkMoreira, DanieleBradshaw, BenMoreira, Ibério De P. R.Bradshaw, PatrickMoreira, Irina S.Braendle, ChristianMoreira, Vânia M.Braga, Anna Rafaela CavalcanteMorel, BertrandBraga, Maria-HelenaMorel, MauricioBraga, Susana SantosMoreno, AbelBraicu, CorneliaMoreno, AdrianBraig, SimoneMoreno, Antonio D.Bramanti, EmiliaMoreno, Diego A.Branca, FerdinandoMoreno, EstherBranca, Jacopo Junio ValerioMoreno, JordiBranca, Rosa TamaraMoreno, Juan J. Brancart, JoostMoreno, Maria JoãoBrancato, VirginiaMoreno, MiguelBrandt, CurtisMoreno-Piraján, Juan CarlosBrás, NatérciaMoreno-Rojas, José ManuelBrasic, JamesMoretto, AlessandroBraslavsky, Silvia E.Morgan, DavidBrassat, KatharinaMorgan, KevinBratu, AnaMorganti, PierfrancescoBraun, Andrew P.Morgunov, IgorBraunstein, PierreMori, MattiaBravo, LidiaMori, SeijiBravo, Susana B.Mori, ShigekiBrayden, David J.Mori, WasukeBrčić Karačonji, IrenaMorianos, IoannisBrea, OrianaMorigi, RitaBrebu, MihaiMorikawa, ToshioBrecker, LotharMorillas, LeandroBreeze, AlexMorimoto, YumaBregadze, VladimirMorimura, ShigeruBrégier, FrédériqueMorini, LucaBrehm, Maria AlexandraMorisseau, ChristopheBrehm, MartinMorita, NaokiBreidbach, AndreasMorita, NobuyoshiBreinbauer, RolfMoriuchi-Kawakami, TakayoBrela, MateuszMoriwaki, HiroshiBren, UrbanMoriyama, KatsuhikoBrender, JeffreyMorjani, HamidBresciani, ElenaMorkunas, IwonaBrestic, MarianMormann, MichaelBreton, Gary W.Mornar, AnaBreugst, MartinMorocho, VladimirBreza, MartinMorozov, Vitaly A.Brezesinski, TorstenMorrison, JulietBrezovský, JanMorrone, AldoBrichacek, MatthewMorselli, DavideBriedis, VitalisMortara, LorenzoBriffa, SophieMortier, JérémieBriganti, MatteoMorzelle, Maressa CaldeiraBrígido, ClarisseMorzycki, Jacek W.Brignole, ChiaraMosca, LucianaBrindisi, MatteoMoschini, RobertaBrindley, DavidMoschopoulou, EkateriniBritton, JonathanMoschopoulou, GeorgiaBriz, OscarMosharov, Eugene V.Broadhurst, Catherine LeighMoskaleva, LyudmilaBrocławik, EwaMosnáček, JaroslavBroda, Małgorzata A.Mosqueira, DiogoBrodosi, LuciaMosqueira, MatiasBrogi, SimoneMosquera, MartaBroniatowski, MarcinMossine, Andrew V.Bronisz, RobertMossine, Valeri V.Bronowska, AgnieszkaMostafavi, MehranBrook, DavidMot, AugustinBrooks, AllenMota, Antonio J.Brooks, Benjamin D.Mota-Morales, Josué D.Brotin, ThierryMotoyama, KeiichiBrouwers, Jos F.Motrescu, IulianaBrouzgou, AngelikiMou, YongchaoBrovarets, Volodymyr S.Mouahid, AdilBrown, Alan B.Mouga, TeresaBrown, DennisMoujir, Laila M.Brown, Janet E.Moura, Nuno M. M.Brown, LindsayMoura, RuteBrown, TomMourão, Paulo A. S.Brozovic, AnamariaMourtzinos, IoannisBruce, JamesMousley, CarlBruckner, ChristianMoussaoui, YounesBruder-Nascimento, Thiago DoMoustakas, MichaelBrugarolas, PedroMoyano, AlbertBruijn, Johannes DeMoyano-Mendez, Jose RamonBrulíková, LucieMoya-Ramírez, IgnacioBrullo, ChiaraMoyseowicz, AdamBrune, WolframMozaffari, SaeedBrunel, FabriceMozejko-Ciesielska, JustynaBrunetti, LuigiMozgawa, WłodzimierzBrünnert, DanielaMozhzhukhina, NataliiaBruno, MattosMozo, Juan DanielBruno, VictorMozumder, Md. Salatul IslamBruns, Carson J.Mphahlele, Malose JackBrunschweiger, AndreasMravec, JozefBrüssow, HaraldMráz, JaroslavBrust, TarsisMrówczyński, RadosławBrust-Mascher, IngridMrowiec-Białoń, JulitaBrutti, SergioMróz, ZenonBryant, Matthew S.Mshvildadze, VakhtangBrycki, BogumilMu, QingxinBryjak, MarekMuccilli, VeraBryk, PawełMuceniece, RutaBryla, DavidMucha, IgorBrys, JoannaMuchowicz, AngelikaBrzeska, JoannaMuchowska, Kamila B.Brzeszcz, JoannaMuders, MichaelBrzezinski, MarekMueller, Paul A.Brzozowski, TomaszMueller, Thomas J.J.Bu, PengliMughal, AmreenBubnov, AlexeyMuhle-Goll, ClaudiaBuchberger, WolfgangMühlenhoff, UlrichBuchet, ReneMujtaba, MuhammadBuchowicz, WłodzimierzMukai, HidehitoBuchtová, HanaMukai, RieBuckley, M.Mukhametzyanov, Timur A.Bucur, BogdanMukherjee, ShayantiBudryn, GrażynaMulak, AgataBudyka, Mikhail F.Mulas, MaurizioBudzak, SimonMulcahy, BenBudzevich, MikalaiMulherkar, ShalakaBudzevich, Mikalai M.Mullen, JackBudzianowski, JaromirMüller, CarstenBudzisz, ElżbietaMuller, ChristianBueno-Silva, BrunoMüller, ChristophBugarin, AlejandroMuller, EricBugni, TimMüller, GerhardBuha, AleksandraMüller, JoachimBuica, AstridMüller, JulianeBuijs, WimMüller, KajetanBujak, MaciejMüller, KarstenBujak, TomaszMuller, Patricia A. J.Bujanovic, BiljanaMüller, ThomasBujnicki, BogdanMüller, Thomas ErnstBukowski, MichalMüller, UweBulánek, RomanMüller, Werner E. G.Bulatov, EmilMüller-Vahl, Kirsten R.Bulboacă, Adriana ElenaMulmudi, Hemant KumarBulet, PhilippeMunekata, Paulo E.S.Bulgariu, LauraMuñoz, AsunciónBülow, Margret H.Muñoz, EduardoBulushev, DmitriMuñoz, Marcelo AndrésBuma, Wybren J.Muñoz-Castro, AlvaroBun Ng, TziMuñoz-Mingarro, DoloresBunce, Richard A.Muntean, DaninaBunea, AndreaMuntean, DeliaBungau, SimonaMunteanu, Florentina-DanielaBungau, Simona GabrielaMuntimadugu, EameemaBuniowska, MagdalenaMunusamy, ElangoBuonocore, FrancescoMura, UmbertoBura-Nakić, ElviraMurab, SumitBuranelo Egea, MarianaMurakami, KazumaBurcul, FrankoMurakami, YasufumiBurdak-Rothkamm, SusanneMuramatsu, AtsushiBureau, RonanMuranaka, AtsuyaBures, FilipMurase, DaikiBurge, KathrynMurata, ToshihiroBurgos, RafaelMuratani, MasafumiBurgos-Ramos, EmmaMuratova, AnnaBurke, AnthonyMuravyev, Nikita V.Burket, JessicaMurdan, SudaxBurkin, MaximMuresan, LianaBurley, Jonathan C.Muresan, VladBurns, Jonathan R.Murias, MarekBurns, Jorge S.Murillo, WalterBurpo, FredMurkovic, MichaelBurrai, Giovanni PietroMurphy, Caroline S.Burrola-Aguilar, CristinaMurray, Jane S.Bursic, VojislavaMurray, KeithBurton, Zachary F.Murtinho, DinaBury, WojciechMurugan, N. ArulBusardò, Francesco PaoloMuscara, Marcelo N.Busby, ChrisMusco, GiovannaBusco, GiovanniMusgrave, IanBusinaro, RitaMusiani, FrancescoBusquets, Maria AntòniaMusilek, KamilBusto, NataliaMusioł, RobertBustos, Diego MartinMusk, BillBuszewski, BogusławMusso, IoanaButenschön, HolgerMust, IndrekButler, Andrew A.Musumeci, ChiaraButler, JohnMusumeci, DomenicaBuzuk, MarijoMus-Veteau, IsabelleBuzzá, Hilde HarbMuszyńska, BożenaBydlowski, Sérgio PauloMuthukumar, KannanBylund, DanMuthuramu, IlayarajaByrdin, MartinMutiga, Samuel K.Byrne, HughMutlu, Ayse SenaBystrom, JonasMuto, KeiByun, SanguineMuxel, Sandra MarciaCaballero, AntonioMuzafarov, AzizCaballero, DanielMuziol, TadeuszCaballero-Casero, NoeliaMuziol, Tadeusz MikolajCaballero-Garcia, BeatrizMwanza, MulundaCabello-Garcia, TomásMyers, Timothy G.Cabeza Gras, ÓscarMykhaylyk, Oleksandr O.Cabral, HoracioMyllymäki, SamiCabrales, LuisMyong Jae, YooCabrele, ChiaraMyongwon Lee, LuciaCabrera, HumbertoMysliveček, JaromírCabrera-Chávez, FranciscoMysliwiec, TamiCabrera-Fuentes, Hector A.Na, DokyunCaccia, MarioNa, KunCacciola, FrancescoNabae, YutaCáceres, AlejandroNadǎş, George CosminCachada, Anabela Ferreira De OliveiraNadiminty, NagalakshmiCacini, SoniaNagaoka, ShojiCaddeo, CarlaNagarajan, BalajiCadiou, CyrilNagata, NaotoCádiz-Gurrea, María De La LuzNagatoishi, SatoruCafforio, PaolaNagatsu, AkitoCafiero, MauricioNagatsugi, FumiCaggia, SilviaNagel, RaimundCagliero, Cecilia LuciaNagel, StefanCahlíková, LucieNagulapalli Venkata, KalyanCai, BishuangNagy, CorinaCai, DeminNagy, Csaba LeventeCai, LishengNagy, EndreCai, WeiminNagy, LászlóCai, WenlongNagy, MiklósCai, ZhengxuNagy, TamásCaille, FabienNagy, VeronikaCailliau, KatiaNahm, Kee SukCailloux, JonathanNahra, FadyCaires, Hugo R.Naini, Santhosh ReddyCairns, Warren Raymond LeeNair, Anroop B.Cairrão, ElisaNair, Sandeep SudhakaranČakić Semenčić, MojcaNair, SreenathCala, AntonioNaito, ToshioCalabrese, LuigiNajda, AgnieszkaCalabretta, Maria MaddalenaNajeeb, ShariqCalabrò, Paolo S.Najlah, MohammadCalapai, GioacchinoNajmanová, IvetaCalasso, MariaNakabayashi, KojiCalatayud, Paul-andréNakabayashi, SeiichiroCalcabrini, CinziaNakabayashi, TakakazuCalcaterra, AndreaNakagaito, Antonio NorioCalcinotto, AriannaNakagawa, AtsushiCalcutt, MichaelNakagawa, HiroshiCalderisi, MarcoNakagawa, TetsuyaCalderón De La Barca, Ana MaríaNakagawa, YoshinaoCaleb, Oluwafemi JamesNakagawa-Goto, KyokoCalhelha, Ricardo CostaNakamaru-Ogiso, EikoCalì, TitoNakamura, IsseiCaliceti, CristianaNakamura, MitsutakaCalifano, ValeriaNakamura, SoichiroCaligiuri, IsabellaNakano, HirotoCalignano, AntonioNakano, ShogoCalina, DanielaNakano, Shu-ichiCalinescu, IoanNakano, TamakiCalín-Sánchez, ÁngelNakashima, KenichiCalissendorff, JanNakashima, SouichiCallery, Patrick S.Nakata, NorioCalligari, PaoloNakata, SatoshiCallone, EmanuelaNakayama, MasayoshiCaloca, Jose MariaNakayama, MizuhoCalogeropoulou, TheodoraNakayama, YuushouCalokerinos, Antony C.Nakazato, GersonCalpena, Ana C.Nakhjavani, MaryamCaltagirone, ClaudiaNalewajko-Sieliwoniuk, EdytaCalucci, LuciaNam, Il-WooCalvano, Cosima D.Nam, InhoCalvano, Cosima DamianaNam, Ki HyunCalvo, ConceptiónNam, Tae GyuCalvo, LourdesNamasivayam, VigneshwaranCalvo, Marta M.Namgaladze, DmitryCalzaferri, GionNamihira, MasakazuCamacho, M. EncarnaciónNanda, SatyabrataCamacho-Corona, María Del RayoNanduri, RavikanthCamaiti, MaraNandy, ArpitaCamara, Jose SousaNanno, MasanobuCamarasa, María-JoséNano, AdelaCamba, A. TundidorNanoff, ChristianCamele, IppolitoNantasenamat, ChaninCameron, DonaldNapoli, AnnaCameron, SimonNapoli, Edoardo MarcoCaminade, Anne-MarieNapolitano, AlessandraCamins, AntoniNarayan, MaheshCamire, MaryNardecchia, StefaniaCampagne, Jean-MarcNardi, MonicaCampana, RaffaellaNardi, SerenellaCampanella, AlessandroNardin, TizianaCampanella, BeatriceNarita, Shin-ichiroCampbell, BradleyNarkar, AmeyaCampbell, ColinNaryzhny, StanislavCampbell, Grant R.Nasarre, PatrickCampbell, KrisNascimento, Luís Adriano Santos DoCampbell, Phil G.Nascimento, YuriCampiglia, PietroNash, RobertCampilho, AnaNassar, Zeyad D.Campillo, NataliaNastasa, CristinaCampo, GiuseppeNasti, RitaCampo, RiccardoNastuta, Andrei VasileCampone, LucaNatale, PaoloCampos Rosa, Joaquín MaríaNatali Sora, IsabellaCampos, Alex Fabiano CortezNatali, FrancescaCampos, MariaNatarajan, ArutselvanCampos, Rafael KroonNath, ArijitCampos-Martin, Jose M.Nativi, CristinaCampos-Martínez, JoséNaumova, Anna V.Camps, IhosvanyNaumovski, NenadCanas, SaraNaumowicz, MonikaCancemi, PatriziaNaushad, MCandeias, Nuno R.Navarra, MicheleCandiani, GabrieleNavarrete-Vazquez, GabrielCanela, NúriaNavarro, ElenaCanela-Garayoa, RamonNavarro, ElisaCaneschi, AndreaNavarro, RodrigoCanetta, ElisabettaNavarro-Hoyos, MirthaCanfield, ScottNavickiene, SandroCanini, AntonellaNaviglio, DanieleCannistraro, SalvatoreNawaz, MuhammadCannon, RichardNawirska-Olszańska, AgnieszkaCanonico, BarbaraNawrot, BarbaraCantamessa, SimoneNawrot-Hadzik, IzabelaCantarelli, MiguelNayak, TapanCantini, FrancescaNayak, Usha Y.Canuti, ValentinaNazarchuk, EvgenyCao, DeliangNazarkina, ZhannaCao, JianNazarski, Ryszard B.Cao, JianyunNazemi, AliCao, XudongNazir, RashidCao, YongsongNaznin, Most TaheraCapasso, FrancescoNazzaro, FilomenaCapasso, IlariaNdika, JosephCapasso, RaffaeleNebot, CarolinaCapillo, GioeleNebra, NoelCapogni, MarcoNeculita, Carmen MihaelaCaponio, FrancescoNeda, IonCapozzi, VittorioNedialkov, Paraskev T.Cappellacci, LoredanaNees, MatthiasCapperucci, AntonellaNegahdar, LeilaCappiello, MarioNeginskaya, Maria A.Capriati, VitoNegrea, AdinaCapuano, FedericoNegrea, PetruCapurso, GiovanniNegri, GiuseppinaCaputo, FannyNegroni, LucCaputo, GregoryNehela, YasserCaputo, PaolinoNeilson, AndrewCaraballo, MauricioNeira, José L.Carabelli, ValentinaNeiva, Duarte M.Carabineiro, SoniaNelson, AndrewCarac, GetaNelson, RandyCarafa, MariaNelson, Richard W.Carata, ElisabettaNelyubina, YuliaCarazo, AlejandroNemec, IvanCarballeira, Nèstor M.Németh, RóbertCarbonaro, Carlo MariaNemoto, KiyomitsuCarbone, AlessandraNemykin, VictorCarcadea, ElenaNenadis, NiknenCarcelli, MauroNenajdenko, Valentine G.Carda, MiguelNeng, NunoCardador-Martínez, AnabertaNeo, Ana G.Cardellicchio, CosimoNeogi, UjjwalCardenas Zuniga, RobertoNeophytou, Christiana M.Cardia, Maria CristinaNesmelova, IrinaCardona, FrancescaNesterov, DmytroCardoso, SusanaNesterova, Oksana V.Cardoso, Susana M.Nestler, UlfCarfora, AnnaNeubauer, HeidiCarignani, ElisaNeubert, ReinhardCarina, ValeriaNeugebauer, DorotaCariou, KevinNeugebauer, WitoldCarla Aragoni, MariaNeunert, GrazynaCarlberg, CarstenNeureiter, DanielCarlier, PaulNeuwald, Daniel AlexandreCarlini, Célia R.Nevares, IgnacioCarlino, ElvioNevárez-Moorillón, Guadalupe VirginiaCarlos Salay, LuizNeves, BrunoCarlotto, SilviaNeves, JoãoCarmen, MejíaNeves, Maria Da Graça P. M. S.Carmona, Estefanía NúñezNeves, VeraCarmona-Ribeiro, Ana M.Newberg, John T.Carnevale Neto, FaustoNeyerlin, Kenneth CharlesCarney, RandyNg, AndyCarniato, FabioNg, CarolineCaroleo, Maria CristinaNg, Ho LeungCaronni, SarahNg, Tzi BunCarosio, FedericoNgai, ToCarpanese, DeboraNge, Thi ThiCarpentieri, AndreaNgezahayo, AnacletCarpes, Solange TeresinhaNguyen, Khoi TanCarqueijeiro, InêsNguyen, Minh ThoCarr, Carolyn A.Nguyen, Ngoc A.Carradori, SimoneNguyen, Nhat TruongCarrasco, HéctorNguyen, Thanh BinhCarrasco, SergioNguyen, TrieuCarrasco-López, CésarNguyen, Van KhanhCarré, VincentNguyen, WilliamCarregal-Romero, SusanaNi, BukuoCarrella, LucaNi, JunjunCarrera Fernández, CeferinoNiaura, GediminasCarrera, MónicaNicasio, Maria CarmenCarrero, Jose AntonioNicholas, DarrelCarretto, EdoardoNichols, Nancy N.Carriero, Maria VincenzaNiclos Gutiérrez, JuanCarrillo, CeliaNicolas, CyrilCarrillo, WilmanNicolau, ElodieCarrión, MarNicoletta, Fiore PasqualeCarrubba, AlessandraNicoletti, FerdinandoCarter, KatharineNicoletti, RosarioCarter, WayneNicoletti, VincenzoCarullo, GabrieleNicolotti, OrazioCaruntu, ConstantinNiculau, Edenilson Dos SantosCaruso, CalogeroNiculescu, LoredanCaruso, EnricoNie, ChuanxiongCaruso, GiuseppeNie, Guo-XingCaruso, UgoNie, LimingCarvajal-Millan, ElizabethNiedziela, TomaszCarvalheiro, Manuela CollaNiedzielski, PrzemysławCarvalho Pimenta, DanielNiedźwiedzka-Rystwej, PaulinaCarvalho, DanielNielsen, Peter EigilCarvalho, Luisa M.H.Nielsen, Vance G.Casadio, RitaNiemczak, MichalCasado-Coterillo, ClaraNiemczyk, AgataCasado-Martinez, M. CarmenNieminen, PenttiCasampai, AntalNiemirowicz-Laskowska, KatarzynaCasanova, LlanosNieoczym, DorotaCasasnovas, RodrigoNieto, GemaCasciaro, BrunoNieto, ManuelCascone, SaraNieto, Marcelo J.Case, RaymundoNievergelt, Adrian PascalCásedas, GuillermoNikitin, KirillCasey, JenniferNikolaev, EugeneCasiello, MicheleNikolaev, ViacheslavCasiraghi, AntonellaNikolaevskii, Stanislav A.Casnati, AlessandroNikolaidis, NikolaosCasoni, DorinaNikolaivits, EfstratiosCassidy, John F.Nikolakaki, EleniCastagna, MichelaNikolakakis, IoannisCastaldo, RacheleNikolakopoulou, Angeliki M.Castañeda-Corral, GabrielaNikolay V., SidorovCastan-Laurell, IsabelleNikolić, DraganCastellano, CarloNikoloudakis, NikolaosCastellari, MassimoNikolova, RositcaCastelli, Ivano E.Nima, Zeid A.Castelli, M. PaolaNinfali, PaolinoCastellini, ElenaNing, XuhuiCastellón, Enrique RodríguezNinomiya, SatoshiCastelo-Branco, MiguelNishi, KosukeCastiglione, FrancaNishi, MayumiCastilho, PaulaNishida, RitzuoCastillo, AntonioNishihara, YasushiCastillo, FranciscaNishikimi, AkihikoCastillo, JuanNishimoto, YoshioCastillo, SandraNishinaga, TohruCastñeiras, AlfonsoNishiwaki, HisashiCastoldi, LidiaNishiwaki, NagatoshiCastorena-Torres, FabiolaNishiyama, YusukeCastro, EulogioNissen, LorenzoCastro, José Javier FernándezNissinen, MaijaCastro, Mariana S.Nisticò, RobertoCastro, RemediosNistor, Ileana DenisaCastro, Ricardo A.E.Nita, Loredana E.Castro-Vargas, Henry I.Nitiss, JohnCastrucci, Ana MariaNitulescu, George MihaiCataldi, TommasoNitulescu, MihaiCataldo, SalvatoreNitz, MarkCatani, MartinaNitzsche, BiancaCatanzaro, GiuseppinaNiu, RanCatapano, CarloNiu, TianhuaCatar, RusanNixon, GemmaCatarino, IsabelNizetic, SandroCatarino, Marcelo D.Njar, Vincent C. O.Catarino, SusanaNkukwana, ThobelaCatauro, MichelinaNoble, BenjaminCaterino, MariannaNobre, LuísCattoën, XavierNocentini, SaraCatucci, GianlucaNoda, TakahiroCatucci, LuciaNoei, HeshmatCatuogno, SilviaNoël, SébastienCauchy, ThomasNogales-Bueno, JulioCaucia, FrancaNogami, MakotoCauda, ValentinaNogara, PabloCautela, DomenicoNogawa, ToshihikoCauteruccio, SilviaNoge, KojiCava, ClaudiaNoguchi, HiroshiCavalcanti, João Henrique F.Nogueira Perez, Juan JoseCavaleiro, José A. S.Noguera Artiaga, LuisCavallaro, GiuseppeNoguera, RosaCavallo, CarmenNogues, IsabelCavalluzzi, Maria MaddalenaNomikos, TzortzisCavani, LucianoNomngongo, Philiswa NosizoCavazzini, AlbertoNomura, MikihiroCavé, ChristianNomura, SeitaroCavicchi, KevinNora-Krukle, ZaigaCaviglia, Gian PaoloNorman, TrevorCaydamli, YavuzNorton, MichaelCazacu, MariaNose, TakeruCazzola, RobertaNoseda, Miguel D.Cebecauer, MarekNothias, Louis-FélixCeccarelli, GabrieleNoureddine, AchrafCeccato-Antonini, Sandra ReginaNovak Kujundžić, RenataCecilia, Juan AntonioNovak, PavelCecioni, SamyNovič, MarjanaCefalas, Alciviadis-ConstantinosNovikov, Alexander S.Celakovska, JarmilaNovinec, MarkoCelano, RitaNovio, FernandoCeleiro, MaríaNovío, SilviaCeletti, AngelaNovotna, VladimiraCella, EleonoraNový, PavelCellamare, SaverioNowacka-Jechalke, NataliaCembrowska-Lech, DanutaNowaczyk, JacekCena, CíceroNowak, AdrianaČėnas, NarimantasNowak, AgnieszkaCendrowski, AndrzejNowak, KrzysztofCepeda, JavierNowak, PawelCerazy-Waliszewska, JoannaNowakowska, JustynaCerbo, Alessandro DiNowicka, GrażynaCerezo, JavierNowicki, JanuszČernáková, LuciaNozaki, MasamiČerný, JiříNsanzabana, ChristianCerny, MiroslavNtaikou, IoannaCerón-Carrasco, José PedroNtalii, NikolettaCerra, BrunoNtalli, Nikoletta G.Cerrato, Giuseppina PinucciaNtie-Kang, FideleCerri, GuidoNumez, EstrellaCerri, Marcel OtavioNunes, AlexandraCerrito, Maria GraziaNunes, FernandoCerrone, FedericoNunes, LeonelCerruti, PierfrancescoNuñez, María B.Cerveny, SilvinaNunez, OscarCesar, VincentNuñez, RosarioCesarino, IgorNunzio, VicarioCesaro, AttilioNurieva, EvgeniyaCescutti, PaolaNussaume, LaurentČesonienė, LaimaNuţǎ, Diana CameliaCeulemans, ArnoutNuttall, Patricia A.Cézard, ChristineNwaiwu, OgueriChabaud, LaurentNycz, JacekChacko, Jenu VargheseNycz, Jacek E.Chae, MichaelNyman, Tuula AnneliChai, Kyu YunO’Duill, Miriam L.Chai, Tsun-ThaiO’Hare, TimChaimbault, PatrickOancea, FlorinChakrabarti, SubhadeepObata, TohruChakraborty, AnutoshObata, YasukoChakraborty, AtanuObaya, Alvaro J.Chakraborty, JoydeepOberley, RebeccaChalashkanov, NikolaObeso, AnaChaleckis, RomanasOborník, MiroslavChalioris, ConstantinObrador, ElenaChalupka, KarolinaObreza, AlešChamcheu, Jean ChristopherObuobi, Sybil Akua OkyerewaChami, GoyletteOcampo-García, Blanca ElíChampagne, BenoîtOchędzan-Siodłak, WiolettaChampagne, Pier AlexandreOchi, ShinichiroChampouret, YohanOchiai, BungoChan, Ben C. L.Ochoa-Reparaz, JavierChan, CatherineOchowiak, MarekChan, Chi OnOćwieja, MagdalenaChan, Yin-ChingOczkowicz, MariaChan, Yi-TsuOda, TakujiChan, Yu-YiOda, YukariChandra, Boggarapu PraphullaOdagi, MinamiChandramouli, KulshreshthaOdell, Luke R.Chandrasekaran, MurugesanOgale, Amod A.Chandrasekaran, Srinivas NiranjOgawa, KazumaChandru, KuhanOgawa, MitsutakaChandrudu, SaranyaOghumu, SteveChang, Chang-TangOgnyanov, ManolChang, Che-ChienOgórek, RafałChang, Cheng-ChungOgra, YasumitsuChang, Cheng-Wei TomOgungbe, IfedayoChang, Chia MingOh, Jae-MinChang, Chia-cheOh, KyungsooChang, Chien-ChungOh, SangphilChang, Chih-WeiOh, Seung-WonChang, Ching-yaoOhgane, KenjiChang, Chuang-RungOhisa, SatoruChang, Geng-RueiOhmae, EijiChang, Hai-ChouOhms, GiselaChang, Hsueh-WeiOhnishi, KohtaChang, Hsun-ShuoOhnishi, ShihoChang, Jui-ChengOhno, OsamuChang, Kun-CheOhta, KiminoriChang, KunokOhta, NobuoChang, Long-SenOhta, ShinjiChang, Sue-JoanOjeda-May, PedroChang, Te-ShengOjha, DeepakChang, Wei-JenOjima-Kato, TeruyoChang, Wen-WeiOjo, PeterChang, Yuan ShiunOjstršek, AlenkaChang, Yu-WeiOjwang, DicksonChao, Chih-HuaOkada, YoshiharuChao, Louis KuopingOkamoto, MasamiChapman, NadineOkamoto, TakayukiChaptal, VincentOkawara, ToruCharest, PascaleOkazawa, AtsushiCharitidis, Costas A.Oklinski, Michal Krystian EgelundCharmas, BarbaraOk-Seon, KwonChartier, AgnesOkuda, MasayukiChartoumpekis, DionysiosOkumura, MasakiChassande, OlivierOkunade, Adewole L.Chatel, GregoryOkuniewski, AndrzejChatgilialoglu, ChryssostomosOláh, AttilaChatterjea, DevananiOláh, JuliannaChatterjee, AbhijitOláh, ViktorChatterjee, RuchiraOlar, RodicaChatterjee, SudiptaOlaru, MihaelaChatzaki, EkateriniOlaru, Octavian TudorelChatzidoukas, ChristosOlech, MartaChatzifragkou, AfrodotiOlejar, Kenneth J.Chaud, MarcoOlejnik, AnnaChauhan, NeerajOlennikov, DaniilChaurasia, Akhilesh KumarOleszek, WieslawChava, Rama KrishnaOlewnik-Kruszkowska, EwaChaves, SílviaOlga, PapadopoulouChavez, Carlos A. SanhuezaOliani, Sonia MariaChavez, FermanOliva, AlexisChavez-Calvillo, GabrielaOliva, JoséChavez-Santoscoy, Rocio AlejandraOliva, RominaChawla, AseemOliveira, Alcineia C.Checconi, PaolaOliveira, AnaChechik, VictorOliveira, AndreiaCheel, JoséOliveira, Guedmiller S.Chekmenev, EduardOliveira, Hugo M.Chellan, NireshniOliveira, JoanaChelli, RiccardoOliveira, José M.Chemat, FaridOliveira, M. ConceiçãoChen, BingdiOliveira, Maria ConceiçãoChen, BinlingOliveira, Paula A.Chen, BolinOliveira, Rui S.Chen, CharlesOliver, William ThomasChen, Chau-ChyunOliveri, PaoloChen, ChiOlivero-Verbel, JesusChen, Chi-TienOliviero, FrancescaChen, Chuan-LinOliviero, GiorgiaChen, Chung-HwanOlivos-Oré, Luis AlcidesChen, Chun-JungOliwa, RafałChen, Chun-LinOllero, MarioChen, GangOllevier, ThierryChen, GuangpingOlmo, Luis Rojo DelChen, GuoxunOlmo-García, LucíaChen, HaixiaOlofinjana, AyodeleChen, HaoOlorunniji, Femi J.Chen, Hao PingOlson, David E.Chen, HaoqingOlson, Thomas L.Chen, HongzhangOlubiyi, Olujide O.Chen, Hsin-ChunOlyaee, SaeedChen, Hsing-YinOmar, Ahmad A.Chen, Hsuan-YingOmar, Syed HarisChen, Hung-YuOmichi, MasaakiChen, James KOmidian, KosarChen, Jeff Yi-fuOmura, JunichiChen, Jen-TsungOnčák, MilanChen, Jhy-DerOnchoke, KefaChen, JiahaoOnduka, ToshimitsuChen, JianboOng, Eng ShiChen, Jing-HsienOng, Hwai ChyuanChen, JunOniga, IlioaraChen, KaihongOniga, Smaranda DafinaChen, Kelvin H.-C.Oniszczuk, AnnaChen, LiOniszczuk, TomaszChen, Liang-YuOnnis, ValentinaChen, Lih-GeengOno, KenjiChen, LiliOnyeagucha, Benjamin ChidiChen, LinOnyenwoke, Rob UcheChen, LingxinOnys’ko, PetroChen, Mei-ChuanOostenbrink, ChrisChen, MingliOpaliński, ŁukaszChen, MingshengOpatz, TillChen, MingzhouOpekar, FrantišekChen, MinjianOpitz, JörgChen, Mu-KuanOppedisano, FrancescaChen, Po-WenOprea, Corneliu IoanChen, QiOprea, OvidiuChen, Qiao-HongOpricǎ, LăcrămioaraChen, QixianOpsenica, IgorChen, Shiu-NanOracz, JoannaChen, Tao-HsingOrbach, RonChen, WeiOrczyk-Pawiłowicz, MagdalenaChen, Wei-TingOrdaz-Ortiz, José JuanChen, Wen-YingOrdóñez García, SalvadorChen, Xiao-LanOrdoudi, StellaChen, XiaoxinOrellana-Palma, PatricioChen, XinOreshonkov, AleksandrChen, XinjianOrfila, CarolineChen, XiujuanOrian, LauraChen, Xue-GangOrinska, ZaneChen, YehOrkusz, AgnieszkaChen, Yen-HauOrlando, GiustinoChen, Yi-FanOrlić, SandiChen, YijiaOrlova, AnnaChen, YuOrłowski, MarekChen, Yung-ChungOrmeci, AlimChen, Yun-JuOrobtchouk, RegisChen, ZhihangOrodnez, MarioChen, ZhijunOroian, Mircea AdrianChen, ZhongningOrooji, YasinChénais, BenoitOrosa, José A.Cheng, Chih-ChiaOrozco, JahirCheng, Fong-YuOrso, EvelynCheng, GangOrtega Castro, JoaquínCheng, Juei-TangOrtega, DanielCheng, Jya-WeiOrtega, María JesúsCheng, PeiOrtega, NatividadCheng, QingqingOrtega, PaulaCheng, QunkangOrtega-Alfaro, María CarmenCheng, Sen-SungOrth, James D.Cheng, TaoOrtigueira, JoanaCheng, XiaodongOrtiz Martínez, Víctor ManuelCheng, XingguoOrts, JulienCheng, XinlaiOrynycz, OlgaCheng, Yi-ShengOrzelska-Górka, JolantaCheng, Yuan-BinOsakada, KohtaroCheng, Yu-JungOsakada, YasukoCheon, Dong-JooOsaki, TomohiroCheong, Yuen-KiOsborn, HelenCheraghi Bidsorkhi, HosseinOsborn, Helen M.i.Cheraghian, GoshtaspOsés, SandraCheret, JérémyOshima, YusukeCherkaoui Malki, MustaphaOsma, JohannChermette, HenryOsman Ahmed, AhmedChernikov, OlegOśmiałowski, BorysChernova, TatianaOsório, Wislei RCheshenko, NataliaOspina-Millán, Claudia A.Chetcuti, Michael J.Osses, NelsonChetwynd, AndyOstacolo, CarmineCheynier, VéroniqueOstadhassan, MehdiChi, GeraldOstoa-Saloma, PedroChianese, GiuseppinaOstojic, SergejChiang, Anthony S. T.Ostrauskaite, JolitaChiang, Chien-MinOstrowska, KingaChiang, Chi-LingOstrowski, MaciejChiang, Hsiu-MeiOtero, José A.Chiang, Wen-DeeOtero, MartaChiang, Ya-YuOtero-Espinar, Francisco JavierChiarella, EmanuelaOthman, Eman M.Chiarelli, LaurentOtmačić Ćurković, HelenaChiarini, MarcoOtomo, TakanobuChiavaroli, AnnalisaOtřísal, PavelChichorro, Juliana G.Otsuka, MasamiChien, Hsiu-WenOtsuka, YuzuruChien, Shih-ChangOtte, Andrew D.Chien, Yi-wenOttria, RobertaChifiriuc, CarmenOuellet-Plamondon, ClaudianeChifiruc, Mariana CarmenOuguerram, KhadijaChilibeck, PhilOuvrard, AimericChilton, LisaOuyang, LiliangChimenti, IsottaOvando Martínez, MaribelChimienti, Guglielmina AlessandraOven, PrimozChin, Young-wonOvergaard, JacobChinchilla, RafaelOves-Costales, DanielChinnathambi, ShanmugavelOvidi, ElisaChiou, Chun-TangOvissipour, RezaChirgwin, John M.Owczarz, PiotrChiriac, AuricaOyama, DaiChirila, TraianOyedeji, AdebolaChis, VasileOzarowski, MarcinChisaka, MitsuharuOzawa, KoichiroChisholm, John D.Ozawa, ShogoChistyakov, Vladimir AnatolievichOzawa, YoshikiChisu, ValentinaP. Busardó, FrancescoChitchumroonchokchai, ChureepornPacelli, SettimioChitescu, Carmen LidiaPacetti, DeborahChitgupi, UpendraPachauri, VivekChittori, SagarPacheco, NeithChiu, Chih-WeiPacheco-Catalán, DaniellaChiu, Ching-FengPacheco-Fernández, IdairaChiummiento, LuciaPacheco-Londoño, Leonardo C.Chizhov, Alexander O.Paci, MaurizioChizzola, RemigiusPacia, Marta Z.Chlichlia, KaterinaPacifici, RobertaChmielarz, LucjanPacifico, SeverinaChmielarz, PawełPaciorek-Sadowska, JoannaChmielewski, TomaszPacios, Luis F.Cho, Dae WonPaczesny, JanCho, Deok-YongPaczkowski, JonCho, Dong-WooPadilla-Martínez, Itzia I.Cho, Hea-YoungPadilla-Zakour, OlgaCho, Hyun-JongPadmanabhan, VenkatCho, Jae YoulPadnya, PavelCho, Sang WanPadrão, JorgeCho, SunghunPadro, Juan M.Cho, Yi TzuPaduch, RomanChobot, VladimírPadula, Matthew P.Chocholouš, PetrPaeng, KijungChoi, HyukjaePaepegaey, Anne-CécileChoi, Hyung-KyoonPagacz, JoannaChoi, InsungPagani, StefaniaChoi, Jae-HongPaganin, Valdecir AntonioChoi, Jane RuPagano, AlessandraChoi, Jeong-WooPagano, BrunoChoi, JonghoonPagano, CinziaChoi, JongminPagliara, StefanoChoi, Nag JungPagotto, SaraChoi, SeungkeunPaik, YounkeeChoi, Tae GyuPailleret, AlainChoi, Young HaePaim, LeonardoChoi, Young HeePaixão Cansado, Isabel PestanaChoi, Young WhanPaixao Coelho, Jose AugustoChojnacki, JerzyPaizs, CsabaCholewinski, GrzegorzPajak, BeataChoquesillo-Lazarte, DuanePajchel, ŁukaszChorazy, SzymonPajeva, IlzaChorna, NataliyaPak, ChanhoChoromanska, AnnaPakdel, FarzadChoshi, TominariPakhomova, SvetlanaChou, C. JamesPal, AnimeshChou, WeichunPal, RumpaChouhan, Raghuraj SinghPalacio, EdwinChow, James C. L.Palacios, JavierChow, Simon Kwoon-HoPalacios-Santander, José MaríaChowdhury, Abu Faem Mohammad AlmasPaladino, AntonellaChowdhury, PankajPalage, MarianaChowdhury, RatulPalamà, IlariaChrcanovic, BrunoPalanti, SabrinaChreptowicz, KarolinaPalavai, Sripal ReddyChristensen, Jørn B.Palchoudhury, SoubantikaChristensen, Lars P.Palcut, MariánChristina, Barja-FidalgoPalenzuela López, José AntonioChristodoulou, Maria-IoannaPalermo, AmeliaChristoffer Eklund, PatrikPalese, Luigi LeonardoChrobak, ElwiraPalinko, IstvanChronakis, NikosPall, EmokeChronopoulou, EvangeliaPallàs Lliberia, MercèChronopoulou, LauraPalma, EleonoraChruszcz, MaksymilianPalma, MiguelChrysargyris, AntoniosPalmer, AlanChrzanowski, ŁukaszPalmieri, AlessandroChu, Chun HungPalmieri, ErikaChu, Pei-MingPalmieri, ValentinaChu, XiangpingPalomba, LetiziaChuang, Er-YuanPalomba, RobertoChubarov, AlexeyPalombi, LauraChueh, Anderly C.Paltinean, RamonaChueh, Pin JuPalumbo, Giuseppe AlbertoChun, Jong TaiPalumbo, OrieleChun, Kyung-SooPaluszczak, JarosławChung, Ching-HuPalyi, GyulaChung, Dong YoungPalyulin, VladimirChung, Doo SooPan, JingChung, GehoonPan, JinheChung, Sheng-HengPan, Po-shenChung, Suk-JaePan, QuanwenChung, YeonseokPan, ShanlinChurio, MariaPan, Yuan JiangChuyen, Hoang V.Panagiotidis, Mihalis I.Chwalek, KarolinaPanaro, Maria AntoniettaChwalibog, AndréPanayiotou, CostasChworos, ArkadiuszPanchal, SatyamChyau, Charng-CherngPanchapakesan, BalajiChylińska, MartaPandele, MadalinaCiampi, SimonePanderi, IreneCiancaleoni, GianlucaPandey, SantoshCiancetta, AntonellaPandit, SantoshCianfaglione, KevinPandíțh, AnúpCiardelli, FrancescoPandya, Pankita H.Ciardiello, Maria AntoniettaPanek, JaroslawCiarlo, LauraPanek, JarosłąwCiasca, GabrielePaneth, AgataCiccarelli, RenataPaniagua, CandelasCicchi, StefanoPanić, ManuelaCicco, NunziaPanigati, MonicaCiccone, MarcoPannerselvam, SureshÇiçek, Serhat SezaiPannkuk, EvanCicenas, JonasPansare, Sunil V.Cicero, ArrigoPantazis, Dimitrios A.Cichero, ElenaPantić, MilicaCielecka-Piontek, JudytaPanzella, LuciaCiesielski, WojciechPao, Chun-WeiCieśla, JolantaPaola, D’ArrigoCieslar, GrzegorzPaolella, AndreaCieslik, WioletaPaolesse, RobertoCifone, Maria GraziaPaoli, PaoloCilla, AntonioPaolillo, MayraCillo, FabrizioPaolino, MarcoCimas, Francisco J.Paolmino, OlgaCimino, RobertoPaone, AlessioCimpoiu, ClaudiaPap, JózsefCinelli, PatriziaPapaccio, GianpaoloCinteza, Otilia LudmilaPapachristodoulou, AlexandrosCiobica, AlinPapadakis, RaffaelloCiogli, AlessiaPapadimitriou, VassilikiCiolacu, DianaPapadopoulos, Antonios N.Ciornea, Elena TodirascuPapadopoulos, Christodoulos E.Čipak Gašparović, AnaPapadopoulos, DimitriosCipak, LubosPapageorgiou, Anastassios C.Cipakova, IngridPapagiannitsis, Costas C.Cipullo, RobertaPapagiannopoulou, DionysiaCirciumaru, AdrianPapaioannou, DionissiosCircu, ViorelPapakyriakou, AthanasiosCirillo, EmiliaPapamichael, Emmanuel M.Cirillo, GiuseppePapanastasiou, IoannisCirmi, SantaPapathanasiou, FokionCirri, DamianoPapatriantafyllopoulou, ConstantinaCirrincione, GirolamoPapetti, AdeleCirtoaje, CristinaPapi, AlessioCirujano, FranciscoPapież, MonikaCiszewski, WojciechPaplinska, MagdalenaCitores, LuciaPapoti, Vassiliki T.Ciufolini, Marco A.Pappa, AglaiaČižmár, ErikPappalardo, DanielaČizmić, MirtaPappas, ChristosClaramunt, Rosa M.Paquete, Catarina M.Claridge, Jolyon K.Parada, FabiánClark, BenjaminParadela, AlbertoClark, GeorginaParadowska-Gorycka, AgnieszkaClarke, Anthony J.Paramythiotis, SpirosClarke, William AlexPardío-Sedas, Violeta T.Claudia, Delgadillo PugaPardo, EmilioClaudio, TrapellaParedes, AdrianClaus, HaraldParedes, Alejandro JavierClavijo-McCormick, AndreaParedez, AlexanderClegg, Jack K.Parenti, CarmelaClément, GillesParfenova, HelenaClement, YuenParida, Asish K.Clemetson, Kenneth J.Parida, SheetalClericuzio, MarcoParikesit, Arli AdityaCoates, DawnParisi, FilippoCobb, Steven L.Parizad, Parisa AbbasiCocco, LucioPark, Byung-WookCocero Alonso, María JoséPark, Chul-hoCocklin, SimonPark, ChulhunCocovi Solberg, David JaimePark, DaehoCoderch, ClairePark, DaesungCodina, GeorgianaPark, Deog-SuCoelho-Silva, JuanPark, Dong HyukCoetzer, TheresaPark, Dong SunCohen, BoikoPark, Han-ACohen, YachinPark, Hee-SooCojocaru, CorneliuPark, Hyun-WooColangelo, Anna MariaPark, Il-KwonColavito, Sierra A.Park, InkyuColby, DavidPark, Jae-HyungColdea, Teodora EmiliaPark, JeewoongColina-Márquez, JosePark, Jeong-HunColl, JosepPark, JeyoungCollet, TrudiPark, Ji-WonCollina, SimonaPark, JongseungCollinge, David B.Park, Joon SikColomban, PhilipePark, Ki TaeColombo, DiegoPark, Kyoung-ChanColonna, BiancaPark, Kyung-MokColonna, GiovanniPark, MinČolović, Mirjana B.Park, MiraColtelli, Maria BeatricePark, Myung HwanComan, Simona M.Park, Sang KiComan, VasilePark, SangmoonComas-Garcia, MauricioPark, SangwonCombès, AudreyPark, SolmoiComfort, Kristen K.Park, Soo JeanComito, Robert J.Park, SungsuCommisso, MauroPark, WansuComparat, DanielPark, WooramConan, FrancoisePark, YaewonConcellon, AlbertoPark, Yoo MinConcilio, SimonaPark, YoonsuConconi, Maria TeresaPark, Young-SeoCondelli, NicolaPark, Young-TaeCondello, MariaParker, Linda A.Condorelli, Guglielmo GuidoParlog, RalucaConejero, SalvadorParmeggiani, FabioConforti, FilomenaParpinello, GiuseppinaCongBao, KangParra, MargaritaConnor, MarkParra-Saldívar, RobertoConran, JohnParrella, EdoardoConstantin, LucianParrino, BarbaraConstantinescu-Aruxandei, DianaParrino, FrancescoConstantinides, ChristosParrotta, ElviraConstantinou, ViolettaParrotta, LuigiConsumi, MarcoParsaeimehr, AliContardi, MarcoParsons, JasonContel, MariaPârvu, Alina ElenaConterosito, EleonoraPârvu, MarcelConti, ClaudiaPasa, AndreConti, PioPasán, JorgeContiero, JonasPasbakhsh, PooriaContreras-Cornejo, Hexon AngelPascali, GiancarloCoombs, MelaniePasche, BorisCooper, RobinPaseiro-Cerrato, RafaelCopeland, LesPasetto, MarcoCopolovici, Dana MariaPasini, DarioCopolovici, LucianPaskaleva, AlbenaCoppedè, FabioPasquinelli, GianandreaCoradin, ThibaudPassamonti, SabinaCorallo, ClaudioPassarella, DanieleCorchado, Jose C.Pasternak, TarasCordaro, MarikaPašti, Igor A.Cordeiro, Ana SaraPastor, VictoriaCordero, Chiara EmiliaPastore, CarloCordero-Lanzac, TomasPastore, RobertoCórdoba, ManuelPastuch-Gawolek, GabrielaCórdoba-Diaz, ManuelPaszkiewicz, MonikaCorke, HaroldPaszkiewicz, SandraCornea-Cipcigan, MihaielaPatankar, Jay V.Cornejo, AlbertoPatarata, LuísCornelis, MarilynPate, F. DonaldCornett, ClausPateiro, MirianCornu, AgnesPatel, AlokCoroaba, AdinaPatel, Ankit B.Corradini, ValdisPatel, BhargavCorrea Pacheco, Zormy NacaryPatel, NilkanthCorrea, ArkaitzPatenge, NadjaCorrêa, Arlene GonçalvesPateraki, IriniCorrêa, Camila RenataPaterson, BrettCorrea, Diego Rafael NespequePathak, PawanCorrecher, VirgilioPatience, GregoryCorreia, Ana CristinaPatra, Jayanta KumarCorreia, João D. G.Patriarchi, TommasoCorreia, ManuelaPatruno, AntoniaCorreia-de-Sá, PauloPattammattel, AjithCorsaro, CarmeloPattison, GrahamCorsaro, Maria MichelaPauer, WernerCorsi, IlariaPaul, AnanyaCorte, LauraPaul, ArghyaCortés, CristóbalPaul, SanjoyCortés, Farid B.Paul, TitanCortiella, Mariona GilPauleta, SofiaCortijo, MiguelPauliukaite, RasaCorvis, YohannPaulo, AntónioCorvo, Marta C.Paulson, JoelCorzana, FranciscoPaulus, BeateCosco, DonatoPau-Roblot, CorinneCoscueta, EzequielPautz, AndreaCosenza, GianfrancoPavagadhi, ShrutiCosimbescu, LeliaPavanello, ChiaraCoskun, Ahmet F.Pavao, Mauro SergioCosma, PinalysaPavel, Octavian DumitruCosme, FernandaPavela, RomanCossairt, Brandi M.Pavelic, Sandra K.Cossignani, LinaPaventi, GianlucaCossio, FernandoPavic, ValentinaCosta, AnaPavlić, BranimirCosta, CorradoPavlos, KlepetsanisCosta, David SchellenbergerPavlov, Georges M.Costa, GiosuèPavlov, Youri ICosta, LucaPavlovic, RadmilaCosta, Marina C.Pawar, Shrikant D.Costa, Paulo JorgePawar, SnehalataCosta, PedroPawlak, AndrzejCosta, RosariaPawlicki, MiłoszCosta, RubenPawluć, PiotrCosta, RuiPawyta, MiroslawaCosta, StefaniaPayan-Carreira, RitaCosta, Susana P. G.Paz, CristianCostabile, GabriellaPazdzior, KatarzynaCostantini, AnielloPaz-Ferreiro, JorgeCostantini, SusanPazourek, JiříCostanzo, PaolaPeana, Massimiliano F.Costas, MiguelPecina, PetrCoste, FranckPecinová, AlenaCostea, TeodoraPeczuh, MarkCostello, Ben De LacyPedatella, SilvanaCosti, Maria PaolaPedersen, HenrikCostinel, DianaPedraza Chaverri, JoséCostisor, OtiliaPedretti, AlessandroCotelle, PhilippePeelman, FrankCotillas, SalvadorPeeters, MarloesCouderc, FrançoisPeethambaran, BelaCoulouarn, CédricPei, YonggangCourdavault, VincentPei, ZhiqiangCouris, SteliosPei, ZingwayCoutinho, Henrique Douglas MeloPeiretti, Pier GiorgioCouto, CristinaPeixe, Tiago SeveroCovino, RobertoPeixoto, Maria JoãoCox, JamesPeiyan, ShenCox, Jonathan A. G.Pék, ZoltánCoy-Barrera, EricssonPekar, MiloslavCoz, AlbertoPeleteiro, MercedesCozza, GiorgioPelidou, Sygkliti-HenriettaCozzi, CristinaPelka, RobertCozzolino, DanielPellegrin, YannCragg, PeterPellegrini, CarolinaCran, Marlene J.Pellegrini, MarikaCranfield, CharlesPellegrino, Roberto MariaCrans, DebbiePellegrino, SaraCrauste, CélinePellei, MauraCravero, Maria CarlaPellico, JuanCravotto, GiancarloPelosi, GiorgioCrawford, JagodaPeluffo, HugoCrawford, Philip W.Peluso, IlariaCreager, Stephen E.Peluso, PaolaCreary, XavierPeña Fernández, María ÁngelesCrescenzi, ElviraPeña-Amaro, JoséCrescenzi, OrlandoPeñalver, RosaCrespi, StefanoPeña-Rodríguez, Luis ManuelCrespo, IrenePenas, Sonia EirasCretoiu, DragosPenesova, AdelaCreutz, SidneyPeng, BingyinCrezee, JohannesPeng, Robert Y.Criado-Gonzalez, MiryamPeng, Shu-FenCrinelli, RitaPeng, Wen-HuangCrișan, GianinaPeng, ZhiliCrisan, LuminitaPenn, RaymondCrisp, Ryan W.Penna, ClaudiaCrispi, StefaniaPentzold, StefanCristea, GabrielaPepi, MilvaCristiano, Maria De LurdesPeptu, CristianCristofolini, LuigiPeral, AngelCristóvão, BeataPerale, GiuseppeCrivii, CarmenPerálvarez-Marín, AlexCroatt, Mitchell P.Perbellini, LuigiCrocetti, LetiziaPerche, FédéricoCrochet, AurélienPercino, JudithCross, Carroll E.Percival, Susan S.Crucho, Carina Isabel CorreiaPerdih, AndrejCrupi, PasqualePerdih, FrancCrupi, RosaliaPerdomo, JoseCruz, RodrigoPerego, CarlaCruz, Rui M. S.Pereira, CarlaCruz-Rivera, EdwinPereira, DavidCryle, MaxPereira, ElianaCsámpai, AntalPereira, FilipeCsapo, EditPereira, FlorbelaCsávás, MagdolnaPereira, Giuliano EliasCsiszár, AgnesPereira, JorgeCsóka, IldikóPereira, José AlbertoCsősz, ÉvaPereira, José AugustoCsuk, RenéPereira, LeonelCuadrado, CarmenPereira, LeonorCuccovia, Iolanda MideaPereira, LuizCucinotta, FrancisPereira, Maria CarmoCuesta, RafaelPereira, Maria De LourdesCui, Hai-yingPereira, Maria G.Cui, HuaqingPereira, Nelson A. M.Cui, HuiPereira, Olívia R.Cui, JingnanPereira, PatriciaCui, QingbinPereira, RenatoCui, YuhaiPereira, RuiCui, ZhengPereira-Leite, CatarinaCui, ZhisongPereira-Maia, EleneCummins, AdrianPerera, LalithCundari, Thomas R.Perera, RushikaCuny, Gregory D.Peres, António M.Cuomo, FrancescaPerestrelo, RosaCurcio, ManuelaPerestrelo, Rosa Maria De SáCurrò, MonicaPerez Castillo, YunierkisCurti, ClaudioPérez De Vega, María JesúsCurtis, Anthony D. M.Perez Del Real, RafaelCurtis, MarionPérez Pérez, MaríaCurulli, AntonellaPérez Puyana, Víctor ManuelCutignano, AdelePerez Reyes, CarolinaCutone, AntimoPérez Sestelo, JoséCuya, Ricardo TeobaldoPérez, Ana G.Cuzzocrea, SalvatorePérez, AntonioCvrček, LadislavPérez, ElíasCwalina, BeataPerez, Juan J.Cyboran-Mikołajczyk, SylwiaPerez, LaurentCycoń, MariuszPerez, RaphaelCyplik, PawełPerez, SergeCypryk, MarekPérez-Álvarez, Jose AngelCysewski, PiotrPérez-Casas, SilviaCytlak, UrszulaPérez-Gálvez, AntonioCzarnecki, Mirosław AntoniPérez-García, SeleneCzech, BożenaPérez-Jiménez, JaraCzechowski, TomaszPérez-Lozano, PilarCzekala, WojciechPérez-Mendoza, Manuel J.Czekelius, ConstantinPerez-Sayans, MarioCzekster, Clarissa M.Perfetti, MauroCzernek, JiříPergher, Sibele B. C.Czerwińska, EwaPérigo, Elio AlbertoCzerwińska, MonikaPerin, GiorgioCzerwiński, MarcinPerinelli, Diego RomanoCzogalla, AleksanderPerioli, LuanaCzugler, MátyásPeris, José-EstebanCzyż, JarosławPeriyasamy, PalsamyCzyżowska, AgataPerkins-Veazie, PenelopeD. Bortner, CarlPerkovic, IvanaĐ. Glišić, BiljanaPerlepes, SpyrosD. Škrbić, BiljanaPerles, JosefinaD’Abramo, MarcoPerlikowski, DawidD’Agostino, CarminePerlin, MichaelD’Alessandro, WalterPermyakov, EugeneD’alonzo, DanielePerna, FilippoD’Amato, Chiara AnnaPerna, GiuseppeD’andrea, CristianoPerni, MicheleD’Angelo, MichelePero, RaffaelaD’Angelo, StefaniaPeron, GregorioD’Anna, FrancescaPerova, Tatiana S.D’Antona, NicolaPerreau, FrancoisD’Archivio, Angelo AntonioPerret, AlainD’Arcy, PadraigPerrin, Charles L.D’Auria, John C.Perrin, LaraD’Auria, Maria ValeriaPerrone, DanielaD’Auria, MaurizioPerrucci, StefaniaD’Errico, StefanoPerry, RobinD’Onofrio, LucaPersuric, ZeljkaD’Onofrio, NunziaPerteghella, SaraD’Orazio, GiovanniPerugini, PaolaD’Orsi, BeatricePerut, FrancescaD’Ursi, Anna MariaPes, Giovanni MarioD’Ursi, PasqualinaPescina, SilviaD’Urso, AlessandroPessela João, BenevidesDa Costa Ribeiro, Ana PaulaPestov, AlexanderDa Costa, Ana Maria Dos Santos RosaPestryakov, AlexeyDa Cunha, Mário Antônio AlvesPetcu, CristianDa Pieve, ChiaraPetelin, AndrejDa Silva Júnior, Arnóbio AntonioPeter, AncaDa Silva Júnior, Eufrânio N.Peter, FranciscDa Silva, Emerson RodrigoPeter, KarinDa Silva, Jaime Pedro OliveiraPeter, MartinDa Silva, Luís C. N.Peters, JudithDa Silva, Luis Cláudio NascimentoPeters, VerenaDa Silva, Maria D. M. C. RibeiroPeterson, Eric CharlesDa Silva, Victor Diogenes AmaralPeterson, JuliaDa Silva, WiltonPeterson, LarrynDa Veiga Junior, Valdir FlorencioPetit, ChristopheDa’na, EnshirahPetit, Patrice X.Daberdaku, SebastianPetkoski, SpaseDąbrowska, MonikaPetkova, NadezhdaDąbrowski, WojciechPetkovic, HrvojeDaca, AgnieszkaPetkovic, MilenaDacarro, GiacomoPetkowski, Janusz J.Dadachova, EkaterinaPetrásková, LucieDadáková, KateřinaPetriccione, MilenaDagmara, MalinaPetrikaite, VilmaDagnac, ThierryPetrillo, GiovanniDahan, AlbertPetrini, MarinoDahiya, RajivPetroni, KatiaDahl, MortenPetronilho, AnaDai, ChongshanPetropoulos, SpyridonDai, LizongPetrosino, MariaDai, YuminPetrou, ChristosDaiguebonne, CarolePetrov, DmitryDakshanamoorthy, ArivuoliPetrova, Krasimira T.Dal Corso, AlbertoPetrović, BiljanaDal Piaz, FabrizioPetrović, MarijaDal Prá, IlariaPetrucci, RitaDalgarno, ScottPetrucci, RobertoDalhaimer, PaulPetruczynik, AnnaDall’Acqua, StefanoPetruzzelli, GianniantonioDalla Pozza, ElisaPetruzzi, LeonardoDalla Via, LisaPetti, CarloalbertoDallavalle, SabrinaPettinari, ClaudioDalle Vacche, SaraPetuhov, OlegDalmoro, AnnalisaPetzold, GuillermoDalpozzo, RenatoPeuronen, AnssiDama, MuraliPeveler, WilliamDamalanka, VishnuPeyrottes, SuzanneDamasco, JossanaPeyton, David H.Damberger, FredPezzani, RaffaeleDambolena, José S.Pezzato, LucaDamha, Masad J.Pezzella, AlessandroDamiano, FabrizioPezzella, CinziaDamkaci, FehmiPezzuto, AldoDamodaran, Krishna K.Pfattner, RaphaelDams-Kozlowska, HannaPfister, BarbaraDan, DobrotaPhadtare, VarshaDanac, RamonaPham, ThaoDanaher, MartinPhan, Chin-SoonDanao, Mary-Grace C.Phan, Thi Tuong VyDance, IanPhapale, Prasad B.Danciu, CorinaPhilipp, ManfredDandawate, PrasadPhilippe, AllanDandlen, Susana AnahiPhilips, NeenaDangal, ShreePhillips-Jones, Mary K.Dangles, OlivierPiao, XijunDangol, ManitaPiasecka, AnnaDaniel, Morales CanoPiątczak, EwelinaDaniela, JakšićPiatkevich, KirylDanielak, DorotaPiątkowski, MarekDaniel-da-Silva, Ana LuísaPicariello, GianlucaDaniels, RolfPicchio, RodolfoDanielson, NeilPiccialli, GennaroDanijela, BognerPiccionello, Antonio PalumboDanilo, Belviso BennyPiccirillo, ClaraDanilov, Sergei M.Piccolella, SimonaDanjou, Pierre-EdouardPicerno, PatriziaDanko, MartinPichini, SimonaDannaoui, EricPichler, VerenaDansette, Patrick M.Pichon, CélineDante, SilviaPickering, Andrew M.Darabantu, MirceaPickford, RussellDaran, Jean-ClaudePico, YolandaDarchen, AndréPicone, DeliaDarder, MargaritaPidot, Sacha J.Dardonville, ChristophePiechowiak, TomaszDarenskaya, M. A.Pieczywek, Piotr M.Darie-Nita, Raluca NicoletaPiedade, Ana PaulaDaroch, MaurycyPiegat, AgnieszkaDarras, AnastasiosPiekoszewski, WojciechDarzi, SaeedehPiel, MarkusDas, PradipPielech-Przybylska, KatarzynaDas, RupaliPielichowski, KrzysztofDatta, RupsaPiemonte, VincenzoDatta, SandipanPiemontese, LucaDatta, ShubhashisPieraccini, StefanoDaugrois, Jean-HeinrichPieretti, StefanoDavalieva, KatarinaPiergiovanni, LucianoDavank, VadimPierini, FilippoDavarpanah, AfshinPiermatti, OrianaDavid, Gabriela IuliaPierson-Wickmann, Anne-CatherineDavid, VictorPierzynowski, StefanDavidescu, Corneliu-MirceaPietropolli Charmet, AndreaDavies, MichaelPietrzyk, BozenaDavinelli, SergioPietrzyk, PiotrDavis, FrankPietrzyk-Brzezinska, Agnieszka J.Davis, FredPietschnig, RudolfDavis, Matthew C.Piga, IsabellaDavydova, ViktoriyaPigge, F. ChristopherDawe, LouisePigliacelli, ClaudiaDawn, ArnabPignataro, BrunoDayen, Jean FrançoisPignitter, MarcDayneko, SergeyPiiper, AlbrechtDaza-Navarro, PaulaPike, Nicholas A.De Agostini, ArianePiktel, EwelinaDe Almeida, Roberto FarinaPilarska, Agnieszka A.De Almeida, Wagner BatistaPileio, GiuseppeDe Alvarenga, Elson SantiagoPilepić, ViktorDe Amici, MarcoPilic, BrankaDe Araújo, Daniele RibeiroPilkington, LisaDe Araujo, ElvinPilla, VivianeDe Assis, Leonardo V. M.Pilling, DarrellDe Bellis, LuigiPilo, Maria I.De Benedetto, GiuseppePilo-Pais, MauricioDe Berardis, DomenicoPiluso, SusannaDe Brevern, Alexandre G.Piluzza, GiannellaDe Bruijn, Henriette S.Pimienta, Rodney LacretDe Carvalho Santos Ebinuma, ValériaPinakoulaki, EftychiaDe Carvalho, Rosemary AparecidaPinalli, RobertaDe Castro Ramalho, TeodoricoPinar, AnitaDe Castro, Gabriela S.Pindelska, EdytaDe Castro, SoniaPineda Rodríguez, TeresaDe Ceballos, Maria L.Pinedo-Rivilla, CristinaDe Clercq, ErikPinela, JoséDe Coninck, JoëlPinent Armengol, MontserratDe Crisci, AntonioPiñero, Jose CarlosDe Deurwaerdere, PhilippePiñero, ManuelDe Diego, MartaPingarrón, JoséDe Falco, BrunaPinheiro, MarinaDe Falco, EnricaPinho, LuísDe Falco, ValentinaPini, ElenaDe Feo, VincenzoPini, ValerioDe Filippis, BarbaraPinotti, LucianoDe Giglio, ElviraPintea, AdelaDe Giorgi, Maria LuisaPinti, MarcelloDe Giorgio, FrancescaPinto, Diana CláudiaDe Giudici, GiovanniPinto, ErnaniDe Graaf, CoenPinto, EugéniaDe Jonghe, StevenPinto, JavierDe Julián Ortiz, JesusPinto, SaraDe Kruijff, Robin M.Pintor, ArianaDe La Fuente, HortensiaPintus, Gianfrancode la Fuente-Núnez, CésarPinzaru, IuliaDe La Garza, MireyaPinzi, LucaDe La Luz Garcia-Hernandez, MariaPiosik, JacekDe La Rosa, Laura AlejandraPiotrowska, UrszulaDe La Torre, GemaPiotrowski, PaulinaDe La Vega, José Manuel GarcíaPiotrowski, TomaszDe Lima Nobre, Marcos AugustoPiotto, Stefano P.De Lima, Valéria Marçal FelixPiovesan, DamianoDe Luca, GiuseppinaPiper, BrianDe Luca, PierantonioPirani, FernandoDe Luca, StefaniaPires, Regina HelenaDe Marco, FedericoPiret, JocelyneDe Martin, RainerPiriou, YannickDe Masi, LuigiPirman, TatjanaDe Matteis, ValeriaPisana, SimoneDe Meijer, MariPisaneschi, FedericaDe Melo, Marcelo M. R.Pisani, Didier F.De Mendonça, Dina I. M. D.Pishgar, RoyaDe Mey, GilbertPisk, JanaDe Miranda, João Antônio LealPiska, KamilDe Munno, GiovanniPisklak, Dariusz MaciejDe Oliveira Lobo, AndersonPispas, StergiosDe Oliveira, Helinando PequenoPitsikalis, MarinosDe Oliveira, José Miguel P. FerreiraPitsinos, EmmanuelDe Oliveira, Kléber T.Pittelkow, MichaelDe Oliveira, Marcos RobertoPitto-Barry, AnaïsDe Oliveira, Paulo RenatoPitucha, MonikaDe Oliviera Silva, ElianePiubelli, ChiaraDe Palma, LauraPivac, NelaDe Pasquale, ClaudioPivetta, TizianaDe Paula, Haroldo César BeserraPiwowarski, Jakub P.De Paz, Antonio TaberneroPizzi, AntonioDe Riccardis, FrancescoPizzo, ElioDe Rosa, Igor M.Pizzuti, LucasDe Rosa, MariaPlaczek, William J.De Rosa, Maria CristinaPlagge, JanDe Santo, Maria PenelopePlapp, BryceDe Silva, FranklynPlasseraud, LaurentDe Simone, ClaudioPlastina, PierluigiDe Simone, SalvatorePlatas-Iglesias, CarlosDe Simone, Salvatore GiovanniPlatella, ChiaraDe Sousa, Damião PergentinoPlatta, HaraldDe Sousa, Frederico B.Platts, JamesDe Souza Gil, EricPlavec, JanezDe Souza, Danival JoséPlaza, Encarna GomezDe Stefano, VitaPłaza, GrażynaDe Tommasi, NunziatinaPlaza-Díaz, JulioDe Vendittis, EmmanuelePlazinski, WojciechDe Villiers, Melgardt M.Plech, TomaszDe Vincentis, GiuseppePlenis, AlinaDe Visser, SamuelPleshko, NancyDe Vleeschouwer, FreijaPletsa, VassilikiDe Vries, MattanjahPletschke, BrettDe Wit, MarynaPlíhalová, LucieDe Oliveira, Ronaldo NascimentoPliss, Artem M.De, RanjitPlochberger, BirgitDeacon, GlenPłotka-Wasylka, JustynaDeagostino, AnnamariaPlotniece, AivaDean, FrankPluskal, TomášDeazevedo, EduardoPluta, RyszardDeb, SubrataPluth, Michael D.Debnath, AsimPlyushch, ArtyomDębowski, MarcinPobelov, IlyaDec, JanPochard, IsabelleDecurtins, SilvioPocheć, EwaDefant, AndreaPochet, SylvieDegennaro, LeonardoPocurull, EvaDegiacomi, Matteo T.Poddelsky, AndreyDegl’innocenti, DonatellaPodkościelna, BeataDegola, FrancescaPodlewska, SabinaDehaen, WimPodlipnik, ČrtomirDehelean, CristinaPodolska, GrazynaDei, SilviaPodsedek, AnnaDeigner, Hans-PeterPodsiadły, RadosławDeja, StanislawPodvorica, FetahDel Caro, AlessandraPoeggeler, BurkhardDel Castillo-Santaella, TeresaPoetsch, AnsgarDel Fattore, AndreaPogacnik, LeaDel Pozo Bayón, Maria A.Poggi, AlessandroDel Río Celestino, MercedesPoggio, ClaudioDel Rio, GabrielPogorzelec-Glaser, K.Del Vas, MarianaPohl, Carolina H.Delaglio, FrankPohl, EhmkeDelaGrecca, MarinaPohl, PawelDelattre, CédricPohl, SebastianDelaude, LionelPohl, Sebastian Öther-GeeDelbarre-Ladrat, ChristinePohlkamp, TheresaDelfino, Domenico V.Pohlmann, AdrianaDelft, FlorisPoiana, MarcoDelgado, Daniel RicardoPoiana, Mariana-AtenaDelgado, Juan LuisPoinsot, VerenaDelgado, Sergio NogalesPóka, RóbertDelgado-Aguilar, MarcPoklar Ulrih, NatašaDelgado-Andrade, CristinaPokrywka, AndrzejDell’Agli, MarioPolák, BeataDell’Anna, Maria MichelaPolak-Berecka, MagdalenaDella Ca’, NicolaPoláková, MonikaDella Sala, PaoloPolakowski, Nicholas J.Dellafiora, LucaPolanski, MarekDellaGreca, MarinaPolčic, PeterDelle Piane, MassimoPoletto, MatheusDelogu, GiovannaPoli, AlessandroDeLong, Robert K.Politeo, OliveraDelsuc, Marc-AndréPolitis, IoannisDembinski, RomanPolito, LauraDembska, Anna RenataPollari, FrancescoDemchuk, Oleg M.Pollastro, StefaniaDeme, PragneyaPolosa, RiccardoDemeunynck, MartinePolverino, PierpaoloDemey, HaryPolyak, BorisDemir, FatihPolyakov, NikolayDemo, PavelPolz-Dacewicz, MałgorzataDemonacos, ConstantinosPoma, PaolaDemopoulos, Vassilis J.Pomastowski, PawełDeng, QiliangPomerantz, WilliamDeng, WenPomin, VitorDeng, YongmingPon Velayutham, Anandh BabuDenisow, BozenaPonce-Vargas, MiguelDenkov, NikolaiPonczek, Michał B.Denkova, AntoniaPonik, SuzanneDenny, BillPonnandy, PrabhuDent, MajaPons, JosefinaDeo, PermalPonti, AlessandroDeo, Randhir P.Pontiki, EleniDeodato, DavidePontrelli, PaolaDeora, GirdharPonzoni, IgnacioDepciuch, JoannaPoon, GregoryDerda, RatmirPop, AncaDerewiński, MirosławPop, Carmen RodicaDergunov, SergeyPop, CristinaDeriu, Marco AgostinoPop, LaurentiuDerkatch, SvetlanaPop, Tudor LucianDerkowska-Zielinska, BeataPopa, Daniela-SavetaDernovics, MihalyPopa, LacramioaraDerosa, JosephPopa, MarcelDerreumaux, PhilippePopa, Mona ElenaDerry, PaulPopa-Wagner, AurelDeryabina, Yu I.Popescu, RadianDesai, DhimantPopescu, VasilicaDesgranges, LionelPopgeorgiev, NikolayDeshpande, ShayuPop-Georgievski, OgnenDeslandes, EricPoplawski, TomaszDesmaële, DidierPopov, Alexey A.Desmarets, ChristophePopov, AntonDessbesell, LuanaPopova, Milena P.Dessolin, JeanPopović Đorđević, JelenaDestainville, NicolasPopovich, DavidDetsi, AnastasiaPorat, Ze’evDettin, MonicaPorcelli, FernandoDettmer, UlfPoręba, MarcinDettori, Maria LuisaPořízka, PavelDeutsch, AlexanderPorro, ChiaraDevasurendra, AmilaPortilla, JaimeDevaurs, DidierPortillo-Estrada, MiguelDevesa, JesúsPorto, André Luiz MeleiroDevi, LakshmiPortugal, Camila C.Devillers, JamesPoŝa, MihaljDevkota, Hari PrasadPoso, AnttiDey, Amit KumarPospisil, JiriDhanasekaran, MuraliPospisil, TomasDhasarathy, ArchanaPossik, EliteDhasmana, AnupamPotaczek, Daniel P.Di Bella, SantoPotaniec, BartłomiejDi Bussolo, ValeriaPotapov, AndreiDi Cerbo, AlessandroPotgieter, Martin JohannesDi Cesare Mannelli, LorenzoPothupitiya, JinalDi Chenna, PabloPotopnyk, MykhayloDi Giacomo, SilviaPoudel, Tej NarayanDi Giallonardo, FrancescaPouli, NicoleDi Gioia, Maria LuisaPouranian, M. RezaDi Giosia, MatteoPourbaghi Masouleh, MiladDi Girolamo, RoccoPournik, MaysamDi Liegro, ItaliaPous Rodríguez, NarcísDi Maro, AntimoPoveda Larrosa, Jose AntonioDi Martino, PieraPowers, EvanDi Mattia, Carla DanielaPowers, RobertDi Mauro, MariaPoynter, Matthew E.Di Micco, SimonePozio, AlfonsoDi Natale, ConcettaPozzi, CeciliaDi Nunzio, FrancescaPozzi, GianlucaDi Paola, RosannaPrabakaran, MayakrishnanDi Pasqua, Laura GiuseppinaPrabhu, VivekDi Pietro, SebastianoPracharova, JitkaDi Salle, AnnaPradhan, ShantanuDi Sanzo, RosaPradoMassarioli, AdnaDi Sarli, ValeriaPramanik, ArindamDi Sotto, AntonellaPrandi, CristinaDi Stefano, Stefano Pranovich, AndreyDi Zazzo, ErikaPraplan, Arnaud P.Di, RongPrasad, RamDiaba, FaïzaPrashar, SanjivDiaconeasa, ZoritaPrassl, RuthDiamantopoulos, GeorgiosPrat, MariaDiana, PatriziaPratesi, AlessandroDiana, RositaPratsinis, HarrisDias, Fábio De SouzaPredoi, DanielaDias, Maria CelestePreiner, DarkoDias, Maria InesPreite, MarceloDias-Junior, Carlos A.Prell, JamesDiaz Blanco, Manuel JesúsPremasiri, RanjithDiaz, ElenaPreston, DanDiaz, FernandoPretorius, SakkieDiaz, Sebastian AndresPretsch, ThorstenDiaz-maroto Hidalgo, María ConsueloPrévost, SébastienDiaz-Marrero, Ana R.Prhashanna, AmmuDíaz-Ortiz, ÁngelPricl, SabrinaDiban, NazelyPrieto Garcia, JoseDickens, AlexPrieto, ElenaDickert, Franz L.Prieto, IsabelDictor, Marie-ChristinePrieto, JoseDiederich, MarcPrieto, MiguelDiego, La MendolaPrieto, Miguel ÁngelDíez, DavidPrieto-Garcia, Jose M.Dijkstra, BaukePrieto-Lloret, JesusDikalov, SergeyPrijić, AnetaDileep, VishnuPrimožič, InesDiliën, HannePrisic, SladjanaDillon, HeatherPrisinzano, ThomasDima, CristianPritzker, KennethDimakis, NicholasPrivette Vinnedge, LisaDimakopoulos, YannisPrlić Kardum, JasnaDimandja, Jean-MarieProbert, ChrisDimarogona, MariaProchazka, MarekDimesso, LucangeloProchor, PiotrDimmock, Jonathan R.Procopio, Jesús R.Dimos, KonstantinosProdanovic, RadivojeDinache, AndraProestos, CharalamposDinadayalane, TandabanyProfire, LenutaDing, JianxunProietti, NoemiDing, ZhaoyangProkai, LaszloDingley, Andrew J.Prokop, IzabelaDini, IreneProkopov, TsvetkoDini, LucianaProrokova, Natalia PetrovnaDinic, JelenaProsa, MarioDinică, RodicaProsen, HelenaDinica, Rodica-MihaelaProtti, MicheleDinis, Maria Alzira PimentaProulx, CarolineDinischiotu, AncaProulx, StephanieDinis-Oliveira, Ricardo JorgeProvenzani, AlessandroDinos, GeorgiosProvost, PatrickDinu-Pirvu, Cristina-ElenaPrudêncio, MiguelDiogo, PatriciaPruna, Alina IulianaDiomede, FrancescaPruneanu, Stela-MariaDionisio, GiuseppePrusik, KrystianDionysopoulos, DimitriosPruvost, AlainDippong, ThomasPrywer, JolantaDireito, RosaPrzybył, JarosławDiring, StephanePrzybyłek, MaciejDissanayaka, WarunaPrzybylski, PiotrDivan, RaluPrzybylski, WiesławDiVerdi, JosephPsomas, GeorgeDixon, DavidPsurski, MateuszDixon, David A.Psycharis, VassilisDixon, NicholasPtaszek, AnnaDjavaheri-Mergny, MojganPtaszek, MarcinDjuran, Miloš I.Pu, XiaopingDludla, Phiwayinkosi V.Pucci, AndreaDługaszewska, JolantaPucek, AgataDłuska, EwaPuchades-Carrasco, LeonorDmochowski, IvanPuchalka, RadoslawDo Carmo, Sergio J. C.Pucheault, MathieuDobchev, Dimitar A.Puchkova, Ludmila V.Dobosz, RobertPuchta, RalphDobrikova, Anelia G.Puertas-Mejía, Miguel A.Dobrovolskaia, MarinaPugajeva, IvetaDobrowolski, PiotrPuiatti, MarceloDobrzynski, PiotrPulido, Olga MDobslaw, DanielPulito, ClaudioDocimo, TeresaPullano, Salvatore A.Doda, Sai ReddyPulvirenti, AlfredoDodero, VeronicaPum, DietmarDodi, GianinaPunta, CarloDogra, PranayPurcell, AnthonyDoh, Kyung-OhPurgatorio, RosaDohanosova, JanaPuri, AnuDohi, ToshifumiPurrello, RobertoDolashka, PavlinaPurse, Byron W.Dolegowska, BarbaraPurves, RandyDolezal, MartinPusch, StefanDolganov, Pavel VladimirovichPüschel, Gerhard P.Doligalska, MariaPushkar, YuliaDołowy, MałgorzataPuszka, AndrzejDolzhenko, Anton V.Puszkarewicz, AlicjaDołżonek, JoannaPutnam, William C. Domagała, MałgorzataPutrinš, MartaDomb, AviPuttreddy, RakeshDombrowski, YvonnePutz, MihaiDoménech-Carbó, AntonioPyka, AlinaDomin, HelenaPyka-Pająk, AlinaDomingues, Fernanda Da ConceiçãoPylypchuk, Ievgen V.Domingues, Sergio H.Pyne, StephenDomingues, Valentina F.Pyun, Jae-CheolDomínguez-Álvarez, EnriquePyzik, MichalDomínguez-Perles, RaúlQazi, SohailDomínguez-Ramírez, Julio LeninQi, HangDominguez-Robles, JuanQi, JiDominici, FrancoQi, YanfeiDominikowska, JustynaQi, ZhouDomitrović, RobertQian, JinDonadel, GiuliaQian, Jin-yuanDonadu, Matthew GavinoQian, MingxingDonaire, LiviaQian, ZhaoDonaldson, WilliamQiao, MingyuDonato, PaolaQiao, QiquanDonato, RosarioQiao, YuDonato, Rosario FrancescoQiaobin, HuDong, AlideertuQin, DajunDong, BoQin, DonghuanDong, GuangnengQin, SanboDong, HaiQin, XiaoliDong, QiuchenQin, YuDong, RenhaoQin, ZhihuiDong, XinglongQing, Lin-senDong, YiQiu, MinghuaDong, YueshengQiu, QiwenDonizetti, AldoQu, LingboDonnelly, SheilaQu, XiaozhongDonninger, HowardQuadrelli, PaoloDonnini, SandraQuan, GuilanDonohoe, Bryon S.Quax, PaulDonzelli, ElisabettaQueiroz, Maria JoãoDoody, Karen M.Queneau, YvesDorado, RubenQuesada, ErnestoDormán, GyörgyQuesne, MatthewDorotkiewicz-Jach, AgataQuevedo, Ivan R.Dos Reis, Paulo Nobre BalbisQuevedo, RobertoDos Santos Lima, MarcosQuijada-Garrido, IsabelDos Santos, António MoreiraQuijada-Maldonado, EstebanDos Santos, CatarinaQuiles Morales, Jose L.Dos Santos, Demetrio JacksonQuilez, JoseDos Santos, José Cleiton SousaQuiliano, MiguelDos Santos, Lucilene DelazariQuinet, PascalDosche, CarstenQuinn, Mark T.Doseff, Andrea I.Quintard, AdrienDOSOKY, NOURAQuintás, GuillermoDotsikas, YannisQuintas, Luis Eduardo M.Doucet, JeanQuintieri, LauraDoudin, KhalidQuinto, Emiliano JoséDouglas, TimothyQuinto, MaurizioDouroumis, DennisQuirino, Joselito P.Doušová, BarboraQuiroga, DiegoDovinova, ImaQuodbach, JulianDowling, JenniferQyyum, Muhammad AbdulDrabik, AnnaR. El-Seedi, HeshamDrabińska, NataliaRaabe, GabrieleDrabowicz, JozefRaak, NorbertDračínský, MartinRaasmaja, AtsoDracopoulos, VassiliosRabanal Ruiz, YoanaDragos, HorvathRabbani, GulamDragostin, Oana MariaRabilloud, FranckDragsted, LarsRabu, PierreDrasar, Pavel B.Racané, LivioDrescher, SimonRace, MarcoDreschers, StephanRachek, Lyudmila I.Dressel, MartinRachwalski, MichałDrevinskas, TomasRacoviceanu, RoxanaDrew, MichaelRácz, AnitaDrewniak, SabinaRadaram, BhaskerDrgan, ViktorRadchenko, Eugene V.Drijfhout, FalkoRadenkovic, MiroslavDrillet, Jean-FrançoisRadenković, SlavkoDrochioiu, GabiRadhakrishnan, Ammu K.Drolet, MarcRadic, ZoranDrouet, ChristianRadić, ZoranDrougard, AnneRadisavljevic, ZivDrozd, MarekRádis-Baptista, GandhiDruzbicki, KacperRadko, LidiaDrużyńska, BeataRadošević-Stašić, BiserkaDrzeżdżon, JoannaRadu, Gabriel LucianDu Plessis, Heinrich W.Radulescu, CristianaDu, ChengranRaduly, Lajos ZsoltDu, DeguoRadušienė, JolitaDu, GuodongRadwan, MohamedDu, MingRae, ColinDu, Zhen-XiaRaffa, DemetrioDu, ZhifengRafieenia, RaziehDuarte, Ana Rita C.Rafińska, KatarzynaDuarte, FátimaRafiński, ZbigniewDuarte, Filomena L.Ragab, KhaledDuarte, Iola F.Raganati, FrancescaDuarte, Maria CristinaRagauskas, ArthurDuarte, NoeliaRagg, EnzioDuatti, AdrianoRaghavan, ShreyaDubiel, WolfgangRagino, Yuliya I.Dubrova, YuriRagnini-Wilson, AntonellaDuburs, GunarsRagno, GaetanoDuchet-Rumeau, JannickRagno, RinoDucho, ChristianRagucci, SaraDuda, Marcel MateiRagusa, AndreaDudás, ZoltánRagusa, SalvatoreDudek, KatarzynaRaharivelomanana, PhilaDudev, TodorRahier, HubertDudhipala, NarendarRahimi, FaridDudina, DinaRahimi, ParvanehDudyński, MarekRahimi-Gorji, MohammadDuefer, MartinaRahman, ArmanDuggan, Brendan M.Rahman, Ashiqur X.Dulama, Ioana-DanielaRahman, AzizurDulf, FranciscRahman, FaizDuliu, OctavianRahman, M. AzizurDulova, NiinaRahman, Md ToufiqurDuma, LuminitaRahme, KamilDumas, JeraldRai, AnkitDumbrava, AncaRailean-Plugaru, VioricaDumitriu, Raluca-PetronelaRaimondi, LauraDumitru, GabrielaRaimondi, LaviniaDumont, JosephRaimondi, Maria ValeriaDunderdale, Gary J.Raimondo, DomenicoDunphy, Michael J.Raimundo, JoanaDuodu, Kwaku G.Raimundo, NunoDuplay, JoëlleRaina, DeepakDupureur, Cynthia M.Raina, KomalDurán, Alexandra GarcíaRainer, AlbertoDuran, CristhianRainey, JanDurán-Peña, María JesúsRais, RanaDuranti, AndreaRajauria, GauravDurazzo, AlessandraRajendiran, KarthikrajDurazzo, MarilenaRajić, ZrinkaDurgo, KsenijaRajkovic, IvanĐurović, SašaRajnak, MichalDusny, ChristianRajnarayanan, RajendramDussault, PatrickRaju, RiteshDutartre, PatrickRakass, SouadDutot, MélodyRakhmatullin, AydarDutta, AdityaRakitin, Oleg A.Dutta, KingshukRalston, EmilyDutu, LigiaRama, Ana RosaDuval, Raphaël E.Ramadan, SherifDuval, RomainRamadoss, AnanthakumarDux, MáriaRamaiahgari, Sreenivasa C.Duzgunes, NejatRamakrishnan, SayanthanDvoranova, DanaRamalho, Maria JoãoDwamena, AmosRamalho-Santos, JoaoDworatzek, ElkeRamalingam, NagendranDwyer-Nield, Lori D.Ramanavicius, ArunasDydio, PawełRamani, KomalDymińska, LucynaRamasamy, ElamparuthiDyszkiewicz-Konwińska, MartaRamasamy, ThiruganeshDzade, NelsonRamil, CarloDžeja, PetrasRamírez, DavidDziadas, Mariusz TomaszRamírez, MartínDziegiel, PiotrRamirez-Arcos, SandraDziekońska-Kubczak, UrszulaRamirez-Gonzalez, GustavoDziendzikowska, KatarzynaRamis, GianguidoDzierwa, AndrzejRammohan, AluruDziki, DariuszRamont, LaurentDzikuć, MaciejRamos, CatarinaDzimira, StanisławRamos, M. TeresaDziurka, MichalRamos, Marco A.Dzolic, ZoranRamos, Ricardo MartinsDzuba, Sergei AndreevichRamos, Rui MiguelDżugan, MałgorzataRamos, SoniaDzyuba, SergeiRamos-Organillo, ÁngelEbani, Valentina VirginiaRamos-Ortiz, GabrielEbrahim, WeaamRamos-Vivas, JoséEbrahimi Warkiani, MajidRampe, ElizabEthEbrahimi, AminRampias, TheodorosEchevarria, AureaRan, ChongzhaoEcheverria, FernandoRan, YanEckl, KatjaRanadheera, SenakaEda, ShigetoshiRanc, VáclavEddy, Matthew T.Rancan, MarzioEderson Rossi, AbaideRandazzo, Giuseppe MarcoEdin, HansRandazzo, WalterEdirisinghe, MohanRando, DanielaEdreira, Martin M.Ranjan, AmareshEdwards, CharlesRanjan, AtulEdwards, ChristineRanzato, EliaEdwards, Dylan R.Rao, AshitEdwards, RobertRao, AshutoshEffantin, GrégoryRapa, MattiaEfremenko, ElenaRapeanu, GabrielaEgami, HiromichiRâpeanu, GabrielaEgawa, YuyaRapone, BiagioEggleston, IanRapposelli, SimonaEgito, Eryvaldo Socrates Tabosa DoRaskovalov, AntonEgorin, AndreiRaspanti, MarioEgorova, KseniaRassias, GerasimosEhlers, PeterRastija, VesnaEhm, ChristianRastrelli, LucaEhrlich, HermannRasulev, BakhtiyorEhrmann, AndreaRatajewski, MarcinEhtezazi, TourajRatcliffe, NormanEid, NabilRatcliffe, Richard GeorgeEidenberger, ThomasRateb, MostafaEiroa, José LuisRather, IrfanEisenreich, WolfgangRathinasabapathy, AnandharajanEjidike, Ikechukwu P.Rathore, Atul S.Ekaterina, BartashevichRatke, LorenzEkielski, AdamRatnakumar, Bugga V.Ekino, KeisukeRattenholl, AnkeEklund, Patrik C.Raucci, UmbertoEl Badawy, Amro M.Raudino, AntonioEl Ghoch, MarwanRaugei, GiovanniEl Khalloufi, FatimaRaúl, Avila-SosaEl Rawas, RanaRault, FrancoisEl-Aarag, Bishoy Y.A.Ravanidis, StylianosElaissari, AbdelhamidRavi, Rama S.El-Amri, ChahrazadeRavin, Nikolai V.Elansary, Hosam O.Ravula, SudhirEl-Bacha, TatianaRawel, HarshadraiEl-Dars, FaridaRawson, JeremyEldeeb, MohamedRay, AsimEldridge, SandyRay, ParthaEleftheriadis, TheodorosRayan, AnwarEleftheriou, Eleftherios P.Raynie, DouglasElena-Herrmann, BénédicteRaza, AunElfalleh, WalidRazo, RodrigoEliav, EphraimRazorenov, Dmitry YurievitchEliseeva, Svetlana V.Re, SuyongElizalde, Luis E.Rea, IlariaEljaafari, AssiaRéal, FlorentElkabets, MosheReany, OferEller, FredRébé, CédricEllett, FelixReber, ChristianElliott, Paul I.P.Rebollo-Hernanz, MiguelEllison, MichaelRecabarren Gajardo, GonzaloEl-Moghazy, AhmedRecek, NinaElmquist, Randolph E.Redan, BenjaminEl-Nabulsi, Rami AhmadReddy, M. V.El-Safty, SherifReddy, PriyankaElsebai, MahmoudReddy, SakamuriEl-Seedi, HeshamRedell, JohnElshafie, Hazem S.Redondo, Pedro CosmeElshamy, AbdelsamedRedshaw, CarlElsner, MatthiasRee, MoonhorEmberson, DavidReeh, PeterEmwas, Abdul-HamidReekie, Tristan A.Enache, Mădălin-IancuRees, D. Andrew S.Enchev, VenelinReetz, Manfred T.Encinar, Jorge RuizReeve, VivienneEndo, TatsuroRegazzoni, Luca GiovanniEndoh, TamakiRege, AmeyaEnfedaque, AlejandroRegiec, AndrzejEngel, W. DieterRegmi, BishnuEngelmann, PeterReguero, MarEnko, DietmarRehder, DieterEnkvist, ErkiRehman, Junaid UrEnman, JosefineRehmann, HolgerEnyedy, Éva AnnaReid, Russell C.Eo, Soo HyungReid, ScottEparvier, VéroniqueReimann, Regina R.Eppard, ElisabethReinach, Peter S.Era, BenedettaReis, CatarinaEras, JordiReis, Catarina PintoErb, WilliamReis, CristianoErben, MauricioReis, Glaydson Simoes DosEremeyev, VictorReiser, OliverEri, S. SrivatsanReiss, Lucinda V.Erickson, Jeffrey S.Rejah, ShahiErickson, TimothyRelat, JoanaEriksson, KristinaRemedios Serrano, DoloresEritja, RamonRemenyik, JuditErjavec, Gordana NedicRen, BaozengErnesto, Cortés-CastellRen, HaoErnesto-Casanova, OscarRen, QinlongErrandonea, DanielRen, ShijieErsilia, AlexaRen, YanrongEsatbeyolgu, TubaRen, YulinEscalante, JaimeRenard, Pierre-YvesEscames, GermaineRenaud, Jean-LucEscárcega Bobadilla, Martha VerónicaRenaud, JustinEscargueil, AlexandreRenault, NicolasEscario, JoséRenčiuk, DanielEscobedo, GalileoRencoret, JorgeEscola, José MaríaRendle, Phillip M.Escudé, ChristopheRengasamy, KannanEscudero, CarlosRenò, FilippoEscudero, DanielRentschler, EvaEscudero, OlgaRenukuntla, JwalaEseev, MaratRenzo, VannaEser, Bekir EnginReščič, JurijEskin, MichaelRescifina, AntonioEsmaeely Neisiany, RasoulRescigno, AntonioEspada-Bellido, EstrellaResconi, Virginia CeliaEspartero, José L.Resendiz, MarinoEspiña, BegoñaReshetilov, Anatoly N.Espinach, Francesc XavierRestivo, Francesco M.Espindola, Laila SalmenReuter, KlausEspínola, FranciscoRevelou, Panagiota-KyriakiEspinós, CarmenRevilla, EugenioEspinosa, EduardoRevnic, CorneliaEspita, PaulaRevuelta, JuliaEsposito, Emilio XavierRey, FelisaEsposito, GermanaReyes, ArchieEsposito, VeronicaReyes, FernandoEspuelas, SocorroReyes-Chilpa, RicardoEsquifino, Ana IsabelReyes-Ortega, FelisaEsquivel, BaldomeroReynisson, JóhannesEsquivel, KarenReza, Khan MamunEstay, HumbertoŘezáčová, MartinaEstelrich, JoanRezaei, NimaEsteve-Romero, JosepRezania, ShahabaldinEsteves, EduardoŘezanka, MichalEstévez Cabanas, Ramón J.Reznikov, Leah R.Estevez, AnaRezvani, KhosrowEstévez, JorgeRezzani, RitaEstevinho, Leticia M.Rhazi, LarbiEtienne, ThibaudRhee, Ki-JongEtinski, MihajloRhee, Young HoEto, BrunoRho, Jung-RaeEttayapuram Ramaprasad, Azhagiya SingamRial, RaquelEu, PeterRibaudo, GiovanniEudald, CasalsRibechini, ErikaEugenio, ApreaRibeiro Macedo, SandraEugster, PhilippeRibeiro, ArturEvanno, LaurentRibeiro, ClaudiaEvans, ChristopherRibeiro, HelenaEvans, StephenRibeiro, Luís FilipeEvans, WilliamRibeiro, MiguelEvaristo, JaivimeRibeiro, ThierryEvergren, EmmaRicardo Da Silva, JorgeEwald, AndreaRicardo, FernandoEyer, LuděkRicardo, GobatoEyres, GrahamRicardo-da-Silva, JorgeEzekiel, UthayashankerRiccardi, ClaudiaEzhov, MaratRicchi, MatteoEzquerra-Brauer, Josafat-MarinaRicci, AriannaF. G. Dias, FernandaRicci, Pier CarloFábián, IstvánRiccio, AngeloFabian, ZsoltRiccò, RaffaeleFabiani, RobertoRice, Cynthia AnnFabianowski, WojciechRicelli, AlessandraFabio, MarmottiniRichards, Dylan JackFabiszewska, AgataRichardson, AlanFabroni, SimonaRichardson, Rudy J.Facchetti, GiorgioRichardson, SimonFaccio, GretaRichert, RankoFadda, AngelaRichter, KatrinFages, JacquesRichter, PeterFaia, Arlete MendesRichter, ShacharFairfull-Smith, KathrynRichtera, LukášFaissner, SimonRidruejo, ÁlvaroFakis, MihalisRiedel, StefanieFalcão, Soraia I.Rigalli, Juan PabloFalciani, ChiaraRigamonti, LucaFalcieri, ElisabettaRigano, DanielaFalcone, Pasquale MassimilianoRighetti, LauraFalconer, Ian R.Rigoni, FedericaFalconer, RobertRijo, PatríciaFaleiro, Maria LeonorRimbach, GeraldFalqué López, ElenaRimpelova, SilvieFalsini, SaraRinaldi, FedericaFan, DeliangRincon Joya, MiryamFan, JizhouRinnerthaler, MarkFan, LiangRintoul, IgnacioFan, QiaohuiRío Segade, SusanaFan, XugeRiobé, FrançoisFan, ZhijinRios, RamonFanali, SalvatoreRiou, LaurentFang, Guor-ChengRipolles, Teresa S.Fang, LiangRisbridger, GailFang, MarongRiscoe, MikeFang, MingxiRisinger, April L.Fang, QingmingRispoli, ConcettaFang, Ronnie H.Ristic, RenataFang, YiRitter, HelmutFanizzi, Francesco PaoloRitz, UlrikeFantacuzzi, MarialuigiaRivadeneyra, AlmudenaFapyane, DebyRivas, CarmenFarabegoli, FulviaRivas, FatimaFarantos, Stavros C.Rivera De La Rosa, JavierFaraone Mennella, Maria RosariaRivera, Víctor M.Farcas, Anca CorinaRivera-Monroy, Zuly JennyFarcasanu, Ileana C.Rivero Cruz, José FaustoFarci, DomenicaRivero-Pérez, M. DoloresFare, SilviaRivilla, RafaelFarias-Pereira, RenalisonRizvi, Syed A. A.Fariba, FathiRizzi, CorradoFarina, NicholasRizzi, LauraFarinha, CarlosRizzi, MenicoFarjas, JordiRizzo, CarlaFarkas, AttilaRizzo, FabioFarkas, EtelkaRizzolio, FlavioFarkaš, PavolRizzuti, BrunoFarmer, PatrickRo, Hyeon-SuFarris, StefanoRoa Rovira, Joan JosepFarshbaf, SepidehRobaszkiewicz, AgnieszkaFarzaneh, VahidRobert Sosnowski, TomaszFasciana, TeresaRobert, PazFatás, EnriqueRoberto Da Silva, EdsonFatima, TahiraRoberts, Arthur G.Fattuoni, ClaudiaRoberts, Stephen K.Fauconnier, Marie-LaureRoberts, TaraFaustino, M. Amparo F.Robertson, Mark J.Fausto, RuiRobeyns, KoenFavero, LucillaRobien, WolfgangFavreau, Peter F.Robiette, RaphaelFavre-Réguillon, AlainRobinson, SeriFazio, AlessiaRobledo Quintos, Norma RFazio, BarbaraRocca, RobertaFazio, EnzaRocchetti, GabrieleFazzi, DanieleRocchigiani, LucaFeás, XesúsRocha, CláudiaFecka, IzabelaRocha, Djenisa H. A.Feczkó, TivadarRocha, HugoFederico, StephanieRochais, ChristopheFedin, VladimirRocha-Martin, JavierFedoreyev, SergeyRochani, AnkitFedosov, SergeyRoche, EnriqueFedotov, Petr S.Roche, ManonFedriani, Luis EsquiviasRoche, MichelleFeintuch, AkivaRochfort, SimoneFeis, AlessandroRocio-Bautista, PriscillaFekete, AgnesRockenfeller, PatrickFelczak, Krzysztof Z.Roda, GabriellaFeldblyum, Jeremy I.Rode, Tone MariFeldman, SteveRodembusch, FabianoFelgueiras, HelenaRodembusch, Fabiano S.Felipe-Sotelo, MonicaRodger, AlisonFeliu, LidiaRodilla, VicentFelix, Anderson AndréRodolfo, CarloFélix, LuisRodrigo, LuisFélix, Luís M.Rodrigo, Sara MoralesFeliz, MartaRodrigues, AlírioFelpin, François-XavierRodrigues, Clóves G.Feng, ChaohuiRodrigues, FranciscaFeng, Chao-HuiRodrigues, JoãoFeng, Chia-HsienRodrigues, Luiz FredericoFeng, LijuanRodrigues, MárcioFeng, Sheng-WeiRodríguez Díaz, Juan CarlosFeng, ShuanglongRodríguez González, ÁlvaroFeng, Wei-shengRodríguez González, VicenteFeng, XiamingRodríguez López, María IsabelFeng, Yong-LaiRodriguez Rojo, SorayaFenning, DavidRodríguez Romero, AdelaFenyvesi, EvaRodríguez Villanueva, JavierFerbinteanu, MarilenaRodriguez, GuillermoFercher, ChristianRodriguez, Hortensia M.Ferdov, StanislavRodriguez, JaimeFeregrino-Pérez, Ana A.Rodríguez, José AntonioFerenc, PajorRodríguez, LauraFeresin, GabrielaRodríguez, MarioFeresin, Rafaela G.Rodríguez, Ricardo ReyesFerey, LudivineRodriguez-Couto, SusanaFermo, PaolaRodríguez-Flores, M. ShantalFernadez-Bolanos, José G.Rodríguez-López, Carlos E.Fernandes, AlexandraRodriguez-Lorenzo, LauraFernandes, AntonyRodríguez-Roque, María JanethFernandes, Carla Sofia GarciaRodriguez-Sinovas, AntonioFernandes, Elizabeth S.Rodríguez-Yoldi, María JesúsFernandes, JolynRodriquez, HectorFernandes, José OliveiraRodriquez, ManuelaFernandes, Maria HelenaRodziewicz-Motowidło, SylwiaFernandes, Rosa Cristina SimoesRoedel, FranzFernandes, Tiago A.Roex, ErwinFernández Barbero, GerardoRofouie, PardisFernández Ramos, María DoloresRoger, Jean MichelFernandez, AntonioRoger, JulienFernandez, BelenRoglans, AnnaFernandez, CarlosRogobete, Alexandru FlorinFernández, InmaculadaRogozhin, EugeneFernández, Juan A.Roh, ChanghyunFernández, María JesúsRoh, JaroslavFernandez, Maria LuzRohanifar, AhmadFernandez, MercedesRohde, KyleFernandez, Pablo SebastianRohlena, JakubFernandez, Wadie MahauadRohn, SaschaFernández-Arévalo, MercedesRöhrig, UteFernandez-Garcia, JavierRoj, EdwardFernández-Gutiérrez, MarRojas, ClaudiaFernandez-Lafuente, RobertoRojas-Aguirre, YareliFernández-Lázaro, FernandoRojas-Hernandez, Rocío EstefaníaFernández-Lucas, JesúsRojo, Maria AngelesFernández-Moreira, VanesaRokavec, MatjazFernandez-Salas, Jose A.Rokosz, KrzysztofFernández-Soto, PedroRolfe, Barbara E.Fernando, BinoshaRolim, AdrianaFeroci, MartaRolle, LucaFerraboschi, PatriziaRollin, PatrickFerramosca, AlessandraRolo, JoanaFerrandez-Villena, ManuelRomán Guerrero, AngélicaFerrante, ClaudioRoman, CelianFerrante, FrancescoRomani, AndreaFerrante, Maria ImmacolataRoman-Junior, WalterFerrantelli, VincenzoRomano, GiovannaFerraris, DanaRomański, JarosławFerraro, VincenzaRomar, HenrikFerreira, Ana M.Roma-Rodrigues, CatarinaFerreira, AntónioRomeo, IsabellaFerreira, GuilhermeRomeo, RobertoFerreira, JOANARomero, AlejandroFerreira, Leonardo G.Romero, Marta R.Ferreira, Leonardo M.R.Romero, Rosenberg J.Ferreira, Luisa MariaRomero-González, RobertoFerreira, MarceloRomero-Muñiz, CarlosFerreira, NelsonRomero-Salguero, Francisco J.Ferreira, OlgaRomoli, OttaviaFerreira, Paula M.Ronca, AlfredoFerreira, Ricardo J.Roncada, PaolaFerreira, Sandra Regina SalvadorRončević, SandaFerreira, SusanaRonchetti, SimonaFerreiro, MartaRonconi, LucaFerreiro-González, MartaRondeau-Gagné, SimonFerrentino, GiovannaRondon, AurélieFerreon, JosephineRonquist, GunnarFerrer, BelenRonzan, MarilenaFerrer, EmiliaRoose, BartFerrer, GerardoRoosen-Runge, FelixFerrer-Gallego, RaulRoot, MartinFerreri, CarlaRoper, Stephen DFerri, NicolaRoppolo, IgnazioFerrieres, VincentRoque, AliciaFerrini, FrancescoRoques, JonathanFerrini, SilvanoRosa Da Silva, RobsonFerro, YveliseRosa, AntonellaFershtat, LeonidRosa, Arianna CarolinaFesta, CarmenRosa, MarcoFicai, AntonRosado, CatarinaFidalgo, AlexandraRosales, JuliaFidelibus, CorradoRosales-Hernández, Martha CeciliaFideline, Tchuenbou-MagaiaRoscini, LucaFiedler, HolgerRosenau, ThomasFiedot-Toboła, MartaRosenberg, ErwinFierascu, IrinaRosenberg, IvanFierro, José FernandoRosenberg, MosheFierro, VanessaRosenberry, TerroneFifere, AdrianRosenthal, KatrinFigaj, DonataRosenzweig, DerekFigueira, FlavioRosi, MarzioFigueiral, Maria HelenaRosicka-Kaczmarek, JustynaFigueiras, AnaRosini, ElenaFiguereido Neto, AntonioRosokha, Sergiy V.Figueroa Cardenas, Juan De DiosRoss, Ian LFijałkowski, KarolRoss, KirstinFik-Jaskółka, Marta A.Ross, Robert L.Filannino, PasqualeRoss, ThyerFilarowski, AleksanderRos-Santaella, Jose LuisFilella, XavierRossella, DoratiFilice, SimonaRossella, FrancescoFilip, AdrianaRossi, ClaudioFilip, JaroslavRossi, FilippoFilip, Lucian DragosRossi, FilomenaFilipeanu, CatalinRossi, GiancarloFilipova, IneseRossi, Jean-ChristopheFilippi, LucaRossi, MarcoFilippou, Panagiota S.Rossi, MiriamFilippov, AndreiRossi, SergioFilippov, Oleg A.Rostoker, GuyFimognari, CarmelaRoszak, SzczepanFinelli, CarmineRoszkowska, AnnaFini, PaolaRotaru, AndreiFiocco, DanielaRotblat, BarakFiore, Carlos E.Roterman, IrenaFioretti, BernardRoth, MichaelFiorica, CalogeroRotondo, ArchimedeFiorillo, LucaRotstein, Benjamin H.Fiorin, GiacomoRoubaud, AnneFiorucci, SebastienRoubelakis-Angelakis, Kalliopi A.Firlak, MelikeRouphael, YoussefFischer, Hartmut R.Roussis, Ioannis G.Fischer, HenrikeRout, BhimsenFischer, HolgerRovini, AmandineFischer, RolandRovisco, AnaFischer, WolfgangRovnak, JoelFischer-Fodor, EvaRow, Kyung HoFisher, CarlRowlands, Gareth J.Fisher, Jonathan S.Rowles, MatthewFisicaro, EmiliaRoy Chowdhury, SandipanFisslthaler, BeateRoy, AnuradhaFiszer, AgnieszkaRoy, DhruvajyotiFitta, MagdalenaRoy, DipankarFitter, JörgRoy, JugantaFitzpatrick, AnthonyRoy, RenèFiumara, GiacomoRóżalska, BarbaraFizeșan, IonelRóżalska, SylwiaFlamini, GuidoRozalski, MarcinFlanagan-Steet, HeatherRozès, NicolasFleet, GeorgeRozhon, WilfriedFletcher, MaryRožić, MirelaFleurat-Lessard, PaulRozwadowski, TomaszFlieger, JolantaRóżycki, BartoszFloerke, UlrichRóżyło, RenataFlorán, BenjamínRozza, ArianeFlores Johnson, Emmanuel AlejandroRuan, KeFlores, Ana IsabelRuaro, BarbaraFlores-Morales, VirginiaRubakhin, StanislavFlórez-Fernández, NoeliaRubčić, MirtaFlorian, HübnerRubino, Federico MariaFloris, BarbaraRubio Alonso, FaustoFlorowska, AnnaRubio Alonso, JuanFlythe, MichaelRubio-Martinez, JaimeFocaroli, StefanoRubis, BlazejFoley, Ann C.Rudat, JensFollink, BartRuddraraju, Kasi ViswanatharajuFologea, DanielRudić, SvemirFonseca, FilomenaRudick, JonathanFont, JoaquimRüdiger, StefanFontaine, VéroniqueRudiuk, SergiiFontana, AntonellaRudnicka, WiesławaFontana, ArielRudnóy, SzabolcsFontana, FedericoRudshteyn, BenjaminFontana, JuanRudzińska, MagdalenaFontana, SimonaRueda Ordóñez, Yesid JavierFontanals, NúriaRuegg, UrsFontanay, StéphaneRuelland, EricFontanini, DeboraRuffino, FrancescoFonte, PedroRuffolo, GabrieleFontés, MichelRuiu, LucaForbey, Jennifer S.Ruiz Téllez, TrinidadForero-Quintero, Linda S.Ruiz, MarioFormato, MarilenaRuiz-Angel, Maria JoséFormisano, PietroRuiz-Rubio, LeireFornai, FrancescoRuiz-Sánchez, EsaúFornarini, SimonettaRulíšek, LubomírFornstedt, TorgnyRuml, TomasForoozandeh, MohammadaliRumlová, MichaelaForte, ClaudiaRumyantseva, MarinaFortenberry, RyanRupasinghe, ThusithaForti, LucaRuperez, Francisco J.Forzato, CristinaRuphuy, GabrielaFossa, PaolaRuppert, RomainFoster, Leonard J.Rusciano, Maria RosariaFoster, Toshana LauriaRusike, ConstanceFoti, ClaudiaRusinek, RobertFoubelo, FranciscoRusmini, PaolaFouillaud, MireilleRusnati, MarcoFourmentin, SophieRussell, FraserFournier, BertrandRusso Krauss, IreneFousteris, ManolisRusso, AlessandraFracassetti, DanielaRusso, LauraFracasso, GiulioRusso, LuigiFracchiolla, Nicola StefanoRusso, MariateresaFraga, HugoRusso, MarinaFragai, MarcoRusso, Mario VincenzoFraga-López, FranciscoRusso, NinoFragopoulou, ElizabethRusso, PasqualeFraguas-Sánchez, Ana IsabelRusso, PietroFraile, AlbertoRusso, RobertaFranca, Eduardo F.Russo, RositaFranceschelli, SaraRusso, TeresaFranceschi, PieroRussu, WadeFrancesconi, M. GraziaRusu, DanielaFrancés-Monerris, A.Ruthstein, SharonFrancés-Soriano, LauraRutkowska, MałgorzataFranchina, Flavio AntonioRuysschaert, Jean-MarieFrancioso, AntonioRuzik, LenaFrancisco, Ana PaulaRuzza, ChiaraFrančišković-Bilinski, StanislavRyabchikova, Elena I.Franckhauser, SylvieRyabchun, AlexanderFranco, Camilo AndresRyadnov, MaxFranco, Carlos M.Ryan, AliFranco, ChrisRybak, KatarzynaFrancolini, IolandaRybarczyk, PiotrFrank, Craig L.Rybarczyk-Pirek, Agnieszka J.Frank, EvaRybczyńska-Tkaczyk, KamilaFrank, JanRybka, JakubFrank, Luiza AbrahãoRyde, UlfFrank, RenéRykowska, IwonaFrankowski, MarcinRyo, UshiodaFrański, RafałRyoo, SungweonFranssen, Maurice C. R.Ryu, BoMiFrantziskonis, George N.Ryu, Jae-HaFranzyk, HenrikRyu, Ji HyunFrapiccini, EmanuelaRyu, Seung YoonFrasca, LoredanaRywaniak, JoannaFratantonio, DeborahRyzhov, VictorFratini, FilippoRzelewska-Piekut, MartynaFratti, Rutilio A.Sa, Jeong-HoonFrazier, CharlesSaaby, LasseFreakley, SimonSaad, JamilFrecer, VladimirSaada, AnnFrédérick, RaphaëlSaba, SumbalFreitag, MarinaSabantina, LiliaFreitas De Freitas, LucasSa-Barreto, Livia L.Freitas, Vanessa MoraisSabaté, RaimonFreitas, Vera L. S.Sabater i Serra, RoserFrelon, SandrineSabatini, Jesse J.Frenking, GernotSabatini, StefanoFrense, DieterSabatino, DavidFrerich, Sulamith C.Sabatino, ManuelaFresco-Cala, Beatriz MaríaSabir, ShekhFreudenthal, Bret D.Sabiu, SaheedFrey, KathleenSaboe, PatrickFreyria, Francesca StefaniaSabol, IvanFrezza, ClaudioŠabovič, MišoFrezza, ElisaSabri, FirouzehFrič, JanSaccenti, EdoardoFriedrich, Holger B.Sacchetti, AlessandroFriesen, J. BrentSacco, RiccardoFrija, LuísSaccone, SalvatoreFrippiat, Jean-PolSachadyn-Król, MonikaFrisch, BenoîtSacher, EdwardFrisch, KimSachse, AlexanderFrittitta, LuciaSacks, GavinFrizzo, Clarissa P.Sackus, AlgirdasFrkanec, RužaSacré, Pierre-YvesFröde, Tânia SilviaSaczonek, Agnieszka OwczarczykFroeyen, MathySadeghi, SamanFrogley, MarkSadeghpour, AminFröhlich, EleonoreSadgrove, NicholasFroldi, GuglielminaSadhasivam, SudharsanFrolov, AndrejSadjadi, SeyedAbdolrezaFrolova, LiliyaSadovskaya, IrinaFrolova, Tatiana S.Sadowska, AnnaFrontera, AntonioSadowska, MonikaFrontera, PatriziaSadowska-Krępa, EwaFruchon, SéverineSaeb, Mohammad RezaFruit, CorinneSaeki, KumikoFruk, GoranSafa, Ahmad R.Fry, AndrewSafe, Stephen H.Frye, Stephen V.Šafratová, MarcelaFu, LiyeSagar, Robin P.Fu, PengSagnou, MarinaFu, TengfeiSagona, SimonaFu, XiaotingSah, HongKeeFu, XuSaha, Manik LalFucikova, AnnaSaha, RajibFuentes-Rivas, Rosa MaríaSaha, SubhasishFuglsang, Anja ThoeSahoo, SanjubalaFujihara, HisakoSahu, Ravi PFujihara, TetsuakiSaiano, FilippoFujii, HideakiSaiardi, AdolfoFujii, YoshiharuSaidaminov, MakhsudFujimori, KoSaielli, GiacomoFujimura, YoshinoriSaifutdinov, Bulat R.Fujioka-Kobayashi, MasakoSaikat, BalaFujisawa, KiyoshiSaiko, GuennadiFujisawa, TomotsumiSaikova, SvetlanaFujita, Ken-ichiSainlos, MatthieuFujita, MasakiSaint-Fort, RogerFujita, MikakoSaito, AkikoFujita, MitsuguSaito, KanFujita, MorifumiSaito, MasaichiFujita, WakakoSaito, SatoshiFujiwara, AkihikoSaito, ShinichiFujiwara, TomoyaSaito, YoshinoriFukase, KoichiSaitta, MarcoFukin, Georgy K.Sajeva, MaurizioFuliaş, Adriana VioletaSajnóg, AdamFulignati, SaraSakač, NikolaFuller, AmeliaSakai, NobuyukiFulvio, Pasquale FernandoSakai, RyuichiFumagalli, LauraSakamoto, Kathleen M.Fun, Lim YeeSakamoto, MasahiroFunar-Timofei, SimonaSakamoto, TakeshiFunazukuri, ToshitakaSakamuro, DaitokuFunes-Ardoiz, IgnacioSakar, MohanFurdui, BiancaSakata, MasahiroFurlan, AlessandroSakilam, SatishFurlani, FrancoSakkas, HerculesFurlani, MaurizioSakurai, KazumasaFurse, SamuelSakurai, TsuneakiFuruishi, TakayukiSalachna, PiotrFurukawa, TatsuhikoSalafranca Lázaro, Francisco J.Furusawa, HiroyukiSalahub, DennisFuruta, ToshiakiSaláková, AlenaFurutani, YutakaSalamanca, Constain H.Fusco, RobertaSalanţă, Liana ClaudiaFuse, ShinichiroSalas, CarlosFuso, FrancescoSałat, KingaFuu, SheuSalaün, FabienFuwa, HaruikoSalazar, Juan RodrigoFuxreiter, MonikaSaldanha, CarlotaGaan, SabyasachiSaleh, MahmoudGabano, ElisabettaSaleh-E-In, Md. MoshfekusGabbar, Hossam A.Salerno, LoredanaGabbia, DanielaSales, Rita De Cássia MendonçaGabr, MoustafaSalhab, HassanGabriele, BartoloŠalić, AnitaGabrielli, PaoloSalman, MootazGacesa, RankoSalmaso, VeronicaGaczynska, MariaSalmerón, IvanGad, Annica K. B.Salmon, EricGadipelli, SrinivasSalo-Ahen, OutiGadjanski, IvanaSalomone, AntonioGadomski, AdamSalonen, LauraGadzała-Kopciuch, RenataSalopek-Sondi, BrankaGadžurić, SlobodanSaltas, VassiliosGagaoua, MohammedSalto-Gonzalez, RafaelGagat, PrzemyslawSalvado, VictoriaGago, CustódiaSalvarani, NicolòGai, ChiaraSalvatori, RobertaGaidau, CarmenSalvatori, StefanoGailer, JürgenSalvetti, AnnaGain, Asit KumarSalvi, Anna M.Găină, Luiza IoanaSalvo, AndreaGaisberger, MartinSamanidou, VictoriaGajc-Wolska, JaninaSamardžić, JelenaGajdács, MárióSamarov, Artemiy A.Gajewicz-Skretna, AgnieszkaSambri, AndreaGal, Adrian FlorinŠamec, DunjaGal, EmeseSamek, OtaGala, Rikhav PSamhan-Arias, AlejandroGalabov, AngelSamková, EvaGalamba Correia, João DomingosSammalkorpi, MariaGalamboš, MichalSampaio, AnaGalán, Miguel LaderoSampath, ChethanGalanakis, CharisSamperi, FilippoGalanakis, Charis M.Sampson, Nicole S.Galanis, AlexisSamson, WillisGalasiti Kankanamalage, AnushkaSamsonov, SergeyGalasso, GiovanniSan Fabian, EmilioGałązka-Czarnecka, IlonaSan Miguel, VeronicaGalbács, GáborSanchez Galan, JavierGalderisi, UmbertoSánchez, GoarGaleazzi, RobertaSánchez, Jesus LozanoGales, LuísSanchez, Jose M.Galhano, AlexandraSanchez, RaquelGalic, AnteSánchez-Barba, Luis F.Galicia-García, TomásSánchez-Fidalgo, SusanaGalina, GenchevaSánchez-García, DavidGalinetto, PietroSánchez-Gómez, RosarioGalione, AntonySánchez-Martín, María JesúsGałkowska, DorotaSánchez-Monedero, MiguelGall Troselj, KoraljkaSanchez-Pastor, EnriqueGallagher, John F.Sanchez-Salas, JoseGallardo, EugéniaSánchez-Soto, Luis L.Gallardo, M. EstherSánchez-soto, PedroGallastegui, GorkaSánchez-Vergara, María ElenaGalletti, CamillaSancho-Garcia, Juan-CarlosGallo, MonicaSancineto, LucaGalloni, PierlucaSancini, GiulioGallorini, MarialuciaSandalio, Luisa MaríaGalperin, Michael Y.Sandeep, DaveGalstyan, AnzhelaSandhu, AmandeepGalus, SabinaSandhu, BhupinderGaluska, Sebastian P.Sandhu, Jagdeep K.Galuska, Sebastian PeterSandjo, Louis PergaudGalvão, Adelino M.Sandlers, YanaGálvez Buerba, Eva M.Sándor, KristyánGalvez, JorgeSándor, ZoltánGálvez, JulioSandra, BeaufortGalvosas, PetrikSandu, Andrei VictorGalzitskaya, Oxana V.Sang, YongmingGambardella, AlessandroSangiovanni, DavideGambari, RobertoSangiovanni, EnricoGamba-Sánchez, DiegoSani, RajeshGambino, GiorgioSanina, Nina M.Gambuti, AngelitaSanjeet, MehariyaGameiro, PaulaSanjeewa, Kalu Kapuge AsankaGamelas, JoséSanjurjo Sanchez, JorgeGamella Carballo, MariaSanjust, EnricoGamiño-Arroyo, ZeferinoSankaranarayanan, Nehru VijiGan, RenyouSan-Miguel, AdrianaGan, Ren-YouSanna, GavinoGan, SamuelSanna, NicoGanatsios, VassiliosSanner, Michel F.Ganazzoli, FabioSannino, FilomenaGancarz, RomanSanny, CharlesGandiglio, MartaSans, AlbertGandomkar, SomayyehSansbury, Brian E.Ganesan, A.Sansone, AnnaGanesan, PalanivelSansone, FrancescaGang, WeiSansone, FrancescoGangoli, Varun ShenoySantacruz Ortega, Hisila Del CarmenGanguly, AvishekSantagapita, PatricioGanguly, PritamSantamaria, PietroGanguly, SamitSantamaria, RitaGanji, RakeshSantamaria-Echart, ArantzazuGanser, ChristianSantana, Andrés GonzálezGao, BeiSantarem, NunoGao, BoyanSanthakumar, Abishek BommannanGao, ChengdeSanthoshkumar, PutturGao, GarySanti, ClaudioGao, HuileSantilio, AngelaGao, JinSantin, Silvana M. O.Gao, PeiyuanSantini, AntonelloGao, YongfengSantini, CarloGao, ZheSantisteban, MariaGarapati, NagasreeSantoro, FabrizioGarbar, ChristianSantos De Gois, JeffersonGarbayo, InésSantos Júnior, Aníbal De FreitasGarbers, ChristophSantos Mendes, Luis FelipeGarcía Fernández, José ManuelSantos, Carla S.García Gómez, SergioSantos, ClementinaGarcia Ispierto, IrinaSantos, Cleydson Breno R.Garcia, AntonioSantos, Eva M.Garcia, Coralia V.Santos, Maria AméliaGarcia, JordiSantos, Maria M. M.Garcia, María DoloresSantos, Raquel De Cássia DosGarcía, Marta MesíasSantos, Ronaldo G.Garcia, RosarioSantos, Sonia A.O.García-Alonso, Francisco JavierSantos, Susana S.García-Arredondo, AlejandroSantos, Willy GlenGarcia-Benett, AlfonsoSantos-Filho, Osvaldo AndradeGarcia-Bernabé, AbelSantovito, GianfrancoGarcia-Bosch, IsaacSantra, BiswajitGarcía-Conesa, María-TeresaSantucci, AnnalisaGarcia-Deibe, AnaSañudo-Barajas, Josefa AdrianaGarcía-Estañ, JoaquínSanz-Marco, AmparoGarcia-Fandino, RebecaSaponara, SimonaGarcía-Fresnadillo, DavidSarabia, FranciscoGarcia-Gallego, SandraSaraiva, NunoGarcía-García, Rosa MaríaSarandeses, Luis A.García-González, VíctorSarasini, FabrizioGarcía-Martínez, Jesús-MaríaSaravana, Periaswamy SivagnanamGarcía-Mena, JaimeSaravanakumar, KandasamyGarcia-Mina, Jose MariaSardone, RodolfoGarcia-Pardo, JavierSaretzki, GabrieleGarcía-Ramos, Juan CarlosŠarkanj, BojanGarcia-Reyes, Refugio B.Sarkar, Amit KumarGarcia-Sosa, AlfonsoSarkar, DhrubaGarcia-Vallve, SantiagoSarlis, NicholasGarcia-Verdugo, IgnacioSarmento-Ribeiro, Ana BelaGarcía-Viguera, CristinaSarno, FedericaGardikis, KonstantinosSartorelli, PatriciaGargiulo, ValentinaSasai, YasushiGarncarek, ZbigniewSasaki, NobuoGarner, CharlesSasaki, Sergio DaishiGaroli, DenisSashuk, VolodymyrGarrett Da Costa, RafaelSassi, MohamedGarrido, Narciso M.Sastre, Ana MariaGarrido-Fernández, AntónioSastre-Santos, AngelaGarroni, SebastianoSastry, JagannadhaGarrosa, ManuelSatake, HonooGarstka, Malgorzata AnnaSatake, MasayukiGarzoli, StefaniaŠatínský, DaliborGasbarri, CarlaSatish, LakkakulaGašić, UrošSato, DaisukeGaspar, CristinaSato, KazuyukiGaspar, Maria ManuelaSato, KenjiGáspári, ZoltánSato, KousukeGasparini, PierluigiSato, KyotakaGasset, MariaSato, MasamitsuGatchell, MichaelSato, MichioGatin, AndreySato, ShinobuGatti, AntoniettaSato, YusukeGatti, CarloSatoh, ShigeruGatto, FrancescaSatora, PawelGaudin, KarenSator-Katzenschlager, SabineGaudin, ValérieSatou, TadaakiGaul, DavidSatriano, CristinaGautam, MukeshSaturnino, CarmelaGautam, RekhaSatyal, PrabodhGautam, UmaSauer, DanielGavara, LaurentSauka, DiegoGavenonis, JasonSaurav, KumarGaviña, PabloSaurina, JavierGavira, JoséSavage, KayeGavrila, Adina IonutaSavage, PaulGawel, KingaSaverwyns, StevenGaweł-Bęben, KatarzynaSaviano, MicheleGawlik-Dziki, UrszulaSavic, Ivan M.Gawronska-Kozak, BarbaraSavić, JelenaGazdzicki, PawelSavina, IrinaGaze, Mark N.Savion, NaphtaliGazerani, ParisaSavoie, Jean-MichelGazzano, MassimoSavovic, SvetislavGe, XinSavy, DavideGeana, Elisabeta-IrinaSawa, TeijiGee, WilliamSawada, Jun-ichiGeelen, DannySawai, HirofumiGeerlof, ArieSawai, JunGeffroy-Rodier, ClaudeSawama, YoshinariGęgotek, AgnieszkaSawicka, BarbaraGehring, Tito A.Sawicki, TomaszGeisler-Lee, JaneSayago, AnaGelbrich, ThomasSayago, Francisco J.Gelbstein, YanivSayydi, NimaGeletii, YuriiSberveglieri, VeronicaGellrich, UrsSbirrazzuoli, NicolasGemma, SandraScafato, PatriziaGendaszewska-Darmach, EdytaScafuri, BernardinaGenders, AmandaScala, AngelaGeng, HuilingScala, ValeriaGentile, CarlaScalise, MariafrancescaGentile, DavideScalliet, CamilleGentile, FabrizioScampicchio, MatteoGentile, FrancescoScanu, Antonio MarioGentili, AlessandraScarano, AntonioGentili, DenisScarpa, Emanuele-SalvatoreGeorgakilas, VasiliosScarpa, MarinaGeorge, BlassanScarso, AlessandroGeorge, GraemeScazzina, FrancescaGeorge, SoniaSchab Balcerzak, EwaGeorgi, AnettSchabowicz, KrzysztofGeorgiades, SavvasSchäfer, EdgarGeorgiev, VasilSchander, AndreasGeorgiev, YordanSchatz, JürgenGeorgiev, Yordan NikolaevScheggi, SimonaGeorgieva, RadostinaScheiner, SteveGeorgopanos, ProkopiosSchelhaas, SonjaGeraldo Yoneyama, Kelly AparecidaSchelvis, JohannesGerardino, AnnamariaSchemmer, PeterGerasimaitė, RūtaSchennach, RobertGerasimchuk, NikolaySchenone, SilviaGerbaux, PascalScherf, KatharinaGerber, CobusScheunemann, MatthiasGerbi, VincenzoSchicho, RudolfGerding, HeinrichSchiemann, OlavGerdol, MarcoSchild, AxelGere, AttilaSchilling, StephanGeremia, SilvanoSchiraldi, ChiaraGerislioglu, BurakSchiraldi, DavidGerken, MichaelSchirhagl, RomanaGerlitz, GabiSchirrmacher, RalfGerm, MatejaSchito, Anna MariaGerman, Nadezhda A.Schlaepfer, IsabelGermanò, Maria PaolaSchlafer, SebastianGeronikaki, AthinaSchlettwein, DerckGerosa, GinoSchmeda-Hirschmann, GuillermoGerothanassis, Ioannis P.Schmid, JochenGervasi, TeresaSchmidt, BeataGervasoni, JacopoSchmidt, BerndGesslbauer, BerndSchmidt, Bernhard V. K. J.Gestal, MonicaSchmidt, ClaudiaGeszke-Moritz, MałgorzataSchmidt, MartinGewirtz, DavidSchmidt, MartinaGhaani, MasoudSchmidt, Walter F.Ghaani, Mohammad RezaSchmidtke, GunterGhalem, Bachir RahoSchmitt, TomasGhanbarian, BehzadSchmitz, ChristianGhandi, KhashayarSchmitz, John C.Ghanotakis, Demetrios DemetriosSchmitz, UlfGhantasala, MuralidharSchmutz, MarcGhasemi, RastaSchnabel, ThomasGhavami, SaeidSchneck, EmanuelGhazzal, Mohamed NawfalSchneckenburger, HerbertGhiasvand, AlirezaSchneider, BerndGhica, Mihaela-VioletaSchneider, IoanGhidini, MicheleSchneider, PetraGhilardi Lago, João HenriqueSchneider, RaphaelGhirga, FrancescaSchneider, SabineGhiviriga, IonSchneider-Schaulies, JuergenGholami, FatemehSchnepf, AndreasGhomi, MahmoudSchniepp, HannesGhosh, ArijitSchoffstall, AllenGhosh, ShobhaSchohn, HervéGhosh, SohamSchöllhorn, BerndGhosh, SoumitaScholtz, VladimírGhosh, SubrataScholz, CarmenGiacomazza, DanielaScholze, AlexandraGiacomelli, ChiaraSchomacher, LarsGiacomello, PierluigiSchorzman, Allison N.Giamarchi, PhilippeSchraermeyer, UlrichGiampieri, FrancescaSchramm, Michael P.Giampietro, LetiziaSchreiber, MichaelGiancane, GabrieleSchripsema, JanGianetti, ThomasSchröder, UweGiannakas, ArisSchroeder, GrzegorzGiannakourou, Maria C.Schuchardt, MirjamGiannopoulos, GeorgiosSchughart, KlausGiannuzzi, LedaSchulze, JohannesGianoncelli, AlessandraSchulzke, CarolaGianpiero, CeraSchulzová, VěraGiansanti, FrancescoSchumacher, UdoGiárd, Jean ChristopheSchurig-Briccio, LiciGiaretta, ElisaSchwalbe, MatthiasGiaveno, AlejandraSchwalbe, Ruth A.Gibson, ChristopherSchwaminger, SebastianGiebultowicz, JoannaSchwan, WilliamGiełdoń, ArturSchwans, Jason P.Gieleciak, RafalSchwarz, DanaGierszewska, MagdalenaSchwarz, KarlheinzGigliotti, Casimiro LucaSchwarz, MónicaGikas, EvagelosSchwarz, PatrickGil, BarbaraSchwarz, SimonaGil, Cristiane DamasSchweiker, StephanieGil, JavierSchwendt, MarekGilch, PeterSchwikkard, SianneGiles, CoreySchwinefus, JeffGiles, WayneSchymura, StefanGiliopoulos, DimitriosSciacca, Michele Francesco MariaGilje, John W.Ścibisz, IwonaGillan, Edward G.Scicchitano, PietroGillespie, James W.Sciortino, GiuseppeGillich, Gilbert-RainerSciortino, Maria TeresaGillings, Nic M.Scipione, LuigiGil-Martín, EmilioSciubba, FabioGil-Ramirez, GuzmanScoffield, JessicaGiménez Bastida, Juan AntonioScoffone, Viola CamillaGimeno, ConcepciónScognamiglio, MonicaGimpel, MatthiasScollary, GeoffGimple, Ryan C.Scotece, MorenaGinaldi, LiaScott, IainGindl-Altmutter, WolfgangScott, John AshleyGiner-Casares, Juan J.Scott, PeterGini, GiuseppinaScrima, MarioGioele, PagotScrimin, PaoloGioia, ClaudioScudiero, OlgaGioiello, AntimoScuruchi, MicheleGionfriddo, EmanuelaSczepanski, JonathanGiordano, AntonioSebastian, AimyGiorgetti, AlejandroŠebela, MarekGiorgi, GiorgioŠebesta, RadovanGiorgi, MauroSebestik, JaroslavGiorgini, ElisabettaSebilo, MathieuGiosafatto, Concetta Valeria LuciaSeca, Ana M. LGiovagnoli, StefanoSeca, Ana Maria Loureiro DaGiovannetti, RitaSecenji, AleksandarGiovannini, TommasoSecondo, AgneseGiovannucci, David R.Sedic, MirelaGiovarelli, MatteoSedlak, MilosGiraldo, LilianaSeelam, Prem KumarGirault, AlbanSeene, TeetGiri, BanabihariSegarra-Marti, JavierGirolami, FlaviaŠegota, SuzanaGirolami, MarcoSeguí Gil, LucíaGirometta, Carolina ElenaSegundo, MarcelaGirotti, Albert W.Seidel, Rüdiger W.Gismondi, AngeloSeidl, ClaudiaGitsov, IvanSeifitokaldani, AliGiuberti, GianlucaSeijas Vázquez, Julio A.Giuffrè, Angelo MariaSeipke, RyanGiuffre, OttaviaSeisenbaeva, GulaimGiuffrida, DanieleSekara, AgnieszkaGiuliani, MicheleSekatski, SergueiGiuliano, MichelaSekijima, MasakazuGiunchedi, PaoloSękowski, SzymonGiuseppe, PaladiniSekutor, MarinaGiuseppina, BasiniSelene, Sepulveda-GuzmanGiustiniano, MariateresaSelling, GordonGładkowski, WitoldSelmani, AtiđaGlasmacher, BirgitSelvaraju, Ram KumarGlass, RichardSemenova, Maria G.Glasser, Wolfgang G.Semenyuk, PavelGlattauer, VeronicaSemeril, DavidGlažar, IrenaSemreen, Mohammad H.Gleńsk, MichałSemsarilar, MonaGlibowski, PawełSen, RwikGligor, FeliciaSenesi, GiorgioGlinkowski, WojciechSenesi, NicolaGlinton, KevinSenge, MathiasGlišić, BiljanaŞenilă, MarinGloaguen, VincentSenkovska, IrenaGłogowski, RobertSensini, AlbertoGlomm, Wilhelm R.Seo, Dong-KyunGlover, Stephen A.Seo, Ho SeongGlukhov, EvgeniaSeo, Hyun OokGlukhova, Olga E.Seo, SoonminGlushkov, Dmitrii O.Sepcic, KristinaGłuszyńska, AgataSepe, LuciaGnat, SebastianSequeda-Castañeda, Luis G.Gniadek, MariannaSequeira, César A. C.Gniewosz, MałgorzataSerafín, VerónicaGöbel, MichaelSeravalli, JavierGóbi, SándorSerefko, AnnaGobin, IvanaSereikaite, JolantaGoclon, JakubSereno, DenisGodin, BianaSerezhkin, Viktor N.Godinho, Maria HelenaSergeant, KjellGodlewska-Żyłkiewicz, BeataSergeev, YuriGodyń, JustynaSergeyev, VladimirGoff, Gaëlle LeSergi, ConsolatoGoh, GuodongSergio, LucreziaGohain, Priyakshee BorpatraSerio, AnnalisaGohda, EiichiSerpooshan, VahidGoi, AnnaSerra, RaffaeleGojo, SatoshiSerra, StefanoGolba, SylwiaSerrador Peiró, Juan M.Goldeman, WaldemarSerralheiro, Maria LuísaGolewski, PrzemysławSerrano, Dolores R.Golic-Grdadolnik, SimonaSerrano, MaríaGolovko, MikhailSerrano, MarioGoltsov, AlexeySerrano-Aroca, ÁngelGoltz, Douglas M.Serreli, GabrieleGolub, EyalSertić, MirandaGolub, IgorServili, MaurizioGomes Da Cruz, AdrianoSessini, ValentinaGomes, Ana T. P. C.Sestili, FrancescoGomes, Carlos MartinsSestito, SimonaGomes, Clara S. B.Seto, EdwardGomes, Joao FSeto, ShigekiGomes, Marcelo Gomes DeSetyaningsih, WidiastutiGomes, NelsonSetzer, William N.Gomes, Paula A. C.Seung-Hoon, BaekGomes-da-Silva, Lígia C.Sevastre, BogdanGómez Barrios, XiomarSeverino, BeatriceGómez Castellà, CristinaSevic, DragutinGómez De Cedrón, MartaSevilla, MichaelGómez García, Carlos J.Sevilla-Morán, BeatrizGómez Ocampo, IvánSewani-Rusike, ConstanceGómez, Ana MaríaSgarbossa, PaoloGómez, Jose LorenzoSgherri, CristinaGomez, NidiaSgorbini, BarbaraGómez-Ariza, José LuisSgouros, AristotelisGómez-Bengoa, EnriqueSgrignani, JacopoGómez-Cámer, Juan LuisSgroi, Mauro FrancescoGómez-Cápiro, OscarShabaninejad, MehdiGomez-Pilar, JavierShabatina, TatyanaGomez-Ruiz, SantiagoShafahi, MaryamGomez-Sainero, Luisa MariaShah, Jindal K.Gomha, SobhiShahid, SaroashGoncalves, Alexandre A.S.Shaik, Anver BashaGonçalves, Ana M. M.Shaik, Mohammed RafiGonçalves, BrunoShakeel, FaiyazGonçalves, M. Sameiro T.Shakhtshneider, Tatyana P.Gonçalves, VirginiaShakibaei, MehdiGoncharuk, OlenaShakya, Viplendra P. S.Gonda, SandorShalaby, MohamedGondova, TatanaShan, WenqianGonec, TomasShang, ChaoqunGoněc, TomášShang, QingsenGong, MaxShang, ZhuoGong, XingchuShankar, EswarGoni, FelixShankhari, PritamGonzales, Cara B.Shanmugam, MuruganandanGonzález Domínguez, RaúlShao, FangweiGonzález Laredo, Rubén F.Shao, HuaGonzalez Viejo, ClaudiaShao, HuaiyuGonzález, Concepción C.Shao, LongquanGonzález, Florenci V.Shao, LuGonzalez, Juan M.Shao, XueguangGonzález, M. CarmenShao, YihanGonzález, María De Las NievesShapiro, BruceGonzález, MartaSharan, ShyamGonzalez, OskarSharker, Shazid Md.Gonzalez, VictorSharma, AbhisheakGonzalez, ZoiloSharma, AshutoshGonzalez, ZoraidaSharma, GeetanjaliGonzález-De-Peredo, Ana V.Sharma, IshaGonzález-García, JorgeSharma, KavitaGonzález-Laredo, Rubén FranciscoSharma, PrashantGonzalez-Lezcano, Roberto AlonsoSharma, PriyankaGonzález-Marrero, JoaquinSharma, RitinGonzález-Maya, LeticiaSharma, ViragGonzález-Muñiz, RosarioSharpe, Shaun M.González-Pérez, José A.Shateyi, StanfordGonzález-Sálamo, JavierShaul, Yoav D.Gonzalez-Stuart, Armando EnriqueShavandi, AminGonzález-Vallinas, MargaritaShaw, LisaGonzález-Vergara, EnriqueShaw, SubrataGonzalez-Vicente, AgustinShaya, JanahGonzález-Zamora, EduardoShchyogolev, SergeiGoormaghtigh, ErikShears, SthephenGopalakrishnan, GopakumarSheats, KatieGopalakrishnan, RangaSheen, Horn-jiunnGóra, ArturSheidaei, AzadehGorbacheva, LiubovSheldon, RogerGorczynski, AdamSheldrake, GaryGoretti, EnzoSheldrake, HelenGorgan, LucianShellaiah, MuthaiahGorgieva, SelestinaShen, BinGori, AntonellaShen, JiangchuanGorinstein, ShelaShen, Jian-yingGorkunov, Maxim V.Shen, LimingGornas, PawelShen, QiangGornitzka, HeinzShen, WeifengGornostaev, Leonid M.Shen, XiGornowicz, AgnieszkaShen, XiaoGornowicz-Porowska, JustynaShen, XingguiGoroncy, AlexanderShen, XiulongGorpas, DimitrisShen, YiGorski, ŁukaszShen, YiouGorynski, KrzysztofShen, YudongGościańska, JoannaShen, YuemaoGoseki, RaitaShen, ZhiqiangGosetti, FabioShenashen, Mohamed A.Gośliński, TomaszShenderovich, Ilya G.Gospodarek, JaninaSheng, RongGossage, R. A.Sheng, RuilongGoto, MasuoSheng, YueGoto, MotonobuShenoy, PriyankGoto-Inoue, NaokoSherwood, JamesGotor, Alicia AyerdiSheth, BhavwantiGotor-Fernández, VicenteShevtsov, MaximGotsbacher, MichaelShi, HaitaoGottwald, EricShi, LeiGoua, MarieShi, ShichengGoudoulas, ThomasShi, WenGoula, Maria A.Shi, YangGoulas, VlasiosShi, YushengGourlay, Louise J.Shi, ZengqianGouveia, Rubia FigueredoShiao, Young-JiGouws, ChrisnaShiau, LiedingGovan, JosephShiba, FumiyukiGowda, KrishneShiba, KiyotakaGoyanes, AlvaroShibaev, PetrGozin, MichaelShibata, IkuyaGrabarczyk, MałgorzataShibatomi, KazutakaGrabarek, Beniamin OskarShibayama, NobukoGrabchev, IvoShibuya, MasatoshiGrabek-Lejko, DorotaShieh, Tzong-MingGrabner, Daniel S.Shields IV, C. WyattGrabowski, SlawomirShiga, TakuyaGracin, DavorShiina, MarisaGraczyk-Jarzynka, AgnieszkaShikov, AlexanderGradoni, LuigiShikov, Alexander N.Gradov, Oleg V.Shimada, Ken-ichiroGradzielski, MichaelShimada, MasakoGraebin, CedricShimada, YasuhitoGraham, DavidShimanouchi, ToshinoriGramignoli, RobertoShimazu, ShogoGramza-Michałowska, AnnaShimba, ShigekiGranados-Principal, Sergio M.Shimizu, FlavioGranato, DanielShimizu, MakotoGranchi, CarlottaShimizu, SojiGrande Tovar, Carlos DavidShimpei, IikuniGrandemange, StéphanieShimura, TatsuoGrandjean, CyrilleShin, DongyunGranica, SebastianShin, Hyun-SangGranja, TiagoShin, Jae-HoGrant, GeorgeShin, SoyoungGrases, FelixShin, Young G.Grässel, SusanneShinada, TetsuroGrassi, SilviaShinde, Arti V.Grasso, GiuseppeShinde, Surendra KrushnaGrasso, SimonaShinoda, YoGrattoni, AlessandroShinohara, MitsuruGrause, GuidoShioda, NorifumiGray, ChristophertShioda, TatsuoGraziano, GuellaShiotari, AkitoshiGraziano, Sossio FabioShipley, PaulGreatrex, BenShiragami, TsutomuGrebenc, TineShirahata, TatsuyaGreco, ToddShirai-Matsumoto, KeikoGreen, IvanShirakami, YoheiGreen, MichaelShirasuna, KoumeiGreenberg, Robert M.Shiri-Sverdlov, RonitGreenhalgh, DavidShivange, AmolGreenlief, C. MichaelShiwarski, Daniel J.Greenspan, PhillipShnyder, SteveGreenwood, Michael T.Shoda, MakotoGreer, EdytaShoemaker, CharlesGreetham, DarrenShoji, MitsuoGregor, IngoShoji, YoshiakiGregori, AlbertoShornick, LaurieGrembecka, MałgorzataShoseyov, OdedGressent, FrédéricShpigelman, AviGribble, GordonShrestha, NamitaGribskov, MichaelShu, XinhuaGrice, Jeffrey E.Shuck, ChristopherGrider, ArthurShui, Hao-aiGriesbeck, Axel G.Shuichi, MoriGriffini, GianmarcoShukitt-Hale, BarbaraGriffiths, WilliamShults, Elvira E.Grigoraš, Anca GiorgianaShun, YaoGrilc, MihaShurpik, DmitriyGrill, MagdalenaSi, RuizheGrillo, FedericoSiamidi, AngelikiGrillo, GiorgioSiamwala, Jamila H.Grimaldi, AnnalisaSiatka, TomášGrimaldi, GiovannaŠic Žlabur, JanaGrimm, Marcus O. W.Sicari, VincenzoGrishin, MaximSiciliano, CarloGrishko, VictoriaSicinski, RafalGrisoni, FrancescaSiddique, Abu BakarGritsan, Nina P.Sideratou, ZiliGrivas, IoannisSidorov, PavelGrob, KoniSidorova, YuliaGrochowski, CezarySieniawska, ElwiraGröger, ThomasSier, CornelisGronwald, WoframSieradzan, AdamGross, Andrew J.Sierecki, EmmaGroß, HaraldSieroslawska, AnnaGross, Joshua B.Sierra-Campos, ErickGrossi, GiancarloSievänen, ElinaGrosso, ClaraSiewert, BiankaGrosso, GiuseppeSigala, SandraGrotli, MortenSignore, GiovanniGroundwater, PaulSignorini, CinziaGrubeša, Ivanka NetingerSikalidis, AngelosGrudnik, PrzemyslawSikora, JanuszGrul’ová, DanielaSikorska, EmiliaGrumet, RebeccaSikorski, AleksanderGruszczynska-Biegala, JoannaSikorski, ArturGruzdev, MatveySilacci, PaoloGryaznevich, Mikhail P.Silberring, JerzyGrygier, AnnaŠiler, BranislavGryn’ova, GannaŠilha, DavidGrynkiewicz, GrzegorzSiligardi, GiulianoGryszkin, ArturSilina, YuliyaGrzegorczyk, IzabelaSilipo, AlbaGrzyb, JoannaSiliqi, DritanGu, ZhenSilva Filho, EdsonGuadagni, FiorellaSilva Moura, Elisabete FerreiraGualerzi, AliceSilva, Adriana RibeiroGuamis, BuenaventuraSilva, Amelia M.Guangjiu, ZhaoSilva, ArturGuazzelli, LorenzoSilva, Carlos AlbertoGubatan, JohnSilva, CatarinaGubitosa, JenniferSilva, Catarina L.Guda, Alexander A.Silva, Dr. Márcio S.Gudiminchi, RamaSilva, FilomenaGudiña, EduardoSilva, Ines Vieira DaGudiukaité, RenataSilva, Maria FátimaGudkov, Sergey VSilva, Nuno A.Gudmundsdottir, Anna D.Silva, PaulaGudmundsson, Jon TomasSilva, RuiGudoshnikov, SergeySilva, Saimon MoraesGuedes, Rita C.Silva, Severiano R.Guedes-Alonso, RaycoSilva, Vera L. M.Guerre, PhilippeSilva-Espinoza, Brenda A.Guerrero, José Alejandro HerediaSilvagno, FrancescaGuerrieri, NicolettaSilva-Martínez, SusanaGuerrini, AlessandraSilveira, Nádya P. DaGuerrini, GabriellaSilvestre, Armando J. D.Guerrini, MarcoSilvestre, SamuelGuescini, MicheleSilvestri, CristoforoGuglielmi, PaoloSim, JaehoonGuglielmotti, ManfrediSim, UkGuha, PrasunŠimáček, PavelGuhathakurta, SubhrangshuSimal, JesusGui, RijunŠimėnas, MantasGuibal, EricSimeoli, RaffaeleGuichard, ElisabethSimeonov, VasilGuidi, PatriziaSimes, DinaGuidotti, MatteoSimion, MonicaGuijun, XianSimirgiotis, MarioGuillena, GabrielaSimko, FedorGuillén-Bejarano, RafaelSimmons, KatieGuillén-Sánchez, Dominico A.Simões, PedroGuillet-Nicolas, RemySimões, Zilá Luz PaulinoGuillou, Claude G.Simon, IstvanGuiné, RaquelSimon, JozsefGuiot, CaterinaSimon, JulianGuitton, JérômeSimon, PeterGüldiken, NurdanSimonato, BarbaraGulea, MihaelaSimončič, BarbaraGulic, TamaraSimone, Angela DeGulicovski, JelenaSimone, VincenziGulino, AntoninoSimonescu, Claudia MariaGullo, MaurizioSimonetti, GiovannaGullon, BeatrizSimoni, FrancescoGullon, BetatrizSimonpietro, AgnelloGulyaeva, LyudmilaSimova-Stoilova, Lyudmila PetrovaGumieniczek, AnnaSimpson, Bradley S.Gumienna, MałgorzataSimpson, Thomas J.Gumul, DorotaSims, GeraldGun’ko, Vladimir MSimserides, ConstantinosGun’ko, Vladimir M.Sin, JonGunaje, JayaramaSinanoglou, VassiliaGunasekara, ChamilaSinclair, AndrewGuncheva, MayaSingaraju, AdityaGunda, VenugopalSinger, StevenGundamaraju, RohitSingh, AlokGunia-Krzyżak, AgnieszkaSingh, AnilGuńka, PiotrSingh, AnirudhaGünther, StefanSingh, Bir BahadurGünther, UlrichSingh, GurwinderGuo, Chih-HungSingh, KamalendraGuo, DandanSingh, KulbirGuo, FenghaiSingh, MogieGuo, Hao-BoSingh, NishaGuo, JingshuSingh, ParveshGuo, Ju-TaoSingh, PriyankaGuo, Ming-quanSingh, RanjeetGuo, NaSingh, RuchiraGuo, TingSingh, Rupesh KumarGuo, WeiminSingh, Santosh KumarGuo, XiaoyuSingh, Sharda P.Guo, XinboSingh, SomnathGuo, XingzhongSingh, Surya P.Guo, YongSingha, SubhankarGuo, Zhan-HuSingla, BhupeshGuo, ZhefengSinha, Sudarson SekharGuo, ZhengSinha-Ray, SumanGuo, Zong ShengSinjari, BrunaGuochen, BaoŠinko, GoranGupta, KajalSinn, GerhardGupta, SanjaySintsova, OksanaGupta, SushimSinyukov, AlexanderGurevich, Vsevolod V.Siodłak, DawidGurikov, PavelSiopa, FilipaGurina, DaryaSipos, EmeseGurrapu, SreeharshaSipos, KatalinGus’kov, VladimirSipos, PálGusarov, Sergey I.Sippl, WolfgangGuselnikova, Olga AndreevnaSiracusa, RosalbaGushchin, Artem L.Siramshetty, Vishal B.Gushina, IrinaSirbu, OvidiuGusiatin, Zygmunt MariuszSiren, HeliGuskos, NikoSiskos, Michael G.Guskova, OlgaSistla, SrinivasGust, RonaldSita, GiuliaGustafson, KirkSitarek, PrzemysławGutberlet, ThomasSithole, BruceGuterman, RyanSivaev, Igor B.Gutierrez Ramirez, ManuelSivalingam, PeriyasamyGutiérrez Ramírez, ManuelSivathanu, VivekGutiérrez, LauraSizochenko, NataliaGutierrez, LeopoldoSkaf, Dorothy W.Gutiérrez, MargaritaSkała, EwaGutierrez, OsvaldoSkalicka-Woźniak, KrystynaGutierrez-Aguilar, ManuelSkalicky, MilanGutiérrez-Arzaluz, MirellaSkalniak, LukaszGutiérrez-Gallego, RicardoSkalova, LenkaGutiérrez-Gamboa, GastónSkaltsa, LianaGutierrez-Merino, CarlosSkandalis, NikolaosGutiérrez-Venegas, GloriaSkarżewski, JacekGuzik, MaciejSkelly, DeanneGuzman-Puyol, SusanaSkenderidis, ProdromosGwalani, BharatSkerget, MojcaGwiazdowska, DanielaSkibiński, RobertGyarmati, BenjáminSkoko, John J.Gyémánt, GyöngyiSkonieczna, MagdalenaGyepes, RóbertSkórczewska, KatarzynaGyöngyösi, MariannSkorik, Yury A.H. Gonzalez, DanielSkouta, RachidH. Moreno, Paulo RobertoSkrabana, RostislavHA, Hyung-HoSkrypnik, LiubovHa, Jung-HeunSkrypnik, Liubov N.Ha, Kwon-SooSkrzypczak-Pietraszek, EwaHabala, LadislavSkurnik, MikaelHabas, KhaledSkuza, LidiaHabata, YoichiSkwarek, EwaHabaue, ShigekiSlama, James T.Habermehl-Cwirzen, KarinSlaninová, IvaHabuda-Stanić, MirnaSleigh, JamesHackl, ThomasSlim, AzouziHada, MasahikoSlim, SmaouiHadden, KyleSlimestad, RuneHadian, KamyarŚliwa, IzabelaHadjikakou, SotirisŚliwińska-Wilczewska, SylwiaHadjipavlou-Litina, DimitraSliwinski, TomaszHa-Duong, Nguyet-ThanhSliwka, Hans-RichardHadwiger, Jeffrey A.Sloan, KennethHaendler, BernardSlobodnikova, LiviaHaertle, ThomasSłoczyńska, KarolinaHafiane, AnouarSłomińska-Wojewódzka, MonikaHagar, MohamedSlominski, AndrzejHagberg, CarolinaSłomkowski, StanislawHagelueken, GregorSloop, JosephHageman, JosSlowinska, KatarzynaHahn, FriedrichŚlusarczyk, SylwesterHaidar, SamerSmailyte, GiedreHaidar, Ziyad S.Šmejkal, KarelHaider, NorbertSmeriglio, AntonellaHajizadeh, SolmazSmet, MarioHalász, KatalinSmet, PhilippeHalcovitch, Nathan RSmiesko, MartinHalder, Amit KumarŚmigielski, Krzysztof B.Halder, AvikSmiljanić, KatarinaHalet, Jean-FrançoisSmirnov, Nickolay N.Haliński, Łukasz P.Smith, JenniferHallam, TobySmith, KerryHallmann, EwelinaSmith, Lloyd M.Ham, Young WanSmith, MarkHamano, YoshimitsuSmith, Micholas DeanHamasaki, YoshifumiSmith, ZacharyHamashima, YoshitakaSmulko, JanuszHamba, HidenoriSmyk, BogdanHamdan, NasserSmyth, JamesHameed, AhsanSnapper, MarcHamilton, ChrisSnegur, LubovHamman, JoiasSnežana, PapovićHammarstrom, JaneSnopek, LukasHammer, MartinSnopiński, PrzemysławHammerl, Jens AndreSnow, NicholasHamon, Jean-RenéSnow, Nicholas H.Hampp, NorbertSnowden, TimothyHampp, Norbert A.Snyder, NicoleHan, BingnanSoares, Ana RaquelHan, DongSoares, Bluma G.Han, DongWoonSoares, Maria Isabel L.Han, FelicitySoares, PedroHan, HeyouŠob, MojmírHan, JaehongSobczak, PawełHan, JianlinSobczak-Kupiec, AgnieszkaHan, JinsongSobeh, MansourHan, Jong WonSobiesiak, MagdalenaHan, QianSobkowski, MichalHan, Quan-BinSobolev, ArkadijHan, Se JongSobotta, ŁukaszHan, Soo DeokSocaci, SoniaHan, Sung GuSocaciu, CarmenHan, XiuguoSocała, KatarzynaHan, YouSocas-Rodríguez, BárbaraHan, ZhenlinSocha, KatarzynaHanai, ToshihikoSocha, RobertHanania, MichelSochaj-Gregorczyk, Alicja MariaHanaoka, ToshiakiSocol, MarcelaHanawa, TakehisaSöderberg, Björn C. G.Hancu, GabrielSoe, KentHanda, AkihiroSoengas, Raquel G.Handa, MakotoSoesoo, AlvarHandzlik, JadwigaSoeta, TakahiroHanefeld, UlfSogawa, ChiharuHaneke, EckartSogkas, GeorgiosHanemann, ThomasSohrabi, SalmanHang, TaijunSoica, CodrutaHanganu, DanielaSokolova, Maria PetrovnaHanifehpour, YounesSokolová, RomanaHannedouche, JeromeSolache-Ríos, Marcos JoséHano, ChristopheŠolaja, Bogdan A.Hansen, EspenSolano, FranciscoHansen, NielsSolè, DanielHansford, KarlSoler, Miguel ÁngelHanton, LyallSolieri, LisaHao, JinsongSoliman, MahmoudHao, ZhaoSoliman, Sameh S. M.Haouas, MohamedSolís, Luis Fernando MagañaHaponiuk, JózefSołoducho, JadwigaHaque, ArifulSolomon, MelaniHara, KenjiSolomonov, BorisHaraldsen, Jason T.Solomonov, InnaHarashima, NanaeSoloshonok, VadimHarasym, JoannaSolovieva, SvetlanaHarbertson, JamesSolovyev, NikolayHardacre, AllanSolyanikova, InnaHarijan, Rajesh KSolymar, MargitHarijith, AnanthaSomavat, PavelHarms, AmySommerer, NicolasHarnisz, MonikaSomoza, ÁlvaroHaroun, RicardoSomsák, LászlóHarp, Joel M.Son, Ki-HoHarper, JamesSon, SeungukHarrington, Peter De BovesSonavane, ManojHarris, ChrisSoncin, FrancescaHarris, Edward N.Sone, HidekoHarris, TrevorSong, BaoanHarrison, JoeSong, JianweiHarrison, PatrickSong, JianxunHarrison, Paul H. M.Song, Jong-WonHarrison, SabineSong, JuntaeHarrison-Bernard, LisaSong, MinjungHarscoat-Schiavo, ChristelleSong, XuezhengHart, Peter C.Song, Yang-HeonHartenbach, IngoSong, YuandaHartl, FrantisekSong, ZhiyiHartman, MariuszSong, ZiyuanHartman, Matthew C. T.Sonnenberg, AntonHartung, JensSonnier, RodolpheHaruki, MitsuruSonowal, HimangshuHarvey, PetaSoond, Surinder M.Has, CristinaSoran, Maria-LoredanaHasan, AbsharSordello, FabrizioHasan, MehediSørensen, Jens LauridsHasan, MurtazaSorensen, JohnHasan, Syed SaifSoreq, HermonaHasanuzzaman, MirzaSorg, OlivierHasegawa, MakotoSoria, Ana CristinaHasegawa, MorifumiSoriano, José MiguelHashem, AbulSorokin, AndreyHashemi, MohtadinSorokina, Irina V.Hashidzume, AkihitoSorrentino, AndreaHashimoto, MakotoSorrentino, Nicolina CristinaHashimoto, MasanoriSosa Díaz, TeresaHashimoto, MasaruSosic, IzidorHashimoto, TakeshiSosnowska, DorotaHashimoto, TomokoSosnowska, KatarzynaHashimoto, YoshitakaSosnowski, MarcinHasiuk, Franciszek J.Sotelo Mundo, RogerioHaskó, GyörgySotiriou, GeorgiosHassan, AhmedSoto, Alejandro PresaHassan, ErrolSoto, CarmenHassan, MohamedSoto-Hernández, MarcosHassan, Sherif T. S.Sotomayor, NuriaHasumi, KeijiSottero, BarbaraHatada, RurikoSou, KeitaroHatae, NoriyukiSoukoulis, ChristosHatano, AkihikoSoural, MiroslavHatano, TsutomuSousa, CarmenHatano, YuichiroSousa, Joana L. C.Hathaway, NathanielSousa, Maria JoãoHati, SantanuSousa, Sérgio F.Hatzakis, EmmanuelSousa, Sérgio FilipeHatzakis, NikosSova, MatejHatzdimitriou, EffieSovadinova, IvaHatzilazarou, StefanosSoveral, GraçaHatzpolous, PolydefkisSovinco, FabioHaudecoeur, RomainSowa, IreneuszHaufe, GünterSpaccini, RiccardoHaugen, Håvard J.Spadaccini, RobertaHaupt, MichaelSpagnolo, VincenzoHaurie, LaiaSpagnuolo, CarmelaHauser, PeterSpain, Jim C.Hausmann, MichaelSpallarossa, AndreaHavelek, RadimSpanedda, MariaHavlik, JaroslavSpasov, Alexander A.Havran, LudekSpassov, TonyHawash, ZaferSpatari, SabrinaHawes, Chris S.Specht, AlexandreHawrył, Anna M.Spelzini, DaríoHawrył, MirosławSpencer, JohnHay, SamSpengler, GabriellaHayashi, Mariko KatoSpetea, MarianaHayashi, NorikoSpieckermann, FlorianHayashi, YoshihitoSpiegel, MartinHayes, MariaSpiegler, VerenaHayes, Sidney J.Spiers, AndrewHaynes, RichardSpingler, BernhardHayward, StevenSpínola, VítorHazai, LászlóSpiridigliozzi, LucaHe, ChengweiSpirina, Liudmila V.He, DongfengSpivak, DavidHe, GuanjieSpiwok, VojtechHe, HailunSpizzirri, U. GianfrancoHe, HuijunSplichalova, AllaHe, LixiaSplinter, RobertHe, PumingSpohr, Klaus MichaelHe, WangxiaoSponer, JuditHe, XinSpratt, Donald E.He, YongSpur, Bernd W.He, ZhiguoSpyroulias, GeorgiosHeavey, SusanSquillacioti, CaterinaHeber, DieterSquillaro, TizianaHeberden, ChristineŚrednicka-Tober, DominikaHebrant, MarcSreejith, Kamalalayam RajanHeck, JürgenSreerama, LakshmaiahHedeland, MikaelSresht, VishnuHedhammar, MySrinivas, KeerthiHedstrom, LizbethSripathy, SmithaHee, Ay ChingSrirangan, KajanHeere, MichaelSrivastava, PranayHeffeter, PetraSrivatsan, MalathiHegarty, MatthewSroda-Pomianek, KamilaHegde, VijaySroka, ZbigniewHegedűs, LászlóSroka-Bartnicka, AnnaHegedűsová, AlžbetaSt. Louis, IrinaHeinrich, BenoîtStacchiotti, AlessandraHejna, AleksanderStachowska, EwaHelesbeux, Jean-JacquesStączek, SylwiaHellweg, ThomasStadie, Nicholas P.Hellwig, MichaelStahl, FrankHeltai, GyorgyStalikas, ConstantineHémadi, MiryanaStamatatos, TheocharisHendriks, Hans H.Stambuk, NikolaHenen, Morkos A.Stan, MirunaHeng, Nicholas C. K.Stana, Anca-DanielaHengesbach, MartinStanciu, Gabriela-DumitritaHenke, AndreasStancu, Camelia SorinaHennig, SvenStandal, Inger B.Henrich, Curtis J.Stanford, Fatima CodyHenrique, CoutinhoStanicová, JanaHenriques, Bárbara J.Staniek, HalinaHenriques, Marta H. F.Stanimirova, IvanaHensley, AlyssaStanisz, BeataHeras, FranciscoStankevic, MarekHerbert, JohnStankova, IvankaHerchel, RadovanStanković, DaliborHerckes, PierreStankovic, MilanHerczegh, PálStanovnik, BrankoHerdewijn, PietStare, JernejHermann, GerritStarecki, TomaszHermann, PetrStarek, MałgorzataHermanowicz, JustynaŠtarha, PavelHermenean, AncaStark, Amy L.Hernadi, KlaraStark, Lesley A.Hernández Saz, JesúsStarowicz, MałgorzataHernandez, AgustinStarykevich, MaksimHernández, Eliud AvierStarynowicz, PrzemyslawHernández, FranciscaStarzak, KarolinaHernandez, JesusStarz-Gaiano, MichelleHernandez, ReinierStasiak-Różańska, LidiaHernández-Barajas, JoséStaszak, KatarzynaHernández-Carlos, BeatrizStaszczak, MagdalenaHernandez-Escobedo, QuetzalcoatlStatt, AntoniaHernández-Hernández, OswaldoStatti, GiacarloHernández-López, HiramStavber, StojanHernández-Mesa, MaykelStavrianidi, AndreyHernández-Montelongo, JacoboStawiński, JacekHernández-Núñez, EmanuelStawny, MaciejHerold, NikolasSteenari, Britt-MarieHerráez-Hernández, RosaSteenekamp, JanHerranz, FernandoStefanelli, ManuelaHerranz, Maria AngelesStefania, FilosaHerrebout, WouterŠtefanić, Petra PeharecHerrera Zaldivar, ManuelStefanidou, Maria E.Herrera, RaquelStefanowicz-Hajduk, JustynaHerrera-Herrera, Antonio V.Stefanska, AnnaHerrero-Martínez, José ManuelStefanucci, AzzurraHerres-Pawlis, SonjaSteger, GerhardHertz, Daniel L.Steger, GertrudHetényi, CsabaSteinbach-Rankins, JillHeuer-Jungemann, AmelieSteinbeck, ChristophHeusch, GerdSteindel, MárioHibbitts, AlanSteinhauer, StephanHibi, TakaoSteinhoff, UweHickey, NealSteinmann, Stephan N.Hidalgo Herrador, José MiguelStellato, FrancescoHiejima, YusukeStellavato, AntoniettaHierro, Isabel DelStelmach-Mardas, MartaHigashi, KyoheiStelmachowski, PawełHigashi, TaishiStepanic, VisnjaHigashino, TomohiroStephen, Michael RajeshHiggs, DavidStephens, GaryHIjikata, AtsushiStepien, EwaHikawa, HidemasaStępień, MarcinHikichi, ShiroStepnicka, PetrHildreth, EasonŠtěpnička, PetrHildt, EberhardStępnik, MaciejHill, GrantStępniowska, AnnaHill, JonathanSter, CélineHill, StephenStern, RaivoHillard, ElizabethSterner, OlovHinds, Terry D.Stetsyshyn, YurijHines, Rochelle M.Steuer, ChristianHingorani, DinaSTEVANOVIC, JevrosimaHinman, SamuelStewart, JamesHintzen, HubertusStewart, JasonHirahara, MasanariStine, Keith J.Hirano, KojiStiriba, Salah-EddineHirano, MasafumiStirke, ArunasHirashita, TsunehisaStobiecka, MagdalenaHirata, TetsuyaStobiecki, MaciejHirata, YokoStocchero, MatteoHirata-Tsuchiya, ShizuStoch, PawełHiroaki, HidekazuStock, Naomi L.Hirota, KiichiStocks, MichaelHirsch, ThomasStoddard, ShanaHirva, PipsaStodulski, MaciejHiscock, JenniferStogniy, Marina Yu.Hix, GaryStoica, AnicutaHlaváč, ViktorStoikov, Ivan IvanovichHo, Chi-TangStojakowska, AnnaHo, JunmingStojanoff, VivianHo, MengfeiStojanović, Gordana S.Ho, Wing ShingStojko, JerzyHocek, MichalStokke, Bjørn TorgerHochlaf, MajdiStokowa-Sołtys, KamilaHockova, DanaStolarczyk, AgnieszkaHodac, LadislavStolz, AndreasHodges, H. CourtneyStornaiuolo, MarianoHodgkinson, JamesStorsberg, JoachimHodgson, David R. W.Stove, Christophe P.Hofbauer, StefanStoyanova, AlbenaHofer, AndersStraatsma, TjerkHoff, Bård HelgeŠtrac, Dubravka ŠvobHoffman, RhondaStraniero, ValentinaHofmann, UteStrankowska, JustynaHogwood, JohnStrankowski, MichalHojo, FuhitoStrappe, PadraigHolak, Tad A.Strati, Irini F.Holb, ImreStratikos, EfstratiosHolder, AlvinStraus, Suzana K.Holec, JosefStrauss, JohannesHolen, ElisabethStrauss, LauraHolien, JessicaStrehmel, VeronikaHollenstein, MarcelStrelec, IvicaHollmann, AxelStrianese, MariaHollmann, EricStripp, Sven T.Holmberg, KristerStroffekova, KatarinaHolmes, NeilStrojny-Cieslak, BarbaraHolsinger, DamainStroupe, M. ElizabethHolt, RobertaStrout, Douglas L.Holt, Stephen A.Strozzi, MatteoHoltmann, DirkStrube, JochenHolynska, MalgorzataStruga, MartaHomaeigohar, ShahinŠtrukil, VjekoslavHomem-de-Mello, MauricioStrungaru, Stefan-AdrianHomonnay, ZoltanStruszczyk, Marcin HenrykHoncharenko, MalgorzataStryczewska, Henryka DanutaHoncová, PavlaStrzelec, KrzysztofHondal, RobertStrzemiecka, BeataHonermeier, BerndStrzemski, MaciejHong, Ic-PyoStuani, LucilleHong, JonginStuart-Williams, HilaryHong, JunStuchlik, StanislavHong, KuiStudzinska-Sroka, ElzbietaHong, Mee YoungSturtzel, CaterinaHong, YuningStürup, StefanHonório, KáthiaStushnoff, CecilHooda, JagmohanStutz, BernardoHook, IngridStylianakis, MinasHopkins, Benjamin D.Stylianakis, Minas M.Hopkinson, RichardSu, Chie-ShaanHoracek, MichaSu, DanHorecka, BeataSu, HongmeiHorhat, Florin GeorgeSu, PeifengHori, AkikoSu, WeihengHorino, YoshikazuSu, Wen-TaHornak, JaroslavSu, YichiHornak, Joseph P.Su, Zheng-YuanHornemann, ThorstenSuarez-Alcantara, KarinaHörner, GeraldSuaud, NicolasHorrocks, RichardSubach, Fedor V.Horti, AndrewŠubarić, DragoHortobagyi, TiborSubczynski, Witold K.Horvath, AneliaSubirats, XavierHorvath, BalazsSubramanian, ChitraHorvath, DragosSubramanian, PalaniappanHorváth, JuditSubramanian, ParthasarathiHorzmann, KatharineSucci, MariantoniettaHosein, Ian DeanSuchanicz, JanHošek, JanSuckling, Colin J.Hoshino, NorihisaSud’ina, Galina F.Hosmane, NarayanSuddala, Krishna ChaitanyaHosokawa, YoichiroSudo, AtsushiHosomi, RyotaSuea-Ngam, AkkapolHosório, HugoSugahara, TakuyaHossain, A. S. M. Monjur AlSugai, TakeshiHossain, EkramSugamata, KohHossain, MarufSugawara, AkihiroHossain, Md AshrafSugawara-Narutaki, AyaeHossain, Mohammad ZakirSugier, DanutaHošťálková, AnnaSugier, PiotrHosur, VishnuSugimoto, MasahiroHosztafi, SandorSugimura, KazukiHou, BoSuh, Hyung JooHou, Duen-RenSuh, JoonhyukHou, QidongSuharoschi, RamonaHou, XianghuiSujin, HoshiHou, ZhaoshengSukhikh, TaisiyaHouen, GunnarSukocheva, OlgaHoujou, HirohikoSulaeva, IrinaHoung, Jer-YiingSulek, AnnaHoury, Walid A.Suleria, Hafiz Ansar RasulHousecroft, CatherineSuliburska, JoannaHouweling, PeterSulka, Grzegorz DariuszHouzé, PascalSułkowska-Ziaja, KatarzynaHowell, BobSulonen, MiraHowells, LynneSülsen, ValeriaHowes, PhilipSulyok, MichaelHoyos, PilarSumby, ChristopherHrabal, RichardSumczynski, DanielaHrckova, GabrielaSummo, CarmineHrenar, TomicaSun, CaijunHroboňová, KatarínaSun, ChengwenHsia, Shih-MinSun, ConroyHsieh, Chang-WeiSun, DiHsieh, KuangwenSun, DuanpingHsieh, Lu-ShengSun, GuohuiHsieh, Ming-JuSun, I-WenHsieh, Tusty-JuanSun, Jing ZhiHsieh, Yi-HsienSun, LvhuiHsieh, YvesSun, Qing-FuHsin, Kun-YiSun, ShaolongHsu, Chih-ChiehSun, XiaodanHsu, Chin-LinSun, YingHsu, Day-ShinSun, Yun-LuHsu, Hao-JenSun, ZhiweiHsu, I-JuiSunatsuki, YukinariHsu, Ming-HuaSundaram, PaulHsu, ToddSundararajan, RajiHsu, Yu-KueiSundarrajan, SubramanianHsueh, Yi-HuangSundholm, DageHu, ChanglingSundman, OlaHu, ChenlinSung, Ping-JyunHu, FangzhongSung, Wen-ChiehHu, FuliangSungthong, RungrochHu, GaizunSuntharalingam, KogularamananHu, GuangSuntornnond, RatimaHu, GuokuSupuran, ClaudiuHu, GuoliSurguchov, AndreiHu, HaoSurleva, AndrianaHu, JiafenSurmacki, Jakub MaciejHu, JinmingSuryanarayana, PhanishHu, MinSut, StefaniaHu, QinhongSutherland, Hamish S.Hu, Shang-HsiuSutton, Adam T.Hu, WanheSuzuka, ToshimasaHu, Wei-PingSuzuki, HideoHu, Xiang-PingSuzuki, MaikoHu, XiaoqiangSuzuki, RyuichiroHu, XiaoyuSuzuki, ToshikazuHu, YanmeiSuzuki, ToyofumiHuang, AnpengSuzutani, TatsuoHuang, Cheng-LiangSuzzi, GiovannaHuang, Chia-HuaSvavarsson, Halldor GudfinnurHuang, Chi-ChangSvete, JurijHuang, Chih-ChiaSvetlana, Tretsiakova-McNallyHuang, Chih-FengSvetlov, MaksimHuang, FaqingSvoboda, LadislavHuang, Genin GarySvoronos, Paris D. N.Huang, Haw-MingSwairjo, Manal A.Huang, Hui-ChiSwarbrick, CrystallHuang, HungJiSwartz, Talia H.Huang, Jiann-JyhSwatko-Ossor, MartaHuang, Jui-HsienSweeney, ElizabethHuang, Meng-YuanŚwiątek, ŁukaszHuang, PengŚwiątek, PiotrHuang, RenaŚwiderek, KatarzynaHuang, RimingSwidergall, MarcHuang, TimothySwiecilo, AgataHuang, Tse-HungŚwiergosz, TomaszHuang, WeiminŚwietlicka, IzabelaHuang, WeixinŚwietlik, RomanHuang, Wen-ChungŚwiętnicki, WiesławHuang, XiaodanSwiezewska, EwaHuang, XuefengSwift, ThomasHuáng, XuriSwinarew, AndrzejHuang, Ya-lingSychrovsky, VladimirHuang, Yeou-LihSydnes, Leiv K.Huang, YifengSyguda, AnnaHuang, Yu-ChunSykłowska-Baranek, KatarzynaHuang, Yu-FangSykora, DavidHubbe, MartinSýkora, JanHubbe, Martin A.Sylla, MaitéHuber, GaspardSymianakis, EmmanouilHuber, Roland G.Syrovets, TatianaHubicka, UrszulaSys, MilanHübner, FlorianSytar, OksanaHuc, VincentSytykiewicz, HubertHuczyński, AdamSzabelski, PawełHudalla, Gregory A.Szabo, AndrasHudson, RobertSzabó, LászlóHudson, SamuelSzabo, MilanHuebschle, Christian BertramSzacon, ElzbietaHuefner, AntjeSzafert, SlawomirHuerta, MiguelSzafranek-Nakonieczna, AnnaHuertas, Jesús R.Szafraniec-Szczęsny, JoannaHuh, Joo YoungSzafrańska, KatarzynaHuh, SeongSzakiel, AnnaHuisman, MarcSzakonyi, ZsoltHułas-Stasiak, MonikaSzala, MateuszHumar, MihaSzaleniec, MaciejHung, AndrewSzállási, ÁrpádHung, Hsin-YiSzałowski, KarolHung, Shih-YaSzanto, MagdolnaHungerford, JamesSzatmári, TündeHungerford, NatashaSzczepański, PiotrHunter, LukeSzczubialka, KrzysztofHunter, Robert P.Szebenyi, MarianHuntosova, VeronikaSzéchenyi, AleksandarHuq, Md. AmdadulSzécsi, MihályHurren, ChristopherSzeiffova Bacova, BarbaraHurtado Oliva, Miguel ÁngelSzekely, GyorgyHusnjak, KoraljkaSzeleszczuk, ŁukaszHussain, HidayatSzendrő, KatalinHussein, AhmedSzeto, Savio Yim TongHussein, WaleedSzewczyk, AgnieszkaHusson, JeromeSzewczyk, KatarzynaHvattum Divon, HegeSzewczyk, RomanHwang, Bang YeonSzewczyk-Golec, KarolinaHwang, Eun SookSzidarovszky, TamásHwang, In KooSzilvási, TiborHwang, Jiun-RenSzlachcic, AnnaHwang, Kwon TackSzlęk, JakubHwang, Long-ChihSzoboszlai, NorbertHwangbo, CheolSzoka, ŁukaszHwu, Jih RuSzöllösi, GyörgyHyeyoung, SeoSzolnoky, GyozoHyun, Sang-HwanSzoor, ArpadIacumin, PaolaSzopa, AgnieszkaIadarola, PaoloSzopka, KatarzynaIammarino, MarcoSzostak, MichalIanchis, RalucaSzostak, RomanIatrou, HermisSzpyrka, EwaIatsunskyi, IgorSztanke, KrzysztofIbáñez Escribano, AlexandraSztanke, MałgorzataIbáñez, Pablo Rafael PardoSzubert, KarolIbrahim, Mohamed AliSzukalski, AdamIbrahim, SalamSzulc, KarolinaIchiishi, NaokoSzulczyk, DanielIchikawa, SatoshiSzultka-Młyńska, MałgorzataIdowu, TemiloluSzumny, AntoniIelciu, IrinaSzumski, MichalIentile, RiccardoSzwajca, AnnaIgea, AnaSzweda, PiotrIglesias, Alberto Á.Szwengiel, ArturIglesias, EmiliaSzyja, Bartłomiej M.Ignat, MariaSzymandera-Buszka, KrystynaIgnatov, AlexanderSzymańki, PawełIgne, BenoîtSzymanska-Chargot, MonikaIgnés-Mullol, JordiSzymborski, TomaszIgnjatovic, NenadTa, HangIizuka, KatsumiTabacaru, AurelIkechukwu, EjidikeTabanca, NurhayatIkeda, MasatoTabassum, ShawanaIkeda, MasayukiTabata, YasuhikoIkegami, TakahisaTabatabai, HabibIkonomopoulou, MariaTabernero, VanessaIkura, TeikichiTabernero-Duque, María-JesúsIkuta, NaokoTada, KoheiIl’yushin, Mikhail A.Taddei, MarcoIlaš, JanezTaddei, MaurizioIlia, GheorgheTadić, VanjaIlic, NebojsaTadyszak, KrzysztofIlic-Stojanovic, SnezanaTaffa, Dereje HailuIlinskiy, YuriyTafuri, SimonaIlisz, IstvanTaga, YukiIlisz, IstvánTagad, HarichandraIlla, OnaTagad, Harichandra D.Illescas, JavierTagit, OyaIm, HyungsoonTagliatesta, PietroImai, HirooTagliazucchi, DavideImai, JunTaglienti, AnnaImam, SyedTaglietti, Angelo MariaImamoto, YasushiTago, KenjiImamura, MasahiroTaguchi, ToruImberti, CinziaTaha, MuhammadImhof, PetraTahara, HironobuImlach, Wendy L.Tai, Dar-FuImm, Jee-YoungTaira, JunseiImoto, KentaTait, Steven L.Imperatore, ConcettaTajti, ÁdámImprota, RobertoTakada, AkihikoImre, SilviaTakafuji, MakotoInaba, HiroshiTakahashi, FumikiInagaki, HidetoshiTakahashi, KaitoInchingolo, FrancescoTakahashi, KazuoIngallina, CinziaTakahashi, KenIngelman-Sundberg, MagnusTakahashi, MasakiInglese, PaoloTakahashi, NobuakiIngram, ConradTakahashi, OsamuInguimbert, NicolasTakai, ChikaInokuchi, TsutomuTakanami, ToshikatsuInokuma, TsubasaTakase, HidekiInomata, HiroshiTakashima, EizoInomata, KatsuhiroTakasu, KiyoseiInoue, AtsukoTakatori, KazuhikoInoue, HirofumiTakaya, TomohisaInoue, KeiichiTakayoshi, SuzukiInui, MasayukiTakayuki, TsukuiInuzuka, ToshiyasuTakeda, YouheiInyushin, Mikhail Y.Takegahara, NorikoIoannides, TheophilosTakenaga, MitsukoIon, Rodica-MarianaTakenaka, ShigeoriIonescu, Elena RodicaTakeoka, ShinjiIonescu, Mihaela IleanaTakeshita, FumitakaIonete, Roxana ElenaTakeuchi, HideyukiIonita, PetreTakizawa, ShinobuIonut, Ioana A.Talhi, OualidIordache, FlorinTaliani, SabrinaIorizzi, MariaTalimian, AliIorizzo, MassimoTallawi, MarwaIppoliti, ThomasTalora, ClaudioIqbal, Hafiz M. N.Taly, AntoineIram, SurtajTam, Eric Kwok WaiIrazoqui, JavierTam, Roger Y.Iriepa Canalda, IsabelTamaian, RaduIrina, CarlescuTamainot-Telto, ZacharieIriondo, AmaiaTambe, MitaliIrwin, David C.Tambuwala, MurtazaIsakova-Sivak, IrinaTampa, MirceaIsaza Martínez, José HipólitoTampieri, FrancescoIseppi, RamonaTamulevičienė, AstaIshihara, KazuakiTamura, AtsushiIshihara, OsamuTamura, OsamuIshii, DaisukeTan, AngelIshii, TakahiroTan, Bee K.Ishikawa, RyutaTan, Bee KangIshimizu, TakeshiTan, ChenIshitsuka, YoichiTan, ChengxiaIshizuka, TakumiTan, Joanne T.M.Islam, Md SorifulTan, Lik TongIslam, ZohorulTan, Ming-QianIslas, LeonTan, Swee ChingIslas-Jácome, AlejandroTan, YanglanIsmael, Maria IsabelTan, Yaw SingIsman, Murray B.Tan, YingIsoda, KyosukeTan, ZhijianIsono, TakuyaTanabe, MakotoIsvoran, AdrianaTanabe, ShihoriItakura, YokoTanabe, YooItatani, YoshiroTanaka, EijiIticescu, CatalinaTanaka, HideoIto, MichihoTanaka, KazuoIto, NaokiTanaka, MasashiIto, ShosukeTanaka, MasatoIto, TakuyaTanaka, NaonobuIto, YoichiroTanaka, NobuyukiItoh, ToshiyukiTanaka, RyoItoh, YoshifumiTanaka, SatoshiItsuno, ShinichiTanaka, ShigenoriIturriaga-Vasquez, PatricioTanaka, TakayukiIván, BélaTanaka, TakujiIvančić Šantek, MirelaTanaka, TeruyoshiIvanov, SergeyTanaka, TomonariIvanov, Yuri D.Tanaka, YoshiyukiIvanova, Alla V.Tanaka, YujiIvanova, Donika G.Tanase, CorneliuIvanova, SvetlanaTanc, MuhammetIvashchenko, OlenaTančinová, DanaIwai, AtsushiTáncsics, AndrásIwaoka, MichioTandlich, RomanIwata, YukiyoshiTang, Chih-HsinIzaguirre, GonzaloTang, DamuIzumi, KoujiTang, HuaIzumi, YoshihiroTang, KailinIzzo, IreneTang, KeJ Edagwa, BensonTang, LongtengJabin, IvanTang, NaipengJablan, JasnaTang, Ren-ChengJablonska, KarolinaTang, Ri-YuanJabłońska-Trypuć, AgataTang, WeiweiJablonski, MiroslawTang, XinjingJabraoui, HichamTang, YaoJacak, JaroslawTang, Ye-FengJacevic, VesnaTang, YouhongJacinto, EstelaTang, You-ZhiJackai, Louis E. N.Tangadanchu, Vijai Kumar ReddyJackowski, KarolTani, FumitoJackson, Robert A.Taniguchi, MakotoJackson, William F.Taniguchi, MoyuJacob, ChristopheTaniguchi, NobukazuJacob, GopasTaniguchi, TsuyoshiJacob, PaulTanner, EdenJacobo-Velazquez, DanielTanzi, Maria CristinaJacobsen, ElisabethTao, LeiJacomin, Anne-ClaireTao, LimingJacquemin, DenisTao, YongshengJadeja, RavirajsinhTao, YunwenJaffrezic-Renault, NicoleTaoukis, PetrosJaganjac, MoranaTapia, Alejandro A.Jager, Martine JTappan, Bryce C.Jagerovic, NadineTappia, ParamjitJagminas, ArunasTarabanko, NikolayJain, AnkitTarabanko, Valery E.Jain, GauravTarallo, OresteJaiswal, Jagdish KumarTaranto, Alex GutterresJaja, Ishmael FestusTarasiuk, JolantaJakas, AndrejaTarasov, AndreiJaki, BirgitTard, CédricJakić, MićeTarkowská, DanušeJakimovski, DejanTarkowski, PetrJakobek, LidijaTarozzi, AndreaJakobusic Brala, CvijetaTarrago, LionelJakopic, JernejaTartaglia, AngelaJakovac, HrvojeTashima, Alexandre K.Jakovljevic, MartinaTashima, ToshihikoJakubczyk, AnnaTashiro, MitsuruJakubczyk, EwaTasic, LjubicaJakubowicz-Gil, JoannaTasinato, NicolaJakubowski, WitoldTasso, BrunoJalil, Abbe Maleyki MhdTata, Rama RaoJallet, DenisTateishi, KensukeJames, JoeTatikolov, Aleksander S.James, MargaretTatsuhiro, KojimaJames, Nicholas G.Tatsuya, KatoJameson, DavidTatullo, MarcoJamin, NadègeTaurin, SebastienJampilek, JosefTava, AldoJamróz, EwelinaTavakoli, JavadJamting, AsaTavani, CinziaJamwal, RohitashTavanti, FrancescoJana, AtanuTavares, José CarlosJana, NavenduTavares, Josean FechineJanda, ElzbietaTavladoraki, ParaskeviJanda, KatarzynaTawfike, AhmedJandl, KatharinaTayebati, Seyed KhosrowJanecek, StefanTayel, AmrJanecka, AnnaTaylor, Erika A.Janecki, DanielTchertanov, LubaJanecki, TomaszTchetina, Elena V.Janek, TomaszTchoukov, PlamenJänes, AlarTecilla, PaoloJanes, TrevorTecpoyotl-Torres, MargaritaJang, Dae-sikTedeschi, PaolaJang, Hae WonTedesco, IdoloJang, Sung-WukTeghil, RobertoJangampalli Adi, PradeepkiranTegl, GregorJaniak, MichalTeif, VladimirJanik, IreneuszTeixeira, AlfredoJanin, Yves L.Teixeira, Hellen S.Janissen, RichardTeixeira, João M. C.Janiszewska-Turak, EmiliaTeixeira, Luís FilipeJanjetovic, ZoricaTeixeira, NatérciaJankauskaite, VirginijaTeixeira, RitaJankauskienė, JurgaTeixeira, RóbsonJankowska, ElzbietaTeixeira-Santos, RitaJanning, MelanieTeixidó, JordiJànos, MartonTejero, JesúsJanovák, LászlóTekko, IsmaielJanowiak, Blythe E.Telang, SuchetaJanuchowski, RadosławTéletchéa, StéphaneJanuszewski, RafałTellez Jurado, LuciaJanzen, AnnekeTéllez-Téllez, MauraJanzen, Daron E.Téllez-Valencia, AlfredoJaparidze, AleksandreTemesvari, LeslyJara Palacios, María JoséTemussi, Piero A.Jara, PaulTeng, Ba-BieJaradat, NidalTeng, PengJarak, IvanaTenhaeff, WyattJarczewski, SebastianTeo, JeremyJaren, CarmenTeobald, KupkaJarocka-Karpowicz, IwonaTeodoro, JoãoJarończyk, MałgorzataTera, MasayukiJaros, DorisTeramoto, HidetoshiJarosz-Wilkołazka, AnnaTeramoto, NaozumiJarzabek, DariuszTeramoto, YoshikuniJarzebski, MaciejTerec, AnamariaJasinski, MarcinTerent’ev, Alexander O.Jasinski, RadomirTerentjeva, MargaritaJasinski, RadoslawTerenzi, AlessioJasiurkowska-Delaporte, MałgorzataTerhorst, CoxJaśkowska, JolantaTermolino, PasqualeJastrzab, RenataTermopoli, VeronicaJastrzebska, BeataTernesten-Hasséus, EwaJastrzebska, IzabellaTerpos, EvangelosJasutiene, InaTerpou, AntoniaJatkowska, NataliaTerraneo, GiancarloJaudzems, KristapsTerrasson, VincentJaumot, JoaquimTerrinoni, AlessandroJay, StevenTeschke, RolfJayaprakash, PriyamvadaTesic, ZivoslavJazvinšćak Jembrek, MajaTeslic, NemanjaJaźwiński, JarosławTetsuo, OkadaJean, LudovicTeusch, NicoleJeandet, PhilippeTews, DanielJedelská, JarmilaTexido Bartes, RobertJedrejek, DariuszTezuka, YasuhiroJędrzejczyk, Roman J.Thakur, AshutoshJedziniak, PiotrThakur, SachinJee, JunGooThakur, VijayJee, Jun-PilThakur, Vijay KumarJeffrey, KieftThalhammer, GregorJekle, MarioThangaraj, AnnaduraiJeldres, Ricardo I.Thapa, ManojJeleń, HenrykThapa, SurendraJeleń, Henryk H.Theodorakis, Emmanuel A.Jeleń, MałgorzataTheodossiou, Theodossis A.Jelińska, AnnaTheoduloz, CristinaJelliss, PaulThi, Thai Thanh HoangJelokhani-Niaraki, MasoudThiebault, ThomasJelonek, KatarzynaThiel, GeraldJelsch, ChristianThies, StephanJena, Himanshu SekharThiriet-Rupert, StanislasJenett-Siems, KristinaThives, Liseane PadilhaJenkins, IanThomas, AnanyaJenny, Titus A.Thomas, Eric JimJensen, FrankThomas, JohnJensen, Lars RThomas, MaciejJensen, MikaelThomas, Morgan L.Jensen, Svend BorupThomas, Robert K.Jenssen, HåvardThomas, Stephen P.Jeon, JonghoThomas, ThresiaJeon, OjuThomas, TiffanyJeon, Sung HoThomas, WayneJeong, EuikyoungThompson, Miles D.Jeong, Jae MinThompson, RoneyJeong, Keun-YeongThompson, StephenJeong, Lak ShinThomson, JenniferJeong, Nak CheonThorpe, ClareJeong, Seong HoonThybring, Emil EngelundJeong, Yeon-junTiago, GonçaloJeremić, SanjaTian, Chun-JieJerez, NancyTian, FurongJerković, IgorTian, GuocaiJerkowić, IgorTian, JiangweiJermakowicz-Bartkowiak, DorotaTian, Kun VivianaJeromel, AnaTian, LiJerzykiewicz, LucjanTian, Shung WuJeschek, MarkusTian, WeiguoJesionek, MarcinTian, YeJesson, DavidTiano, LucaJeucken, AikeTibullo, DanieleJezierska-Mazzarello, AnetaTidgewell, KevinJi, GuozhaoTietze, DanielJi, PengTikhonov, VladimirJi, PengfeiTikhonova, IrinaJi, ShaofeiTimoshenko, Alexander V.Ji, TengfeiTimson, David J.Ji, TianjiaoTing, Richard C. H.Ji, XianglingTintaru, AuraJia, LiTipeev, AzatJia, ShangTiperciuc, BrindusaJia, TonyTirado, Diego F.Jia, YanweiTircso, GyulaJia, ZheTirillini, BrunoJiang, FuyiTiritan, Maria ElizabethJiang, JianxiongTiriveedhi, VenkataswarupJiang, JingTischler, DirkJiang, JiuxingTisovský, PavolJiang, NanTitov, Aleksei A.Jiang, XuefengTiwari, Rakesh K.Jiang, XuntianTiyyagura, Hanuma ReddyJiang, ZaixingTjandra, NicoJiang, ZhenTkac, JanJiang, ZiwenTlili, AnisJiao, KuiTo, Ching TatJibran, RubinaTobiszewski, MarekJijie, RoxanaTobrman, TomášJimbo, MitsuruTocmo, RestitutoJiménez Arellanes, AdelinaTodasca, Maria-CristinaJiménez Carvelo, Ana MaríaTodde, SergioJimenez Migallón, AlfonsoTodorović, Vanja M.Jiménez, Jordi JuárezToepel, JörgJiménez-Araujo, AnaTofana, MariaJiménez-Aspee, FelipeTofani, DanielaJiménez-Barbero, JesusToffolo, MichaelJiménez-Jiménez, Félix JavierToillon, Robert-AlainJiménez-Morillo, Nicasio TomásToiu, AncaJiménez-Tenorio, ManuelTojo, TomohiroJimeno, CirilTokarz, BarbaraJimi, EijiroTokarz-Sobieraj, RenataJin Cheol, KimTokmina-Lukaszewska, MonikaJin, CongruiToledo-Neira, CarlaJin, FangTolga, KarsiliJin, Guo-XinTolgyessy, PeterJin, JiefuTolkou, AthanasiaJin, MinTolomelli, AlessandraJin, Ning-deTolosa, EzequielJin-chul, KimToma, LucioJindřich, JindřichTomai, PierpaoloJing, HuiTomanek, BoguslawJios, Jorge L.Tomás-Barberán, Francisco A.Jiri, PospisilTomasello, BarbaraJirkovsky, EduardTomasevic, IgorJishi, RadiTomasik, PiotrJo, HyunilTomasini, ClaudiaJobe, TimothyTomášková, JanaJobic, StephaneTomaszewska, EwaJochum, ChristophTomaz, IvanaJodłowski, GrzegorzTombelli, SaraJohannissen, Linus O.Tomczyk, WojciechJohn Jervis, PeterTomé, Augusto C.John, ŁukaszTomek, PetrJohn, RohanTomer, Vijay K.Johnson, AaronTomi, FélixJohnson, ChristopherTomić, SimonidaJohnson, ElizabethTomoda, HiroshiJohnson, JoshuaTomonaga, ShozoJohnson, Samuel A.Tomuta, IoanJohnson, Shelby L.Tonazzi, AnnamariaJohnsson, MatsTondi, DonatellaJohnston, Linda J.Tonelli, MicheleJohnston, Michael J. W.Tong, ShengqiangJohnstone, JeanetteTong, WenmingJokić, StelaTong, YexiangJonathan B., CooperTönges, LarsJones, Christopher E.Tonolo, GiancarloJones, MarjorieTonutti, PietroJonnalagadda, SreekanthTopoglidis, EmmanuelJoó, FerencToprakci, Hatice Aylin KarahanJoo, Soo-HyunTopuz, FuatJoos, JonasToriba, TaiyoJordá, José LuisToribio, LauraJordão, António ManuelTorigoe, KanjiroJordheim, MonicaTorikai, KoheiJørgensen, BodilTormo, JoseJørgensen, Kåre B.Torneiro, MercedesJorrin-Novo, Jesus V.Tornesello, Anna LuciaJose, JineyToro, AyelénJoshi, Pushpa RajToro, Roberta G.Joshi-Navare, KasturiTorok, MariannaJovanović, AleksandarToropov, AndreyJovanovic, ZivkoTorović, LjiljaJoven, JorgeTorre, Maria LuisaJoyce, PaulTorre, RenatoJozic, IvanTorregiani, ElisabettaJóźwicki, WojciechTorres Hernández, YadirJuan, Maria EmiliaTorres Perez, María DoloresJuárez-Maldonado, AntonioTorres, Adrian GabrielJudeh, ZaherTorres, SofiaJudeinstein, PatrickTorres-Giner, SergioJuhasz, BelaTorres-Padrón, María EstherJulander, Justin G.Torres-Ramírez, EduardoJulian, FernandoTorri, LuisaJulien, Christian M.Torricelli, PaolaJulien, PierreTorrico, Damir D.Jung De Andrade, MônicaTorroba, TomasJung, Byung JunTorzewska, AgnieszkaJung, Ho-SupTosa, Monica IoanaJung, Hye JinTosha, TakehikoJung, HyunsookTosheva, LubomiraJung, Ji HoonToshio, KoizumiJung, JooheeTošner, ZdeněkJung, Min-CherlTothova, CsillaJung, SeunhoToti, KiranJung, Sokhee PhilemonTotsingan, FilbertJung, Tae SungTotti, FedericoJung, Woo-JinTõugu, VelloJung, Yong ChaeTouraud, DidierJung, Young SungTournié, AurélieJungblut, Peter R.Toušek, JaromírJunichi, KitanakaToyama, IkuoJunior, EdeildoToyoda, YuJunkar, ItaToyohara, JunJuodkazis, SauliusTőzsér, JózsefJuranović Cindrić, IvaTräbert, ElmarJurasekova, ZuzanaTrabocchi, AndreaJurca, TitelTraboni, SerenaJurczak, JanuszTrader, DarciJurić, SlavenTrafiałek, JoannaJurij, TronteljTrajkovic, VladimirJurikova, TundeTrammell, SamuelJurkonienė, SigitaTrammell, Scott A.Jurkowska, HalinaTran, TrongJurman, GiuseppeTrandafirescu, Cristina-MariaJusticia, JoséTrani, AntonioJuszczak, LesławTranmer, GeoffJyothi, Rajesh KumarTrapani, AdrianaK. Friestad, GregoryTrapella, ClaudioK. Karabagias, IoannisTratnik, Janja SnojKaas, QuentinTravlos, IliasKabelác, MartinTrcek, JanjaKabir, AbuzarTrdan, StanislavKačániová, MiroslavaTreebak, JonasKachrimanis, KyriakosTremonte, PatrizioKącka-Zych, AgnieszkaTrequattrini, FrancescoKaczmarek, BeataTres, Marcus ViniciusKaczmarek, MariuszTretyakov, Evgeny V.Kaczmarek-Kędziera, AnnaTrevaskis, NatalieKaczor, Agnieszka A.Trevisan, AndreaKaddour, HusseinTriantafyllidis, KonstantinosKadela-Tomanek, MonikaTribello, GarethKaden, MarikaTricot, GregoryKadlubowski, SlawomirTrifonov, RostislavKadokawa, Jun-ichiTrifonova, Oxana P.Kadota, IsaoTrigger, SergeyKafarski, PawelTrilleras, JorgeKafouris, DemetrisTrimigno, AlessiaKageyama, HakutoTrincone, AntonioKagota, SatomiTrindle, CarlKahlert, StefanTrinh, ThuatKairys, VisvaldasTriola, GemmaKairytė, AgnėTripathi, Jaindra NathKaisarly, DaliaTripathi, ManishaKaiser, AnnetteTripodi, GianlucaKaiser, FabianTripodo, GiuseppeKaji, HiroshiTrippier, Paul C.Kajimoto, ShinjiTritschler, LaurentKajitani, TakashiTrivanovic, DrenkaKajiwara, TakashiTrofimova, MayaKajiyama, SatoshiTrögl, JosefKálai, TamasTrognitz, FriederikeKalantar-zadeh, KouroshTrojanowicz, MarekKalas, WojciechTrombini, ClaudioKalayda, GannaTroncoso, Ana MaríaKalhapure, Rahul S.Tropea, AlessiaKalíková, KvětaTroppmair, JakobKalina, JanTrotsko, NazarKalinich, JohnTrovato, FabioKalinin, VladimirTrubetskaya, AnnaKalinowska Lis, UrszulaTrucillo, PaoloKalinowska, MonikaTrujillo, Antonio-JoséKalinski, Jarmo-Charles J.Trujillo, CristinaKalisz, StanisławTrujillo-Cayado, LuisKaliva, MariaTrujillo-Rodríguez, María J.Kaliyan, NalladuraiTrujillo-Rodríguez, María JoséKalkum, MarkusTrumble, John T.Kallithraka, StamatinaTruong, Vi-KhanhKallitsis, Joannis K.Trushkov, Igor V.Kalló, GergőTrusso Sfrazzetto, GiuseppeKallscheuer, NicolaiTrusso, SebastianoKalluru, PolirajuTrybuła, ZbigniewKalmár, JózsefTrzaskowski, BartoszKalogianni, DespinaTrzcińska, MonikaKalogiouri, NatasaTrzeciak, AnnaKalogirou, AndreasTsai, Chen-YenKaloyanova, Dora V.Tsai, Hui-LienKalušević, AnaTsai, Jen-ChiehKalwat, Michael A.Tsai, Pi-JenKamal, Fadia AliTsai, Shu-HuaiKamali, SaeedTsai, Shu-YaoKamanina, NataliaTsai, Wan-YuKamath, DivyaTsakalof, AndreasKamenov, GeorgeTsakovska, IvankaKameo, HajimeTsaltas, DimitrisKamgang Nzekoue, FranksTsang, MichaelKamiloglu, SenemTsantili-Kakoulidou, AnnaKaminska, BozenaTsarkova, LarisaKamińska, JoannaTsatsakis, AristidisKaminski, Daniel M.Tsatsanis, ChristosKaminski, KamilTscheliessing, RupertKamiński, KrzysztofTse, William K.F.Kaminsky, WernerTse-Dinh, Yuk-ChingKamm, BirgitTseng, Chih-HuaKamysz, ElżbietaTseng, Wei-LungKan, Chi-waiTsentalovich, Yuri P.Kanada, MasamitsuTserepi, AngelikiKanai, MasashiTsiafoulis, Constantinos G.Kananovich, Dzmitry G.Tsibouklis, JohnKanaori, KenjiTsimidou, MariaKanapitsas, AthanasiosTsimpanogiannis, Ioannis N.Kanda, NaokoTsirelson, Vladimir G.Kandathil, Shaun M.Tsirka, KyriakiKandil, SaharTsironi, TheofaniaKandylis, PanagiotisTsitsilianis, ConstantinosKaneko, GenTsitsilonis, Ourania E.Kanematsu, HideyukiTsivileva, OlgaKang, ChenTskhovrebov, Alexander G.Kang, ChulhunTsopelas, FotisKang, Hee EunTsopmo, ApollinaireKang, JianmingTsotsalas, ManuelKang, Jun-GillTsoupras, AlexandrosKang, Ju-SeopTsubaki, KazunoriKang, Kyo BinTsuchida, SachioKang, KyungsuTsuchiya, HiroyukiKang, KyungtaeTsuji, Atsushi B.Kang, MinjeeTsuji, HidetoKang, Nam SookTsukamoto, TakashiKang, Sang SunTsumuraya, TakeshiKang, Sun ChulTsuneda, TakaoKang, WonkuTsunedomi, RyouichiKang, Yang JunTsunoda, MakotoKang, Young-HeeTsurkan, Mikhail V.Kang, YunqingTsutsumi, HiroshiKangas, HeliTsutsumi, OsamuKangas, TeijaTsvetkov, NikolaiKani, KianTsyganov, Viktor E.Kania, HenrykTu, Jun-LingKano, NaokiTu, MinKant, SashiTu, ZhengkaiKantminienė, KristinaTubaro, CristinaKantzas, ApostolosTubaro, FrancoKao, Chai-LinTubić, AleksandraKao, Cheng-YenTuccinardi, TizianoKao, PeterTuci, GiuliaKao, Shao-HsuanTucker, Thomas J.Kaplan, BarbaraTudoroiu, NicolaeKappel, NoemiTufa, Ramato AshuKappenstein, CharlesTufariello, MariaKapusta, IreneuszTufariello, MiriamKar, SupratikTullio, VivianKarabagias, IoannisTullius Scotti, MarcusKarabaliev, MiroslavTůma, KarelKaragiannis, TomTůma, ZdeněkKarahan, H. EnisTumova, LenkaKarakashev, StoyanTun, Mya Myat NgweKarakoti, AjayTundis, RosaKaram, SherifTunesi, MartaKaraman, MajaTungmunnithum, DuangjaiKaraman, RafikTunick, MichaelKaramanoli, AikateriniTunon, InakiKaramaouna, FilitsaTuovinen, OlliKarasali, HelenTuranek, JaroslavKarbownik, AgnieszkaTurano, PaolaKarcz, DariuszTurck, Christoph W.Karczmarzyk, ZbigniewTurco, AntonioKardassis, DimitrisTurco, RosaKareiva, AivarasTurhanen, PetriKarelson, MatiTurnaturi, RitaKarikas, George-AlbertTurnbull, Matthew W.Karim, NasiaraTurner, Alice M.Kariyat, RupeshTurner, DavidKårlund, AnnaTurowski, Tomasz W.Karnati, Konda ReddyTurrini, EleonoraKarolewicz, BożenaTurrini, FedericaKároly, LázárTurunen, LottaKarousou, EugeniaTutone, MarcoKarpe, Avinash V.Tuzimski, TomaszKarpichev, YevgenTvrdeic, AnteKarpinski, Tomasz M.Twarużek, MagdalenaKarra, LailaTyler, RobertKarvelas, EvangelosTylewicz, UrszulaKarwowska, MałgorzataTyliszczak, BożenaKarwowski, BoleslawTylkowski, BartoszKasapidou, EleniTymecki, ŁukaszKaseem, MosabTyski, StefanKaserer, TeresaTyystjärvi, EsaKashchenko, Nina I.Tzanavaras, Paraskevas D.Kashif, MuhammadTzeli, DemeterKashman, YoelTzen, Jason T.Kasiotis, KostasTzeng, Biing-ChiauKaškonienė, VilmaTzeng, Shiang-JongKasnatscheew, JohannesTzeng, Shu-LingKašparovský, TomášTzin, VeredKasperkiewicz, PaulinaTziveleka, Leto-AikateriniKasprowicz, DobrosławaTzortzakis, NikosKasprzak, ArturTzovenis, IoannisKaszowska, MartaTzvetkova, PavletaKataev, EvgenyUan, Jun-YenKatahira, RuiUbeda, CristinaKatalinić, MajaUbiali, DanielaKatanić, ZoranaUchida, SatoshiKataoka, HiroyukiUchida, YoshiakiKataoka, NaoyukiUchihashi, TakayukiKatarzyna, MalarzUchikoshi, MasahitoKatayama, TakeshiUchiyama, TaketoKathuria, HimanshuUdal’tsov, AlexanderKatikou, PanagiotaUddin, JamalKatin, KonstantinUdrescu, LucretiaKato, EisukeUehara, TomoyaKato, FumihiroUekusa, HidehiroKato, KeisukeUeno, MikinoriKato, KoichiUetani, KojiroKato, TakamitsuUetrecht, CharlotteKatsafadou, AngelikiUetz, PeterKatselis, GeorgeUfnal, MarcinKatsila, TheodoraUivarosi, ValentinaKatsuyama, YoheiUkalska-Jaruga, AleksandraKattamuri, PadmanabhaUllah, IrfanKatunin, AndrzejUmehara, MikihisaKauppinen, AnuUmerska, AnitaKauppinen, Esko I.Ungurianu, AncaKaur, AmandeepUnković, NikolaKaur, GunveenUnno, KeikoKaushik, Nagendra KumarUno, TomohideKavan, LadislavUnsworth, WillKavita, KumariUpdegrove, Taylor B.Kawabata, KyuichiUrakawa, OsamuKawaguchi, SeigouUrban, MilanKawaguchi, Shin-ichiUrban, PhilippeKawai, KiyohikoUrbańczyk-Lipkowska, ZofiaKawai, TakayukiUrbanek, PavelKawakami, AkinoriUrbaniak, AlicjaKawakami, JunUrra, Félix A.Kawakami, SusumuUrriolabeitia, EstebanKawakami, YukiUrso, ElenaKawakita, HidetakaUrzi, ClaraKawamura, FumioUsai, DonatellaKawamura, IzuruUsai, MariannaKawanabe, HiroshiUsama, Syed MuhammadKawano, Daniel FábioUsami, YoshihideKawato, TakayukiUshijima, KentaroKawiak, AnnaUssowicz, MarekKayaki, YoshihitoUsui, KenjiKayano, Shin-ichiUsuki, ToyonobuKazakova, Oxana B.Usuki, YoshinosukeKazimierski, RadosławUtikar, Ranjeet P.Kazokaite, JustinaUusi-Oukari, MikkoKazuhiro, ShikinakaUversky, VladimirKebukawa, YokoUziel, OritKeenan, JoanneV. Likhanova, NatalyaKeglevich, GyörgyVaculovičová, MarketaKehr, Nermin SedaVafidis, DimitrisKelarakis, AntoniosVaghasiya, JayrajKele, PeterVagin, MikhailKellenberger, StephanVago, RiccardoKeller, LenaVahabi, HenriKellner, StefanieVaidyanathan, GanesanKello, MartinVaidyanathan, SriramKellogg, Joshua J.Vakh, Christina S.Kem, William R.Vakros, JohnKenry, K.Valandro, SilvanoKerch, GarryValcarce, David G.Kerman, KaganValdersnes, StigKerr, WilliamVale, Carlos Alberto Garcia DoKerrigan, Nessan J.Vale, PauloKerth, AndreasValentao, PatriciaKertmen, AhmetValente, Artur J. M.Kerwin, Sean M.Valente, JoanaKesari, Kavindra KumarValente, LigiaKesharwani, SiddharthValentova, JindraKeshavarz, MeysamValentová, KateřinaKęska, PaulinaValenzuela, MiguelKetkar, AmitValenzuela, RodrigoKettler, RichardValero-Cases, EstefaníaKeyzers, RobValgimigli, LucaKeyzers, RobertValiante, VitoKhaddaj Mallat, RayanValitutti, FrancescoKhalifa, ShadenValiveti, ChaitanyaKhalili-Araghi, FatemehValkenier, HennieKhallouki, FaridValle, Luisa DallaKhalymbadzha, Igor A.Vallée, YannickKhambu, BilonVallejo, Javier P.Khan, AbdulValles, Soraya L.Khan, Dr. Md. ShakhaoathValles, VincentKhan, HamidullahValletta, AlessioKhan, Meraj AlamValsami, GeorgiaKhan, MerajuddinValyaev, Dmitry A.Khan, Mohammad ImranVamanu, EmanuelKhan, Mohammad SharifVan Aerschot, ArthurKhan, MujeebVan Bennekom, W.P.Khan, Omar F.Van Beusechem, VictorKhan, SajidVan Bommel, Maarten RKhan, Zafar K.Van Deenen, NicoleKhang, GilsonVan Den Berg, Marco AlexanderKharlampieva, EugeniaVan Den Ende, WimKhattab, TawfikVan Den Meiracker, Anton H.Khaw, Kooi YeongVan Der Kamp, Jan WillemKhaw, Kooi YepngVan Der Meer, Marcel T. J.Khdair, AdnanVan Der Merwe, MarieKhivantsev, KonstantinVan Der Vlugt, Jarl IvarKhlebnikov, Andrei I.van Duijn, BertKhmaladze, Alexander T.Van Dyke, MichaelKhmelinskii, IgorVan Hecke, KristofKhodadadi, Jay M.Van Herwijnen, HendrikusKhoder, MouhamadVan Hoecke, KarenKholmukhamedov, AndalebVan Horn, WadeKhong, AnthonyVan Lis, RobertKhoshnood, AbbasVan Mourik, TanjaKhotimchenko, Maxim Y.Van Noorden, Cornelis Johannes ForrindinisKhozin-Goldberg, InnaVan Ree, TeunisKhung, Yit LungVan Rijn, Richard M.Khurana, NamrataVan Steenberge, Paul Hm M.Khushwant Singh, BhullarVan Zyl, WernerKia, RezaVan, Thi Thu HaoKicel, AgnieszkaVance, DavidKichler, AntoineVande Velde, ChristopheKiciński, WojciechVanden Eynde, Jean JacquesKiec-Kononowicz, KatarzynaVandenbroeck, KoenKiel, JaapVanderlei, Maria De FátimaKieliszek, MarekVandooren, JenniferKierys, AgnieszkaVanella, LucaKierzek, KrzysztofVanin, Fernanda MariaKiesewetter, Dale O.Vannacci, AlfredoKijewska, MonikaVanni, EsterKikuchi, HaruhisaVannini, AndreaKikuchi, HidehikoVanthuyne, NicolasKikuchi, TakeshiVányolós, AttilaKikuta, ShingoVapnik, YevgenyKil, Kun-EekVaradarajan, ShankarKilbourn, Michael R.Váradi, JuditKilcawley, KieranVarani, LucaKilian, KrzysztofVarchi, GretaKilk, KalleVardhan, HarshKim, BongleeVarga, GáborKim, Byoung SuhkVarga, SzabolcsKim, Choon YoungVarga, ZoltanKim, Chuk YoungVargas Jentzsch, PaulKim, Dong SeonVarghese Gupta, SheebaKim, DukminVarma, Rajender S.Kim, Gue-HyunVarna, MarianaKim, Ha-NeuiVárnagy, KatalinKim, Hee-JoonVaroni, Elena MariaKim, Hoe JoonVarotto, SerenaKim, HojoongVarvounis, GeorgeKim, Hong-DuckVasas, AndreaKim, HoonVasco Vidal, AldrinKim, Hye KyongVasco-Calle, Diego AndrésKim, HyoncholVascotto, CarloKim, Hyun JoonVasdev, NeilKim, Hyun JungVasic, VesnaKim, HyungsikVasicek, OndrejKim, Hyun-JungVasile, Bogdan StefanKim, Hyun-SoonVasile, FrancescaKim, InKyeomVasilescu, AlinaKim, JaehanVasiliev, Mikhail M.Kim, JaehwanVasilyev, Aleksander V.Kim, JaheonVašková, JankaKim, JanghoVasos, PaulKim, Jeong-ChulVasquez, MarlenKim, Jeong-HwanVassallo, AntonioKim, Jeung GonVassilev, Nikolay G.Kim, JihoonVassilis J, InglezakisKim, JinhoVasu, DhananjayanKim, JinseokVatsadze, SergeyKim, Jong-KiVaughan, RogerKim, Jung-HoonVaughey, JohnKim, JungkwunVavitsas, KonstantinosKim, Ki HyunVayá Pérez, IgnacioKim, KwanghoVaz Junior, SilvioKim, Kyoung SubVaz, Daniela C.Kim, Kyung BoVaz, Josiana A.Kim, Kyung HoVaz, Pedro D.Kim, Kyung-MinVazquez Belda, BeatrizKim, Man-HoeVázquez Duhalt, RafaelKim, Mi-hyunVazquez Espinosa, MercedesKim, MinVazquez, Elba S.Kim, Min JungVázquez-Ovando, AlfredoKim, Min-CheolVázquez-Rodríguez, Gabriela A.Kim, Min-SooVázquez-Tato, M. PilarKim, Pyung-HwanVaz-Velho, ManuelaKim, Sang WanVecchio Ciprioti, StefanoKim, SanghoonVedernikov, Andrei N.Kim, Seok-HoVédrine, Jacques C.Kim, Seung-HyunVeenman, LeoKim, SeungjuVega Holm, MargaritaKim, SeyunVegvari, AkosKim, SokVehus, Tore SandnesKim, Soo UnVeit, GuidoKim, Soo-KyungVejpravova, JanaKim, Sung-HoonVejux, AnneKim, Sung-KunVek, ViljemKim, Tae HwanVekariya, RakeshKim, Tae KonVelasco, Rebeca HernandezKim, WoojinVelasco-Torrijos, TrinidadKim, YangVelena, AstridaKim, YongaeVelgosova, OksanaKim, YoungmeeVelikova, TsvetelinaKim, Young-SukVella, Filomena MonicaKim, Yu-JinVellecco, ValentinaKimbaris, AthanasiosVelmuzhov, AlexanderKimura, Ken-ichiVeltri, LuciaKimura, KiminoriVelu, Sadanandan E.Kimura, MasanariVemulapalli, VidyasiriKimura, TetsunariVendamme, RichardKinder, DavidVendrell, JosepKindl, MarijaVenerando, AndreaKing, Joan M.Venkatashamy Reddy, Mogalahalli VenkateshKing, Neil P.Venneri, FrancescaKingery, William L.Vennerstrom, JonathanKinzhalov, Mikhail A.Venskutonis, Petras RimantasKiokias, Sotirios N.Ventrella, DomenicoKippes, NestorVentruti, GennaroKirby, Christopher W.Ventura, Cinzia AnnaKirchmair, JohannesVenugopal, DhamodharanKirin, SreckoVenuti, ElisabettaKirsch, Gilbert H.Verbanac, DonatellaKirsch, Stefan F.Verde, IgnacioKirubakaran, PalaniVerdejo, BegoñaKirwan, JenniferVerdú-Andrés, JorgeKiryukhantsev-Korneev, Ph V.Veremeichik, Galina N.Kiselev, AntonVeres, MiklosKiselev, VitalyVerestiuc, LilianaKishida, MasaoVerga, DanielaKishigami, SatoshiVergara, DanieleKishimoto, JiroVergara, FreddKishimoto, ShinjiVergara, JoséKishimura, HidekiVerger, StéphaneKiskin, MikhailVerho, OscarKisková, TeréziaVerhoeven, AdrieKiss, EvaVerhoog, StefanKiss, RitaVeríssimo, MartaKitagawa, SeiichiVerly, Rodrigo MoreiraKitagawa, YuichiVermathen, MartinaKitagishi, HiroakiVermeulen, MarcKitajou, AyukoVernardou, DimitraKitamura, AkiraVerona, GuglielmoKitamura, YoshiakiVeronesi, FedericoKitano, YoshikazuVerotta, LuisellaKitney, StuartVerri, TizianoKiuru, PaulaVerri, Waldiceu A.Kiyama, RyoitiVervandier-Fasseur, DominiqueKiyokawa, EtsukoVerza, Simone GasparinKjaergaard, MagnusVesa, Stefan CristianKlajnert-Maculewicz, BarbaraVesey, DavidKlar, AgnesVeskoukis, Aristidis S.Klaus, AnitaVessieres, AnneKleczkowska, PatrycjaVetvicka, VaclavKleier, Daniel A.Veverka, VaclavKlein, AxelVeyron-Churlet, RomainKlein, Mark A.Vianello, RobertKlein, SusanneViapiana, AgnieszkaKlejdus, BorivojViappiani, CristianoKlem, KarelVicario, JavierKlempová, TatianaVicaș, Laura GrațielaKlepac, DamirVicente Crespo, GemmaKleszczyński, KonradVicente, Cristina P.Klimek-Ochab, MagdalenaVicente, Eduardo FestozoKline, PaulVicente, FranciscaKlingeler, RudigerVicente-Manzanares, MiguelKljusurić, Jasenka GajdošVícha, RobertKlochkov, Sergey G.Victor J., RicoKlochkov, Vladimir V.Victor, BrunoKlokov, DmitryVidal, FernandoKloskowski, TomaszVideau, PatrickKlouček, PavelVidic, JasminaKluczyk, AlicjaVidovic, BojanaKluczyk-Korch, KatarzynaVieillard, JulienKmita, AngelikaViejo, Claudia GonzalezKnapp, MichaelVielba, Jesús MaríaKnapp, SpencerViennet, ThibaultKnauth, PhilippeVierra, CraigKňazovická, VladimíraVigier, KarineKnez, DamijanVíglaský, ViktorKnežević, AnamarijaViglašová, EvaKnezevic, JelenaVigneresse, Jean LouisKniazeff, JulieVijayakumar, SarathKnight, ChristopherVijayan, MadhuvanthiKnittelfelder, OskarVijgenboom, ErikKnobloch, Thomas J.Viktorova, JitkaKnölker, Hans-JoachimVikulina, AnnaKnudsen, GabrielVil’, Vera A.Knupp, GerdVila, CarlosKnuschke, TorbenVila, Fernando D.Knutsen, ErikViladomat, FrancescKo, Horng-HueyVilaivan, TirayutKo, Sung-kyunVilanova, XavierKo, YounheeVilas, Jose LuisKobayashi, MotoyoshiVilela, AliceKobayashi, RikaVilgis, ThomasKobayashi, YayoiVilla, CarlaKobayashi, YuichiroVilla, ChiaraKobeissy, FirasVilla, CristianKober, KordVilla, StefaniaKobrak, Mark N.Villa-Morales, MaríaKobus-Cisowska, JoannaVillani, ClaudioKochmańska, AgnieszkaVillar, MarceloKocira, SławomirVillarreal, Vicent Sixte SafontKockmann, NorbertVillarreal-Gómez, Luis JesúsKodama, ShintaroVillaseñor, AlmaKodani, Sean D.Villaverde, Juan JoséKoech, PhillipVillegas, VictoriaKoel, MihkelVillena, JuanKoenig, Mary DawnVillorbina, GemmaKoff, AndrewViña, DoloresKoga, YoshikatsuViña, JaimeKoh, EunmiVinarov, ZahariKoh, Gar YeeViñas, ClaraKohanbash, GaryViñas, MiguelKöhl, GudrunVinauger, ClementKoikawa, MasayukiVincent, John B.Koike, HarukiVincent-Bonnieu, SebastienKoike, MariVincenzi, ColombinaKoivisto, JanneVincenzi, FabrizioKoizume, ShiroVincenzo, De LeoKojdecki, Marek AndrzejVincze, EvaKojima-Yuasa, AkikoVinet, RaúlKok, Victor C.Vinnik, DenisKokado, KentaVinodh, RajangamKokina, Tatyana E.Vinšová, JarmilaKokkarachedu, VaraprasadVinueza, NelsonKokkinakis, EmmanouilViola, GiampietroKokkola, TarjaViola, ManuelaKokoska, LadislavViola-Villegas, Nerissa ThereseKokotos, GeorgeVione, DavideKolackova, IvanaVirag, LaszloKolakowski, ZbigniewVirgili, FabioKolar, MitjaVirgili, TersillaKolaric, BrankoVirjamo, VirpiKolesińka, BeataVirto, MailoKolesnikova, Sophia A.Visconti, RobertaKolev, SvetoslavVisentin, AndreaKolling, StefanVisentin, FabianoKolmas, JoannaVisentin, SilviaKolniak-Ostek, JoannaVishe, MaheshKolobov, Alexandr AlexandrovichViškelis, JonasKolocouris, AntoniosViškelis, PranasKolodziejczyk, JoannaViskup, RichardKołodziejczyk, KrzysztofVismara, ElenaKolodziejski, Pawel AntoniViso Beronda, AlmaKolot, Mikhail N.Vistoli, GiulioKolukisaoglu, ÜnerVitale, FabrizioKomarnicka, Urszula K.Vitale, LucaKomatsu, SatoshiVitalini, SaraKomives, TamasVitasović Kosić, IvanaKomorowska, BeataViter, RomanKonar, ArkaprabhaVitetta, LuisKonar, SumitViva, Federico A.Konc, JanezVives, GuillaumeKończak, MagdalenaVivet, AlexandreKondo, JiroVizireanu, CameliaKondo, MizuhoVlachou, MarilenaKondo, Shin-ichiVladescu, MarianKonev, AlexanderVladić, JelenaKong, De-MingVladimirov, VladimirKong, De-XinVlainić, JosipaKong, DuanyangVlase, GabrielaKong, FangongVlasiou, ManosKong, L.X.Voća, NevenKong, LingyanVodeničarovová, MelitaKong, Won-SikVodyankina, OlgaKong, XianVögele, MartinKong, XiangruiVogt, SarahKonger, Raymond L.Vokurka, AlešKonieczny, PiotrVolcho, KonstantinKönig, GerhardVoliani, ValerioKönigs, Rene M.Volkova, Elena G.Konigsberg, William HVolmajer, JulijaKonishi, HiroakiVolodin, Alexander M.Konishi, MasaakiVomhof-DeKrey, EmilieKonjevoda, PaškoVon Eggeling, FerdinandKonno, HiroyukiVon Knethen, AndreasKonno, KatsuhiroVora, AnkitKonno, TomohiroVorobyeva, AnzhelikaKönönen, EijaVorontsov, AlexanderKononikhin, Alexey S.Vorsa, NicholiKonopka, IwonaVosloo, Hermannus C. M.Konrad, FutymaVostinaru, OliviuKonrad, LutzVostrikova, Kira E.Konstantinos, LitinasVoutsinas, Gerassimos E.Konstantinov, Spiro M.Voyiatzis, GeorgeKontek, BogdanVozzi, GiovanniKontogiannidou, EleniVrabel, MilanKontogiorgis, ChristosVračko, MarjanKontominas, Michael G.Vranes, MilanKontoni, Denise-Penelope N.Vranic, EdinaKontoravdi, CleoVriend, JerryKooistra, AlbertVrinceanu, NarcisaKopaczyńska, MartaVrontaki, EleniKopeć, AnetaVukojevic, KatarinaKopecký, VladimírVukomanovič, MariijaKopel, PavelVulic, Jelena J.Kopjar, MirelaVuorimaa-Laukkanen, ElinaKopjar, NevenkaVuthaluru, Hari BabuKopke De Aguiar, Jair AdrianoVyas, VijayKopra, KariVynios, DemitriosKopsahelis, NikolaosVyšniauskas, AurimasKopustinskiene, Dalia M.Vyviurska, OlgaKorach-André, MarionWaagbo, RuneKorb, MarcusWacher, María Del CarmenKorbecki, JanWaczulíková, IvetaKorchowiec, BeataWagner, AnikaKordyukova, LarisaWagner, PawelKordyum, ElizabethWahli, WalterKore, RajkumarWajs-Bonikowska, AnnaKorlyukov, Alexander A.Wakao, MasahiroKormos, AtillaWakioka, MasayukiKornhuber, Malte.Waksmundzka-Hajnos, MonikaKorolkov, Ilya V.Walach, WojciechKorolkova, YuliyaWałajtys-Rode, ElżbietaKoronkiewicz, StanisławaWalczak, KrzysztofKoroteev, Pavel S.Walczyński, KrzysztofKorshun, Vladimir A.Waldum, HelgeKorte, CarstenWałecka-Zacharska, EwaKortholt, ArjanWałęsa-Chorab, MonikaKoseva, NeliWalker, GregKosik-Bogacka, DanutaWalker, HeatherKosior, DominikWalker, Louise A.Kosma, PaulWall Medrano, AbrahamKosmala, MonikaWallace, David R.Košmerl, TatjanaWallace, Gregory Q.Košmrlj, JanezWallace, Heather M.Kosmulski, MarekWallace, KarlKošnář, ZdeněkWallace, M. ArielKosobucki, PrzemysławWalsby, CharlesKosova, NinaWalsh, KerryKostakis, GeorgeWalter, JeskeKostakis, IoannisWalton, JohnKostareva, AnnaWalwyn, Wendy M.Kostecka, MalgorzataWalz, JulianeKostecka-Gugala, AnnaWan, JiepingKostecki, MarekWanag, AgnieszkaKostevšek, NinaWang, Be-JenKostić, Aleksandar Ž.Wang, BoKostikov, Alexey P.Wang, CemingKostoulias, XeniaWang, ChangningKosyakov, Dmitry S.Wang, Chao-MinKoszegi, TamasWang, ChenguangKőszegi, TamásWang, Chi ChiuKoszelewski, DominikWang, Chih-ChiehKoszelnik, PiotrWang, Ching-ChiungKotek, JanWang, ChuanlongKotlyar, Alexander B.Wang, CongzhouKotlyar, Margarita V.Wang, DanhongKotora, MartinWang, DiKotra, LakshmiWang, FazuoKotra, Lakshmi P.Wang, FenglaiKotsiantis, SotirisWang, FukeKotsuchibashi, YoheiWang, FupengKoubogiannis, Dimitrios G.Wang, FuyiKoudounas, KonstantinosWang, GuangshunKoufaki, MariaWang, GuankuiKoumoulos, Elias P.Wang, GuifangKouretas, DimitriosWang, GuliangKoutahzadeh, NeginWang, HsiuyingKoutelidakis, Antonios E.Wang, HuKoutentis, Panayiotis A.Wang, HuaishanKoutsogiannis, IoannisWang, HuanchenKouznetsova, ValentinaWang, HuiKovačević Ganić, KarinWang, Hui-ChunKovačević, BorislavWang, Hui-minKovačević, DavorWang, HuxuanKovačević, StrahinjaWang, JeffreyKovacheva, DanielaWang, JiabinKovacs, LajosWang, JianKovacs, RichardWang, JianboKovács, ZoltánWang, JianhuaKoval, AlexeyWang, JinKovalenko, SergeyWang, Julie Tzu-WenKovalevsky, Andrei V.Wang, JunKovar, PetrWang, KaiKováts, ÉvaWang, KankanKovbasnjuk, OlgaWang, LiKowal, KrzysztofWang, LingKowalczewski, PrzemysławWang, LipingKowalczuk, DorotaWang, LiqiangKowalczyk, AleksandraWang, LirongKowalczyk, DariuszWang, LuKowalczyk, IwonaWang, LuyuKowalczyk, MariuszWang, Michael CaiKowalczyk, TimWang, Mindy Y.Kowalczyk, TomaszWang, Ming-FuKowalska, EwaWang, NanKowalska, IwonaWang, NianKowalska, JoannaWang, Pei-MingKowalska, KarolinaWang, PengKowalska, KatarzynaWang, PengchengKowalski, KonradWang, PingyuanKowalski, RadosławWang, QingdeKowol, Christian R.Wang, QingsongKowolik, ClaudiaWang, QunKoyack, MarcWang, RuiyongKoyioni, MariaWang, San-LangKozak, YoramWang, Sheng-ShihKozanecki, MarcinWang, ShenqiKozempel, JánWang, ShihuaKoziak, KatarzynaWang, Shin-WeiKozieł, EdmundWang, ShueKoziel, JacekWang, ShyiwuKoziol, MateuszWang, SonglinKoziróg, AnnaWang, TaoranKoźlecki, TomaszWang, TengfeiKozlevčar, BojanWang, Tong-HongKozliak, Evguenii I.Wang, Tzu-FanKozlík, PetrWang, WeiKozlov, Mikhail V.Wang, WenjingKozlov, SergeyWang, XiaopingKozlova, EkaterinaWang, XiaoxinKozlova, ElenaWang, XiaoyongKozlowska, JustynaWang, XinkunKozłowska, MariolaWang, XinwenKoźmiński, WiktorWang, XizuKragović, MilanWang, XuKrajka-Kuźniak, ViolettaWang, YanbinKrakhalev, Mikhail N.Wang, Yeng-TsengKrakowiak, AgnieszkaWang, YifeiKrakowiak, JoannaWang, YouxinKrálovec, KarelWang, YuKranz, MathiasWang, YueshengKrasavin, MikhailWang, Yun-MingKrasikova, Raisa N.Wang, ZhenxinKrasnoslobodtsev, Alexey V.Wang, ZhihanKratky, LukasWang, ZhijunKrátký, MartinWang, ZhongyangKratochvilova, IrenaWang, ZongjieKraus, ChristophWard, JohnKrause, FrankWard, Michael D.Krause, GünterWare, VassieKrawczyk, JoannaWarren, J. DavidKrchnak, ViktorWarren, Warren S.Kreis, WolfgangWaser, MarioKrela-Kaźmierczak, IwonaWasilewski, TomaszKřen, VladimirWasylka, Justyna PłotkaKrenn, LiselotteWatanabe, RyuichiKressler, JörgWaterhouse, AndrewKreuter, JustinWatkins, A. NealKrga, IrenaWatkins, Gordon LeonardKriegsmann, JörgWatzky, MurielleKrikstolaityte, VidaWawrzyniak, JolantaKrisch, JuditWdowin, MagdalenaKrishna, GokulWebb, IanKrishnakumar, ShyamWebba Da Silva, MateusKrishnan, KrishWeber, BirgitKrissinel, EugeneWeber, JohnKristensen, Tor ErikWedekind, Joseph E.Krisyuk, VladislavWeeks, BrandonKritikos, GeorgiosWeghuber, JulianKritsi, EftichiaWegrzyn, GrzegorzKrivoruchko, AnastasiiaWei, CunxuKroeker, S.Wei, Da-HuaKrokidis, MariosWei, GangKrokosz, AnitaWei, HuaKról, EwelinaWei, JianjunKrólicka, AgnieszkaWei, KongchangKronekova, ZuzanaWei, WanxingKručaitė, GintarėWei, YufengKrueger, Joanna K.Wei, ZhaojunKruger, CherieWei, ZhongbaoKruger, Jacob S.Weidner, SteffenKrukiewicz, KatarzynaWeigand, WolfgangKrumkacheva, Olesya A.Weina, Peter J.Krummenacher, ClaudeWeinberger, ChristianKrumova, SashkaWeis, MartinKruse, MartinWeiss, RichardKruszynski, RafalWeiss, SiegfriedKrysinski, PawelWelch, KevinKryštof, VladimírWelker, MarkKryštofová, SvetlanaWeller, Michael G.Krystyna, Skalicka-WoźniakWells, TracyKrzeminski, MarekWen, XiaofengKrzywiecki, MaciejWen, ZhiweiKrzyżak, Artur TadeuszWeng, Ching FengKsenofontov, AlexanderWenta, TomaszKshirsagar, Prakash G.Wente, NicoleKu, Kang-MoWenzel, BarbaraKu, SeockmoWerlé, ChristopheKuan, Yu-HsiangWesołowski, MarekKubala, LukášWessel, Gary M.Kubatka, PeterWest, James D.Kubatzky, KatharinaWestcott, Stephen A.Kubica, PawełWeston, PaulKubicek, Christian PeterWestphal, AdrieKubíček, VojtěchWestwell, AndrewKubíčková, AnnaWhite, PeterKubina, RobertWhite, Robert L.Kubinová, RenataWhite, RosemaryKubinyi, MiklósWhyte, ClaireKubissa, WojciechWianowska, DorotaKubo, Anna-LiisaWibowo, DavidKubo, AtsushiWicker, LouiseKubo, MasatakaWiczkowski, WieslawKubo, MiwaWidenmeyer, MarcKubo, TakanoriWidomska, JustynaKubo, YoshinaoWieczorek, MarcinKuca, KamilWieczorek, PiotrKuča, KamilWieczorek, Piotr P.Kučera, LukášWieczorek, Rafal M.Kučera, TomášWieczorek, RobertKučerik, JiříWiedman, GregoryKucerova, MartaWielgomas, BartoszKucharska, KarolinaWiener, ReuvenKucinska, MalgorzataWieszczycka, KarolinaKuckling, DirkWietrzyk, JoannaKućko, AgataWietstock, PhilipKucuk, IsrafilWigle, JeffreyKuczera, KrzysztofWiglusz, KatarzynaKudasik, MateuszWiktor, ArturKuddannaya, ShreyasWilhelm, ReneKúdela, JozefWilkinson, JennyKuder, TomaszWilkowska, AgnieszkaKudłak, BłażejWillaert, RonnieKudo, FumitakaWillers, ClarissaKudo, YutaWilliams, AntonyKudr, JiriWilliams, LyndaKudryavtsev, Denis S.Williams, Noelle S.Kuehne, IrinaWilliams, VanceKueppers, StephanWilliamson, VernellKues, Wilfried A.Willmore, William G.Kühl, Anja A.Wills, MartinKühn, OliverWilner, Samantha E.Kujawska, MałgorzataWilson, Anne M.Kujawski, JacekWilson, Danny W.Kujawski, WojciechWilson, Jeffrey M.Kukli, KaupoWilson, Justin J.Kukovec, Boris-MarkoWilson, PeterKukula-Koch, WirginiaWilson, Philippe B.Kula, KarolinaWilton, SteveKula, SławomirWimmer, ZdenekKula-Pacurar, AnnaWinckler, ThomasKulbacka, JulitaWindholz, LaurentiusKulczyński, BartoszWindshügel, BjörnKulda, VlastimilWink, MichaelKulichikhin, ValeryWińska, KatarzynaKulkarni, Jayesh A.Winter, Horst HenningKulkarni, ShashankWiraja, ChristianKulma, MagdalenaWisniewski, MarekKulnitskiy, Boris A.Wiśniewski, MarekKulozik, UlrichWitczak, CarolKulp, SamuelWitczak, MariuszKumagai, YuyaWitczak, Zbigniew J.Kumal, RajuWitkowski, ArturKumamoto, EiichiWitt, KatarzynaKumar Patel, SravanWitte, MartinKumar, AnujWłodarczyk, MaciejKumar, DhirajWłodarczyk, ZbigniewKumar, DushyantWłodarczyk-Stasiak, MarzenaKumar, HirdeshWnuk, AgnieszkaKumar, Keshav SenthilWnuk, MaciejKumar, KrishanWnuk, StanislawKumar, Majeti N.V. RaviWoelwer-Rieck, UrsulaKumar, ManuWojaczyńska, ElżbietaKumar, NareshWójciak, MagdalenaKumar, NirbhayWojciechowski, KamilKumar, PankajWojcieszak, RobertKumar, PenmetchaWojcik, CezaryKumar, PradeepWojdylo, AnetaKumar, RaviWojnarowicz, JacekKumar, SantoshWojtkielewicz, AgnieszkaKumar, ShailabhWójtowicz, AgnieszkaKumar, ShaileshWojtunik-Kulesza, Karolina A.Kumar, SujeetWojtunik-Kulesza, Karolina AnnaKumar, SuneelWołejko, ElżbietaKumar, VijayWolf, ChristophKumi-Diaka, JamesWolf, KatarinaKun, LiWolff, MarcusKuncewicz, JoannaWolff, MaxKung, Chung-WeiWölfle, UteKung, Woon-ManWolfson, AdiKunji, Edmund R.S.Wolny, Juliusz A.Kunsági-Máté, SándorWołowicz, AnnaKunz, HorstWołowiec, StanisławKunz, WolframWolter, TilmanKuo, Chia-HungWon, Kyung JongKuo, Dong-HauWon, Moo-HoKuo, Meng I.Won, SeunggunKuo, Ping-ChungWondrak, GeorgKuo, ShiaoweiWong, AlanKuo, Tsung-RongWong, ChungKuo, Wen-TenWong, Clarence T. T.Kuo, Yao-HaurWong, Fung-FuhKupka, TeobaldWong, MauriceKuppusamy, Senthil KumarWong, Wai-YeungKurańska, MariaWong, Yung HouKurasaki, MasaakiWoo, Sun HeeKurc, BeataWood, Troy D.Kurczab, RafałWoscholski, RudigerKurdziel, MagdalenaWöstemeyer, JohannesKurien, Biji T.Wowalczyk, WioletaKurihara, HideyukiWozniak, JarosławKuroboshi, ManabuWoźniak, ŁukaszKuroda, DaisukeWoźniak-Budych, MartaKuroda, TakayoshiWozniak-Knopp, GordanaKuroiwa, KeitaWright, Gareth S. A.Kurreck, Prof JensWrobel, RafałKursula, PetriWróbel, TomaszKurtán, TiborWrobel, Tomasz P.Kurtenbach, StefanWróblewska, AgnieszkaKurzatkowska, KatarzynaWróblewski, KarolKurzawa, MarzannaWu, AlanKusaczuk, MagdalenaWu, BinKustov, LeonidWu, CenKustrin, SnezanaWu, ChaoKustrowski, PiotrWu, Chiao-EnKusukawa, TakahiroWu, Chia-WenKutner, WlodzimierzWu, Chieh-HsiKuttel, MichelleWu, Chi-ReiKuttner, ChristianWu, ChongmingKuwabara, TakuyaWu, Chun-ChiKuwajima, KunihiroWu, Chung-ChunKuwatani, TatsuWu, Chung-HsinKuzhir, PolinaWu, DiKuzmin, AlexeiWu, Fu-GenKuznetsov, Maxim L.Wu, HongjingKuznetsov, NikitaWu, Ho-ShingKuźnik, NikodemWu, Hung-ChinKuzuhara, DaikiWu, Jian-YongKuzuya, AkinoriWu, Jing-YunKvasnica, MiroslavWu, Jin-YiKwak, Jong HwanWu, KaiyuKwak, SeonyeongWu, KejunKwan, Hiu-yeeWu, Li-chenKwaśniewska, AnitaWu, LiliKwasniewski, Misha T.Wu, LixinKwiecień, IwonaWu, MaochunKwok, Hang FaiWu, MinKwok, Henry Hang FaiWu, Ming-JiuanKwon, Hyuk-JunWu, Richard YouKwon, Hyung JooWu, RuilianKwon, JaeyulWu, Shan-YingKwon, Jung-HwanWu, ShengruKwon, Seong JungWu, ShengYunKwon, So HeeWu, Shih-JehKwon, Soon-HongWu, Shu-JuKwon, Sung WonWu, XinKwon, SunohWu, XuedanKwon, YongchanWu, XuejianKwon, Young M.Wu, Yang-ChangKwon, YoungjooWu, YunlongKwon, Young-WanWu, Yu-TseKyoungsik, ChangWu, Yu-WeiKyriakopoulou, KonstantinaWu, ZuchengKyzas, GeorgeWulff, Jeremy E.Kyzas, George Z.Wuttke, StefanKyzioł, AgnieszkaWybraniec, SławomirL. Pey, AngelWylie, Benjamin J.L. Waters, MarceyWyman, IanL’homme, LaurentWyszkowski, MirosławLa Clair, JamesXavier, AlencarLa Flor, Silvia DeXavier, Ana Maria Rebelo BarretoLa Foresta, FabioXavier, Cristina Pinto RibeiroLa Motta, ConcettinaXavier, Nuno M.La Penna, GiovanniXi, XinpingLa Regina, GiuseppeXia, AndongLa Rosa, CarmeloXia, ChaomingLa Spina, MartinaXiang, ChunhuiLabbé, SimonXiang, PingLabbozzetta, ManuelaXiang, Shi-huaLabrou, NikolaosXiang, XiaoqiangLacaille-Dubois, Marie-AlethXiao, KaidaLacal, Juan CarlosXiao, SunLachenmeier, DirkXiao, ZhengtaoLachman, JaromirXiao, ZhoushengLachowicz, SabinaXie, BingxianLachter, Elizabeth R.Xie, FengweiLackie, Peter M.Xie, JingLacko, AndrasXie, KefengLackovic, IgorXie, WankunLacomme, ChristopheXie, Wei-dongŁączkowski, Krzysztof Z.Xie, WenyanLadavos, Athanasios K.Xie, Zhong-RuLade, HarshadXin, DongyueLafond, MickaëlXing, ChenLafont, RenéXing, FeiLafrenie, Robert M.Xing, YalanLaganà, Antonio SimoneXiong, JiaLaghi, LucaXodo, LuigiLago, Ana B.Xu, AirongLago, João Henrique GhilardiXu, ChungangLagoa, RicardoXu, DongLagoumintzis, GeorgeXu, FengLagravère, Manuel O.Xu, FuchaoLai, Chian-HuiXu, HongtaoLaitinen, RiikkaXu, JialiangLaitinen, RistoXu, JianweiLakatos, BorisXu, JiaxiLakowski, TedXu, JiminLakshmanan, ImayavarambanXu, JinbinLalas, StavrosXu, JunLalatsa, AikateriniXu, Jun-LiLam, KevinXu, KangzhenLam, Sio-HongXu, KeLama, AdrianoXu, KuiLamac, MartinXu, LinruLamari, Fotini N.Xu, LuLamas, AlexandreXu, NanLambert, JoergXu, PeishengLambert, KarenXu, PengtaoLambertucci, CatiaXu, RongLambri, Osvaldo AgustínXu, WeiLambrinidis, GeorgeXu, Wen TaoLambruschini, ChiaraXu, Yan-MingLamers, ChristinaXu, Yi-JunLamichhane, RajanXu, YitingLamouroux, EmmanuelXu, Zhen-LiangLamponi, StefaniaXu, ZhiminLamprou, Dimitrios A.Xuan, Tran DangLamuta, CaterinaXue, DongfengLamy De La Chapelle, MarcXue, GuangaiLamy, ElsaYadav, DhananjayLamy, EvelynYadavalli, Vamsi KLan, YuchengYaghmur, A.Lanças, FernandoYagi, NaotoLanceros-Mendez, SenentxuYahagi, TadahiroLandon, Chelsea DawnYajima, MamikoLange, HeikoYajima, ShunsukeLangelaan, David N.Yakhvarov, DmitryLangendorf, ChristopherYakimova, LuidmilaLanger, ThierryYakovlev, VasilyLanghansova, LenkaYakushev, VladimirLaparra, Jose MoisésYakymovych, AndriyLapcík, OldrichYakymovych, IhorŁapiński, AndrzejYallapu, MuraliLapteva, MariaYamada, HiroyukiLaraia, LucaYamada, ShigeyukiLara-Sanchez, AgustinYamagishi, TakehiroLaratta, BrunaYamaguchi, MakotoLaricchiuta, AnnaritaYamaguchi, MasahikoLarin, SergeyYamaguchi, MasakiLaRocque, JanYamaguchi, SatoshiLaronze-Cochard, MarieYamaguchi, SoichiroLarraneta, EnekoYamaguchi, YukiLarson, ÅsaYamakoshi, HiroyukiLarson, Nicholas B.Yamamoto, TsuyoshiLartigue, LenaicYamamoto, YasunoriLasfar, AhmedYamamoto, YusukeLash, Timothy D.Yamamura, SoichiroLaskowska, MagdalenaYamanaka, ShusukeLaskowski, ŁukaszYamaoka, ToshimitsuLasseuguette, ElsaYamaotsu, NoriyukiLassi, UllaYamato, TakehikoLatek, DorotaYamauchi, AkiraLatella, GiovanniYamauchi, SatoshiŁatka, LeszekYamayoshi, AsakoLatorre-Moratalla, Mariluz LuzYamazaki, YasuomiLatowski, DariuszYamijala, Sharma S. R. K. C.Lattorff, H. Michael G.Yan, DayunLattuada, MarcoYan, HongbinLau, ChunghoYan, JingLaube, MarkusYan, LiangLauberts, MarisYan, LifengLäuchli, SeverinYan, MaryLaudadio, EmilianoYan, ShengLauersen, KyleYan, Yong-BinLaughton, Charles A.Yan, YunfengLauren E., BrownYanase, EmikoLaurenti, EnzoYanda, MuraliLaurini, ErikYáñez, RemediosLavela, PedroYáñez-Vilar, SusanaLavilla, RodolfoYang, Angela Wei HongLavine, BarryYang, BoLavoie, SergeYang, Chen I.Law, RubyYang, Chih-ChungŁawniczak, ŁukaszYang, Chul-SuLaws, Edward A.Yang, ChunlongLay, Horng-LiangYang, ChunzhangLazarides, TheodoreYang, Ding-YahLazzara, GiuseppeYang, Feng-LingLazzarato, LorettaYang, FengqingLazzari, MassimoYang, GuangLazzaroni, RobertoYang, GuangyuLe Borgne, MarcYang, HaifengLe Gall, GwenaelleYang, HoichangLe Grognec, ErwanYang, HongshunLe Ouay, BenjaminYang, JialeLe Pogam, PierreYang, JianxiaoLe Quéré, Jean-LucYang, JieLe Questel, Jean-yvesYang, JingLe Roes-Hill, MarilizeYang, KejiaLe Thanh-Blicharz, JoannaYang, KunLe, T. T. YenYang, LijiangLeabu, MirceaYang, LiqunLeadbeater, NicholasYang, Pei-MingLeahu, AnaYang, RuiLeal, Ana MendesYang, Seung HwanLeao, JoséYang, Shih ChunLeardini, FabriceYang, Shun-ChinLea-Smith, DavidYang, Sung HoLeavesley, DavidYang, Tsung-ShiLebeau, BénédicteYang, WeiLebedeva, Olga E.Yang, Wei-hsiungLebedyeva, IrynaYang, Wen-ChaoLeber, BettinaYang, XinLebreton, JacquesYang, XinghongLebron, José AntonioYang, XinmaiLebrun, VincentYang, XiuweiLecamp, LaurenceYang, XuanLech, KrzystofYang, YangLech, KrzysztofYang, YingLechel, AndreYang, Ying-YingLecian, Orchidea MariaYang, Yu-ChiaoLeclercq, LoicYang, Yu-liangLecomte, PhilippeYang, ZhaogangLederkremer, Gerardo Z.Yang, ZhigangLedesma, Ana EstelaYannarelli, GustavoLedo, AnaYano, MasatoLee, Chang HoonYanshole, Lyudmila V.Lee, ChangguYao, GuangminLee, Che-HsinYao, Hsien-TsungLee, Cheng-IYao, JianLee, Chia-HungYao, MinLee, Chiang-WenYao, Zi-JianLee, Chien-HsingYaoita, YasunoriLee, Ching-KuoYaraki, Mohammad TavakkoliLee, ChongYaremenko, Ivan A.Lee, Choong HwanYaron, Jordan R.Lee, Chun-LinYashchenok, AlexeyLee, Dae YoungYasin, SohailLee, Dae-HeeYasuda, KaoriLee, Do YupYasuda, TakakoLee, DonghyunYasuhiko, KogaLee, Dong-kiYasukawa, KiyoshiLee, DongkyuYasutake, YoshiakiLee, Dong-UnYates, MatthewLee, Han EolYatoh, ShigeruLee, Hee JaeYatom, ShurikLee, Hon ManYatsunyk, LiliyaLee, Hong-KiYavin, EylonLee, Huei-JaneYazaki, RyoLee, Hwa-JinYazlovitskaya, Eugenia M.Lee, Hye SukYE, EnyiLee, HyunYe, HailongLee, Ik JaeYe, Jian-HuiLee, I-TaYe, JianqiaoLee, JaehwiYe, JinxingLee, JeongmiYe, JunLee, Jetty Chung-YungYe, QingLee, JihyunYe, Shu-fengLee, Jin HyungYe, YunpengLee, Jin-KooYedjou, Clement G.Lee, Jin-KyunYeh, Chen-YuLee, Jiunn HawYeh, I-JuLee, Ji-WoongYeh, Jwu-LaiLee, Jong-DaeYeh, Ting-fengLee, Joon KyuYeliseev, AlexeiLee, Jue-YeonYelko, Rodríguez-CarrascoLee, Jun SikYen, Chia-HungLee, Jung RoYen, Feng-LinLee, Jung SeokYen, Frances T.Lee, JunseongYen, Yi-KuangLee, Ki-TeakYeo, SynLee, Kun-MuYeoman, CarlLee, KyungminYepuri, Nageshwar R.Lee, Li-TingYi, MyunggiLee, Meng-shiouYi, Sun-ShinLee, Min WookYi, TaoLee, MinaYi, YougenLee, Sang GilYi, Young-SuLee, Sang KookYihao, ChenLee, Sang-HanYin, ChungenLee, Sang-hunYin, HengLee, SejoonYin, HengfuLee, Seung JaeYin, KaiLee, SeungguYin, KingsleyLee, Sheng-LongYin, PanchaoLee, Shiow-JuYin, ShengLee, Shyi-LongYin, ZhouyangLee, SukmookYo, TanakaLee, Sung KeunYohda, MasafumiLee, SungyulYokomatsu, TsutomuLee, Tae JinYokosuka, AkihitoLee, Tse-MinYokosyo, KengoLee, Tzu-TaiYokota, ShingoLee, VictorYokoyama, ChikakoLee, Woo-KyungYong, Kar WeyLee, Yeon-JuYong, Yang-chunLee, YongbokYoo, Dong JinLee, Yu-HsuanYoo, DongwonLee, Yun-SangYoo, Hah YoungLee-Ruff, EdwardYoo, Jung SunLees, JustinYoo, So YoungLeeth, CarolineYoon, Byung-IlLefèvre, GuillaumeYoon, IlLefèvre, ThierryYoon, In SooLeffler, HakonYoon, Kee-DongLegaz González, María EstrellaYoon, Moon-YoungLéger, BastienYoon, Seong-JunLegon, AnthonyYoon, Tae JunLegrand, François-XavierYoon, YeojoonLegrand, VincentYoshida, EriLegros, JulienYoshida, KentaroLeherte, LaurenceYoshida, Nidia CristianeLehnherr, DanYoshida, TakashiLehocky, MarianYoshihara, EijiLehtonen, AriYoshimatsu, MitsuhiroLei, TingYoshimi, YasuharuLei, ZhentianYoshimitsu, TakehikoLeidenheimer, NancyYoshimoto, Francis K.Leisz, SandraYoshimune, MikiLeitão, Jorge H.Yoshimura, TomokazuLeitao, Ruben ElvasYoshinaga, JunLeitinger, BirgitYoshinari, NobutoLekka, MalgorzataYoshino, HironoriLelešius, RaimundasYoshioka, Ken-ichiLelli, MorenoYoshioka, TaiyoLemaire, MartinYou, Hye JinLemanowicz, MarcinYou, JungmokLemercier, GillesYou, SeungkwonLemli, BeataYou, ZongbingLemordant, DanielYoung, MichaelLen, ChristopheYousefi, Behrooz H.Lenaic, CouedelYoussef, HibaouiLenardao, EderYperman, JanLenaz, GiorgioYu, FabiaoLenci, ElenaYu, HuaLenhard, Justin R.Yu, JaecheulLennartz, MichelleYu, Jae-sikLentini, GiovanniYu, JingangLentz, ChristianYu, JingxianLenucci, Marcello SalvatoreYu, KangLenz, ChristofYu, LeixiaoLenzi, MarceloYu, LiangLeon Serrano, JavierYu, LingLeon, FranciscoYu, LuLeón, JosefaYu, MeihuaLéonard, EstelleYu, RongminLeonard, StephenYu, Sheng-ShengLeondiadis, LeondiosYu, XiaobinLeonelli, FrancescaYu, YanboLeong, David T.Yu, YuLeong, Max K.Yu, ZuorenLeoni, AlbertoYuan, BingLeonidas, Demetres D.Yuan, Wei-EnLeonidas, PalilisYuan, XinxuLeonowicz, ZbigniewYuan, YaxiaLeón-Rivera, IsmaelYuan, ZhaoyangLeontopoulos, StefanosYuan, ZhihongLeopold, LoredanaYuasa, HideyaLeow, Melvin Khee-ShingYuasa, MariLepareur, NicolasYue, MinLephart, Edwin D.Yue, XuyiLeporatti, StefanoYue, YoufengLepore, MariaYukl, Erik T.Leporini, MariarosariaYuldashev, Shavkat U.Leprince-Wang, YaminYun, Bong-SikLequin, OlivierYun, ChawonLes, FranciscoYun, KyusikLesgards, Jean-FrançoisYun, MiyongLeshchenko, Elena V.Yung, Hong-WaLeśniewicz, AnnaYurchenko, EkaterinaLešnik, SamoYurchenko, VyacheslavLesnikowski, Zbigniew J.Yushina, Irina D.Lesov, IvanZaarour, MoussaLessard, BenoitZabetakis, IoannisLesyk, Roman B.Zabierowski, PiotrLete, EstherZabihi, OmidLetek, MichalZaccone, ClaudioLeung, Chung-HangZacconi, FlaviaLeung, IvanhoeZachariadis, GeorgeLeung, KaHoZacharis, Constantinos K.Leutou, Alain SimpliceZacharof, MyrtoLevchenko, IgorZadeike, DaivaLeveneur, JeromeZafrilla, PilarLevente Nagy, CsabaZaghib, KarimLević, StevaZagórska, AgnieszkaLevina, AvivaZagotto, GiuseppeLevine, MindyZahari, VinarovLevy, MelZaharia, ValentinLevy-Ontman, OshratZahid, MalihaLewandowicz, GrażynaZahínos, Emilio ViñuelasLewandowska, KatarzynaZaid, HilalLewandowski, WiktorZaidi, SaheemLewin, MattZaini, PauloLewinska, AnnaZairov, RustemLewis, PhillipZaitone, Sawsan A.Lewkowicz, AnetaZając, WojciechLeyva-Jimenez, Francisco JavierZakharova, LuciaLezot, FredericZakinyan, ArthurLezsovits, FerencZakłos-Szyda, MalgorzataLhiaubet, Virginie LyriaZakoldaev, Roman A.Li, AifengZakrevsky, PaulLi, Bing-YanZakrzewska, MalgorzataLi, ChengxiZakrzewski, JerzyLi, DongZalibera, MichalLi, DongmeiZaltariov, Mirela-FernandaLi, FengZałuski, DanielLi, FenghuaZamaraewa, MariaLi, FuZamaratskaia, GaliaLi, Fu-shuangZamay, TatianaLi, GangZambito, YleniaLi, GuangmingZamborine-Nemeth, EvaLi, GuannaZambrano-Zaragoza, Maria L. Li, GuohuiZamfir, Carmen LăcrămioaraLi, HaolongZammit-Mangion, MarionLi, HeŻamojć, KrzysztofLi, HongdaZamora-Ros, RaulLi, Hou-JinZamyatnin, AndreyLi, Hsing-HuiZanardi, EmanuelaLi, HuZandvliet, HaroldLi, HuaZanetti, StefaniaLi, HuabinZangrando, EnnioLi, HuiZani, LorenzoLi, JianfengZannetti, AntonellaLi, JianmeiZanolla, MarianelaLi, Jiao JiaoZanotti, GiuseppeLi, JinganZapata, PaulaLi, JingpengZapico, Sara C.Li, JinpengZappia, GiovanniLi, JinyuZaprutko, LucjuszLi, JunjieZaptata-Torres, Molecular Modeling GeraldLi, JunrongZarabadi-Poor, PezhmanLi, KefengZaragoza Contreras, Erasto ArmandoLi, Kuo-TsengZareba, Jan K.Li, LanZarejousheghani, MashaalahLi, LiZaremba-Czogalla, MagdalenaLi, LianboZarkovic, NevenLi, LiyaŻarowska, BarbaraLi, MengxingZarrelli, ArmandoLi, Ming-YuZarzycki, Pawel K.Li, MingzhongZarzyka, IwonaLi, Po-HsienZastrow, Melissa L.Li, Qian-ShengZatterale, FedericaLi, QunZavala-Rivera, PaulLi, RachelZawada, KatarzynaLi, RonghuiZawierucha, IwonaLi, Ruei-NianZbancioc, GheorghiţăLi, SihengZdanowicz, MagdalenaLi, TaoTaoZdarta, JakubLi, TiangangZdenek, ChristinaLi, TianhuZechel, StefanLi, WeiZedler, ŁukaszLi, Wen-WuZeeshan, MuhammadLi, WenyiZehl, MartinLi, WenyueZeković, ZoranLi, XianŻelaszczyk, DorotaLi, Xiang-GuoZeleny, ReinhardLi, XianglinZelkó, RománaLi, XiangmeiZeng, BijunLi, XiangnanZeng, HuipingLi, XiaoZeng, JiaLi, XiaojunZeng, TaoLi, XiaotaoZeng, WeiguangLi, XinminZeng, XiangkangLi, XinmingZengin, GokhanLi, XueZeni, OlgaLi, XundeZepeda, Rossana C.Li, YanZeremski, TijanaLi, YangŻesławska, EwaLi, YaozongZestos, Alexander G.Li, YingZettsu, NobuyukiLi, YiwenZha, GaofengLi, YuanzuoZhai, KuiLi, YubinZhan, PengLi, YunguoZhan, WenboLi, YuxinZhan, XuanzhiLi, ZhaohuiZhang, BoLi, ZhengqiangZhang, CankuiLi, ZhiZhang, ChaoLi, Zhi-MinhgZhang, ChengbinLi, ZhiyuZhang, ChunhuiLiagre, BertrandZhang, DongmingLiakos, IoannisZhang, DongzhouLiang, ChenZhang, EnLiang, HaoZhang, FanLiang, HaozhaoZhang, FumingLiang, Pi-HuiZhang, GuannanLiang, Po-HuangZhang, GuoqiLiang, WenyanZhang, HongboLiang, XianruiZhang, HongyiLiang, YongZhang, HuLiang, YuerongZhang, HuaweiLiang, ZhibinZhang, HuiyingLiao, Hsiao-WeiZhang, JianyingLiao, Jiunn-DerZhang, JianyongLiao, Jyh-feiZhang, JieLiao, Ling-HsiuZhang, JinLiao, PanZhang, JingJingLiao, PengZhang, JinweiLiao, Shu-ChuanZhang, JunboLiao, WupengZhang, JunliangLiao, Yi-HungZhang, JunxianLiao, Ying-ChihZhang, JunzengLiao, YonghongZhang, KaiLiaudanskas, MindaugasZhang, KaihuanLiaw, Chih-ChuangZhang, LeiLiberto, EricaZhang, LiLicata, PatriziaZhang, LiangrenLicea, AlexeiZhang, LiqiangLicini, CaterinaZhang, PengLickiss, PaulZhang, QianLidon, FernandoZhang, QiangzheLiebman, JoelZhang, QichunLieder, BarbaraZhang, QingruiLiefeith, KlausZhang, Qing-WenLigaray, MayzoneeZhang, QiongLigat, GaetanZhang, RunLigor, MagdalenaZhang, ShanjuLiguori, AngeloZhang, Shuang-QingLikhacheva, Anna YuZhang, TaoLikodimos, VlassiosZhang, TongLiktor-Busa, ErikaZhang, WeiLim, Eun-KyungZhang, WenLim, JasonZhang, WencaiLim, Ji-HongZhang, WenjunLim, JiseokZhang, WenxuanLim, Ki-TaekZhang, WenzhongLim, KyuZhang, XiangLim, Lee WeiZhang, XiaoningLim, Lina H. K.Zhang, XiaoyuLim, Sang MooZhang, XinxingLim, SoyeonZhang, XinyuLima Do Monte-Neto, RubensZhang, XuLima Ribeiro, MarceloZhang, XuexinLima, BeatrizZhang, YanchengLima, DênisZhang, Ye-WangLima, Raquel T.Zhang, YinfengLima, Rui A.Zhang, YixuanLima, SofiaZhang, YonghongLimauro, DanilaZhang, YoujunLimbeck, AndreasZhang, YoumingLimongi, TaniaZhang, YuLin, Cheng-WenZhang, YueLin, Cherng-YuanZhang, YumiaoLin, Chih-HsunZhang, YuningLin, GuitingZhang, YuyuLin, HaishuZhang, ZhanhuiLin, HoZhang, ZhenbinLin, Hung-YinZhang, ZhenhuaLin, Jer-AnZhang, ZhidongLin, Kuo-ShyanZhang, ZhiguoLin, LigenZhang, Zhong-XingLin, Long-LiuZhang, ZongtaoLin, Mei-HsiangZhao, BoxinLin, MengZhao, FengyuLin, Po-HengZhao, FuliLin, QishanZhao, JinlingLin, RuibiaoZhao, LinboLin, RuweiZhao, LiyanLin, Shih-YiZhao, NingningLin, ShuoZhao, PeishenLin, Tung-YiZhao, QihuaLin, Tzer-BinZhao, QingnanLin, WeifengZhao, WanxiangLin, YangjuZhao, WeiLin, Yang-weiZhao, WeijieLin, YibinZhao, XiLin, YingboZhao, XianhuiLin, Ying-ChiZhao, XiuhuaLin, ZheshuaiZhao, XuebingLin, ZhiweiZhao, XuhuiLin, ZhoumengZhao, YanchuanLin, ZongtaoZhao, Yong-BiaoLinares, JoseZhao, YuguangLinares, MaríaZhao, YunfengLinares-Pastén, Javier A.Zhao, YuxiaLincoln, RichardZhao, ZengfengLindahl, UlfZhao, ZhenjiangLindberg, GerrickZhao, ZhenwenLinder, MarkusZhdanov, Andrey P.Lindner, MartinaZheleva-Dimitrova, DimitrinaLindquist, NathanZheng, BoLinert, WolfgangZheng, LiqiuLink, AndreasZheng, ShilongLinko, VeikkoZheng, ShuanghaoLinseman, DanielZheng, XingLinul, EmanoilZhiponova, MiroslavaLionetti, VincenzoZhitomirsky, IgorLionetto, Maria GiuliaZhivkova, ZvetankaLiou, Gunn-GuangZhong, Haizhen AndrewLiou, Ying-mingZhongfeng, ZhangLipert, AnnaZhou, BeiyanLipiński, Piotr F. J.Zhou, ChiLipparini, FilippoZhou, DongfangLippens, GuyZhou, GuangfengLips, KatrinZhou, Hai-bingLipscomb, Michael W.Zhou, HuaLis Arias, Manuel J.Zhou, JiajingLiscano, YamilZhou, JianLisec, JanZhou, LiLisi, LuciaZhou, LinliLiška, MarekZhou, MeiLisuzzo, LorenzoZhou, PhoebeLitvić, MladenZhou, QingyuLiu, Bing-LanZhou, QunLiu, BoZhou, ShengbinLiu, ChangZhou, TaoLiu, Chao-LinZhou, TengLiu, ChaoxingZhou, XiaochunLiu, ChengmeiZhou, XiaohongLiu, Chen-GuangZhou, XinglinLiu, Cheuk LunZhou, YanLiu, Chung-ChiunZhou, ZheLiu, ChunmingZhou, ZhidongLiu, DongxiaZhu, CaihongLiu, DongxuZhu, ChenglinLiu, FufengZhu, DandanLiu, FuguoZhu, FengLiu, GangZhu, HuiLiu, Hai-yangZhu, JieLiu, HongbingZhu, JunjieLiu, HongdeZhu, LanLiu, HongyuZhu, LinLiu, HongzhiZhu, QingzengLiu, JiZhu, WeiLiu, JingjingZhu, WeigangLiu, JinmingZhu, WufuLiu, JuewenZhu, XuejunLiu, JunZhu, YuyangLiu, Jun-JenZhu, ZhenbaoLiu, KunZhuang, Shu-linLiu, LiangliangZhuang, YongliangLiu, ManingZhukov, IgorLiu, MiaomiaoZhukova, NataliaLiu, MinminZhuo, Guan-YuLiu, PengdaZiaee, AhmadLiu, PengzhanZiarno, MałgorzataLiu, Shih-JenZiats, Nicholas P.Liu, Shing-HwaZidek, JanLiu, Shiuh-TzungŽídek, LukášLiu, Shi-XiaZidorn, ChristianLiu, ShuangZięba, AndrzejLiu, Shyh-JiunZiegenbalg, DirkLiu, TaihongZielinska, AleksandraLiu, TaoZielinska, DanutaLiu, XianhuZielińska, EwelinaLiu, XinZielińska, SylwiaLiu, Xin-YongZielińska-Błajet, MariolaLiu, XuqingZielińska-Pisklak, MonikaLiu, YangZiembińska-Buczyńska, AleksandraLiu, YangyangZiemkowska, WandaLiu, YanrongZienkiewicz-Strzałka, MałgorzataLiu, Yi-HungZierkiewicz, WiktorLiu, YingZilberg, ShmuelLiu, YingjunZille, AndreaLiu, YongliangZimmerman, WilliamLiu, YongqiangZimmermann, BennoLiu, YouzhongZimnyakov, DmitryLiu, YuZinchenko, AnatolyLiu, YueZingg, Jean-MarcLiu, Yung-ChuanZinicovscaia, IngaLiu, Yu-PengZitko, JanLiu, ZhaominZivanovic, JasminaLiu, ZhaopengZiyatdinova, GuzelLiu, ZhenqiuZizza, PasqualeLiu, ZhiZlatopolskiy, BorisLiu, ZhiqianZloh, MireLiu, ZitongZłotek, UrszulaLivaniou, EvangeliaZlotin, Sergey G.Liwinienko, GrzegorzZmora, PawelLiwo, AdamZmudzki, PawelLizardi-Mendoza, JaimeZnamirowska, AgataLizcano, FernandoZnosko, Brent M.Ljubenkov, IvicaZobel, PatrickLlanos, JaimeZobi, FabioLlata, Marta RuizZoccali, MariosimoneLlop, JordiZoidis, GrigorisLlorca, JordiŻołek, TeresaLloveras, VegaŻołnowska, BeataLluna, Amparo GameroZoltan-Istvan, SzaboLo Faro, Maria JosèZonta, CristianoLo Giudice, AngelinaZoran, StevićLo Sardo, FedericaZoratti, MarioLo Scalzo, RobertoZorc, BrankaLo, Huang-MuZordoky, BeshayLo, SzechengZorov, Dmitry B.Lo, Yi-ChenZou, GangLøbner-Olesen, AndersZoumpoulakis, PanagiotisLobo, Glenn P.Zoumpourlis, VassilisLobón, Natividad ChavesZovko Končić, MarijanaLocatelli, MarcelloZu, YouliLockridge, OksanaZubarev, RomanLodi, AlessiaZubitur, Manoli M.Łodyga-Chruścińska, ElżbietaZubko, MaciejLodygin, EvgenyZubkova, OlgaLoginov, DmitriyZubrienė, AstaLognay, GeorgesZubrik, AntonLognay, Georges C.Zucca, AntonioLohning, AnnaŽugić, AnaLoira, IrisZuk, MagdalenaLoiseau, PhilippeZuluaga, PaolaLoizzo, Monica R.Zuniga, CristalLoizzo, Monica RosaZuorro, AntonioLojou, ElisabethZupko, IstvanLok, Chun NamZuza, EsterLollo, GiovannaZwacka, RalfLomberget, ThierryZwergel, ClemensLomonaco, DiegoZygiridis, TheodorosLomonaco, TommasoŻywicka, AnnaLončar, Mirela BausŻyżelewicz, Dorota

